# High Genetic Diversity of Porcine Sapovirus From Diarrheic Piglets in Yunnan Province, China

**DOI:** 10.3389/fvets.2022.854905

**Published:** 2022-07-07

**Authors:** Xiao Liu, Chunlian Song, Yinghua Liu, Kaixing Qu, Junyu Bi, Junlong Bi, Yunhua Wang, Ying Yang, Junhua Sun, Zhigang Guo, Ganwu Li, Jianping Liu, Gefen Yin

**Affiliations:** ^1^College of Animal Veterinary Medicine, Yunnan Agricultural University, Kunming, China; ^2^Academy of Science and Technology, Chuxiong Normal University, Chuxiong, China; ^3^College of Life Sciences, Nanjing Normal University, Nanjing, China; ^4^Department of Veterinary Diagnostic and Production Animal Medicine, College of Veterinary Medicine, Iowa State University, Ames, IA, United States

**Keywords:** porcine sapovirus, GIII, GV, Yunnan, genetic diversity

## Abstract

As one of the most important enteric viruses, sapovirus (SaV) can infect humans and a variety of animals. Until now, 19 SaV genogroups have been identified, among which 4 from human (GI, GII, GIV, and GV) and 8 from swine (GIII, GV–GXI). Porcine sapovirus (PoSaV) GIII has been prevalent in China; however, the status of PoSaV infection in Yunnan province remains unknown. In this study, 202 fecal samples were collected from piglets associated with outbreaks of acute diarrhea in Yunnan between January and May 2020. PoSaV detection revealed that the total PoSaV infection rate in Yunnan was 35.2%, with 21 PoSaV strains determined and phylogenetically analyzed. The phylogenetic tree analyses demonstrated that twenty PoSaV strains belonged to GIII and fell into five genotypes, whereas one PoSaV strain (YNQB) belonged to GV. Sequence alignments revealed deletions in *VP2* region in 10 of the 20 GIII strains, as well as deletions and insertions in *VP1* region of the GV strain (YNQB). Furthermore, genomic recombination analyses showed that two GIII strains (YNAN and YNJD) were recombinants, closely related to reference sequences MK965898 and LC215880, MK965898 and FJ387164, respectively. In summary, PoSaV-GIII strains were identified in Yunnan in 2020, and for the first time, a PoSaV-GV strain was identified from China, whereas the comprehensive analyses illustrated high genetic diversity of Yunnan PoSaV strains. This study may shed new light on the current PoSaV infections in Yunnan and pave the way toward further control of the PoSaV infections in China.

## Introduction

Sapovirus (SaV) belongs to *Sapovirus* genus of the *Caliciviridae* family and is a positive sense, single-stranded RNA virus. Its genome is ~7.1–7.7 kb in length, containing two open reading frames (ORF1 and ORF2) and a polyA tail at the 3′ end (1). ORF1 encodes a large polyprotein, which comprises seven non-structural proteins (NS1-7) and one major capsid protein (VP1), whereas the ORF2 encodes the minor structural protein VP2 (2). The capsid protein VP1 confers high genetic variability and is closely related to the viral immunogenicity, which thus is a key protein for determination of genetic variation and genotype of SaV (3, 4). Some recent studies reported high genetic variation of *VP2* gene with different deletion patterns (5), which may exert potential effects on the immunogenicity and antigenic epitopes of SaV.

Sapovirus has a wide host spectrum, infecting not only humans (6, 7), but also oysters (8), bats (9), rats (10), dogs and cats (11, 12), chimpanzees (13), and pigs (14). SaV is one of the main pathogens that cause acute gastroenteritis in children and the elderly (1, 15, 16) and was observed for the first time under electron microscopy from the stool samples of children with diarrhea from United Kingdom in 1976 (17, 18). SaV was named after the city of Sapporo, Japan, where the virus was first identified in association with gastroenteritis in 1977 (19). The first porcine SaV (PoSaV) was then isolated from piglet in 1980 and named as Cowden strain (14). Afterward, the prevalence of PoSaV was successively reported in Venezuela (20), Brazil (21), USA (5, 22), Belgium (23), Czechia (24), Denmark (25), Japan (26), South Korea (27), and many other countries, including China (28–36).

At present, based on the *VP1* gene sequences, SaV can be divided into 19 genogroups and 52 subtypes (1, 37, 38), with 4 genogroups (GI, GII, GIV, and GV) and 8 genogroups (GIII and GV–GXI) identified in humans and pigs, respectively. Among the 8 PoSaV genogroups, GIII has been determined as the most predominantly circulating genogroup in swine herds worldwide (39–41), whereas GV is an enteric virus that infects both swine and human, thus posing high risk on public health. In this study, for the first time, we report the detection and genomic characterization of 21 PoSaV strains of high genetic diversity from Yunnan province, China, which is one of the biggest swine-raising provinces in China, and more interestingly, we describe the first case of identification of PoSaV-GV strain from China.

## Materials and Methods

### Sample Collection

During an outbreak of severe diarrhea in 3–50-day-old piglets in Yunnan province, China, between January and May 2020, 202 fecal samples were collected from 53 different pig farms across 15 prefectures/cities of Yunnan province, representing ~5–10% of the total number of farms in Yunnan, in format of rectal swabs using a directed sampling method to minimize cross-contamination, followed by shipment in sterile 15-ml falcon tubes on ice to laboratory for storage at −80°C till isolation of viral nucleic acid and virus detection.

### Extraction of Nucleic Acids and Detection of PoSaV

First, the 202 clinical fecal samples were processed for extraction of viral RNA according to the user instructions of TRIpure total RNA extraction reagent (cat. no.: RP001, BioTeke, Beijing, China). Then, cDNA synthesis of the 202 RNA samples was performed by incubating the reaction at 37°C for 15 min and 85°C for 5 s using EasyScript RT/RI Emzyme Mix (cat. no.: AE311-02, Transgen, Beijing, China) and the downstream primer (5′-CGGTACGCGTAACCAGGGAAAGA-3′ for GIII or 5′-AGTTGTTCATTTYTGGCCATCC-3′ for GV, oligonucleotides purchased from Tsingke Biotech, Kunming, China) for detection of PoSaV-GIII and PoSaV-GV. The subsequent PCR was performed using 2× Phanta Max Master Mix (cat. no.: P505-01, Vazyme, Nanjing, China) and the corresponding prime pairs (Forward 5′-CCCTCATTGGACCAAGTGGGA-3′ and Reverse 5′-CGGTACGCGTAACCAGGGAAAGA-3′ for GIII, Forward 5′-ATCCCAGAGAACATGATGGC-3′ and Reverse 5′-AGTTGTTCATTTYTGGCCATCC-3′ for GV, which were designed according to the reference sequences FJ387164 and KX000383, respectively). The amplification conditions were denaturing at 95°C for 3 min, followed by 35 cycles of 95°C for 15 s, 60°C for 30 s, and 72°C for 60 s, with an additional extension step at 72°C for 5 min. PoSaV-positive samples were determined by gel electrophoresis to visualize the specific amplification products (591 bp and 370 bp in length for the GIII amplicons and the GV amplicons, respectively).

In addition, some most common diarrhea-causing bacterium and parasites, such as *Escherichia coli, Salmonella, Shigella*, and *Coccidia*, were also screened for the PoSaV-positive samples according to the standard protocol. Furthermore, co-infections with other nine diarrhea-related porcine viruses were determined for the PoSaV-positive samples using the primers validated in our recent report (42), namely, porcine sapovirus (PoSaV), porcine epidemic diarrhea virus (PEDV), transmissible gastroenteritis coronavirus (TGEV), porcine rotavirus (PoRV), porcine bocavirus (PBoV), porcine astrovirus (PAstV), porcine deltacoronavirus (PDCoV), classical swine fever virus (CSFV), pseudorabies (PRV), and porcine circovirus 2 (PCV2).

### Sequence Determination of Capsid Protein Genes and Genome of PoSaV

To determine the sequences of capsid protein genes (*VP1* and *VP2*) or genome of PoSaV from the positive samples, the PCR products from the amplifications using the prime pairs listed in Supplementary Table S1 (for PoSaV-GIII strains) and Supplementary Table S2 (for PoSaV-GV strains) were gel purified, cloned into pMD18-T vector (cat. no.: 6011, Takara, Dalian, China), purified, and transformed into *E. coli DH5*α for bulk culture. The plasmid was then extracted from the bacterial culture according to the user instructions of EZ-10 Spin Column Plasmid DNA Minipreps Kit (cat. no.: B610413, Sangon Biotech, Shanghai, China) for subsequent Sanger sequencing confirmation at Sangon Biotech (Shanghai, China). A total of three colonies were sequenced to determine the sequence of *VP1, VP2* or the sequenced genome of PoSaV to minimize the potential sequencing error.

### Sequence Analyses

VP1 is a highly divergent capsid protein and closely related to the viral immunogenicity, and therefore, its encoding gene has been commonly used for genetic characterization of SaV (3, 4). Some selected SaV reference sequences (listed in Supplementary Table S3) were retrieved from the GenBank database for sequence alignments and phylogenetic analyses with the sequences of capsid genes (*VP1* and *VP2*) determined in this study (accession numbers in Supplementary Table S4 and sequences in Supplementary File). DNAStar 6.0 software with the default parameters was used to assemble the sequences and to perform the similarity analysis of the nucleic acid sequences between the selected reference sequences representative of 16 SaV genogroups (Supplementary Table S3) and the 21 strains obtained in this study (Supplementary Table S4 and Supplementary File). Since the alignments contain gaps due to indels, which are phylogenetically informative, the gaps were included into the calculations and further analyses.

The construction of phylogenetic trees from the aligned nucleotide sequences using bootstrap method with 1,000 replicates was achieved using the ClustalW alignment program included in MEGA 7.0 software package (43) with p-distance as the substitution model (22) and using neighbor-joining method. Of note, p distance may not be the best method to estimate genetic distances as the model fails to consider some biologically relevant factors; however, this model is still used in this study, for direct comparison with some previously published similar studies (5, 22). Recombination Detection Program (RDP, version 4) (44) was used to perform recombination analyses using the unweighted pair-group method (UPGMA) (45). All the seven methods (i.e., RDP, GENECONV, BootScan, Maxchi, Chimaera, SiScan, and 3Seq) in the RDP package were included in the recombination analysis, and only when all of them detected a recombination event, can it be accepted as a recombination. The *p*-values reported in the study correspond to the ones from RDP method. Phylogenetic trees and RDP plots are present for each recombinant strain.

## Results

### Detection of PoSaV From Yunnan Province, China

A considerable number of pig farms in Yunnan province reported severe diarrhea in piglets between January and May 2020, which spread in different batches and across adjacent pens, causing huge economic losses to the farms. Over the period, the pig farms collected 202 stool samples in total from diarrheic piglets (3-−6 samples per pig farm) for pathogen diagnoses at College of Animal Veterinary Medicine, Yunnan Agricultural University.

First, we checked the samples for some most common diarrhea-causing bacterium and parasites, such as *Escherichia coli, Salmonella, Shigella*, and *Coccidia*. It turned out that *Salmonella, Shigella*, and *Coccidia* were not detected from the samples. Only *Escherichia coli* were isolated from some of the samples; however, the *Escherichia coli*-killing antibiotics (such as gentamicin and enrofloxacin) from drug sensitivity test did not show noticeable therapeutic effects after being applied to the diarrheic piglets for treatment, indicating that the direct link between *Escherichia coli* infection and diarrhea in piglets was not supported.

Then, RT-PCR detection method showed that the PoSaV-positive rate in diarrheic piglets varied from 22.2 to 64.7% depending on the prefectures or cities, with a total positive rate of 35.2% (71/202, Table 1). In spite of co-infection of PoSaV with some other diarrhea-related porcine viruses, such as PEDV, PoRV, and PAstV, 15 of the 71 PoSaV-positive samples (21.1%) were not co-infected with any of the other nine diarrhea-related porcine viruses, suggesting that PoSaV is a contributing factor for diarrhea in swine herds, in agreement with a very recent report (46). As there is no report yet about PoSaV infection in Yunnan, in this report, we focus on the PoSaV epidemiology and the sequence characterization.

**Table 1:** Sample collection and detection rate of porcine sapovirus.

**Prefecture/City**	**Number of pig farms**	**Sample number**	**Number of PoSaV-positive sample**	**Positive rate** **(%)**
Chuxiong	5	17	11	64.7
Yuxi	6	19	12	63.2
Pu'er	1	4	2	50.0
Kunming	4	15	6	40.0
Lincang	3	9	3	33.3
Xishuangbanna	3	13	4	30.8
Baoshan	3	10	3	30.0
Dehong	3	17	5	29.4
Zhaotong	2	7	2	28.6
Honghe	4	18	5	27.8
Wenshan	3	11	3	27.3
Dali	5	19	5	26.3
Lijiang	2	8	2	25.0
Qujing	7	26	6	23.1
Nujiang	2	9	2	22.2
**Sum**	**53**	**202**	**71**	**35.2**

Not surprisingly, the three prefectures or cities (Chuxiong, Yuxi and Kunming) which are the three biggest swine producing areas in Yunnan province showed highest PoSaV detection rates, when excluding Pu'er city from discussion due to the very limited sample number (only 4 samples in total from Pu'er). Interestingly, besides 70 PoSaV-GIII-positive samples, a stool sample turned to be PoSaV-GV-positive, which is the first case for detection of SaV-GV in pigs from China.

### Genogroup Determination of Yunnan PoSaV Strains by Phylogenetic Analyses

In this study, 2–4 PoSaV-positive samples from each prefecture/city were randomly selected for nucleic acid sequence determination of the structural capsid protein genes *VP1* and *VP2*, or of the sequenced PoSaV genome. Subsequently, nucleic acid sequences of capsid protein genes of 17 GIII PoSaVs, whole genomic sequences of three PoSaV-GIII strains (YNLH, YNAN, and YNJD) and one PoSaV-GV strain (YNQB) were determined using the primer pairs listed in Supplementary Tables S1, S2, respectively. The 21 Yunnan PoSaV strains obtained in this study are documented in GenBank under the accession numbers MW285639–MW285642 and MW296248–MW296266 (Supplementary Table S4 and Supplementary File).

**Figure 1:**
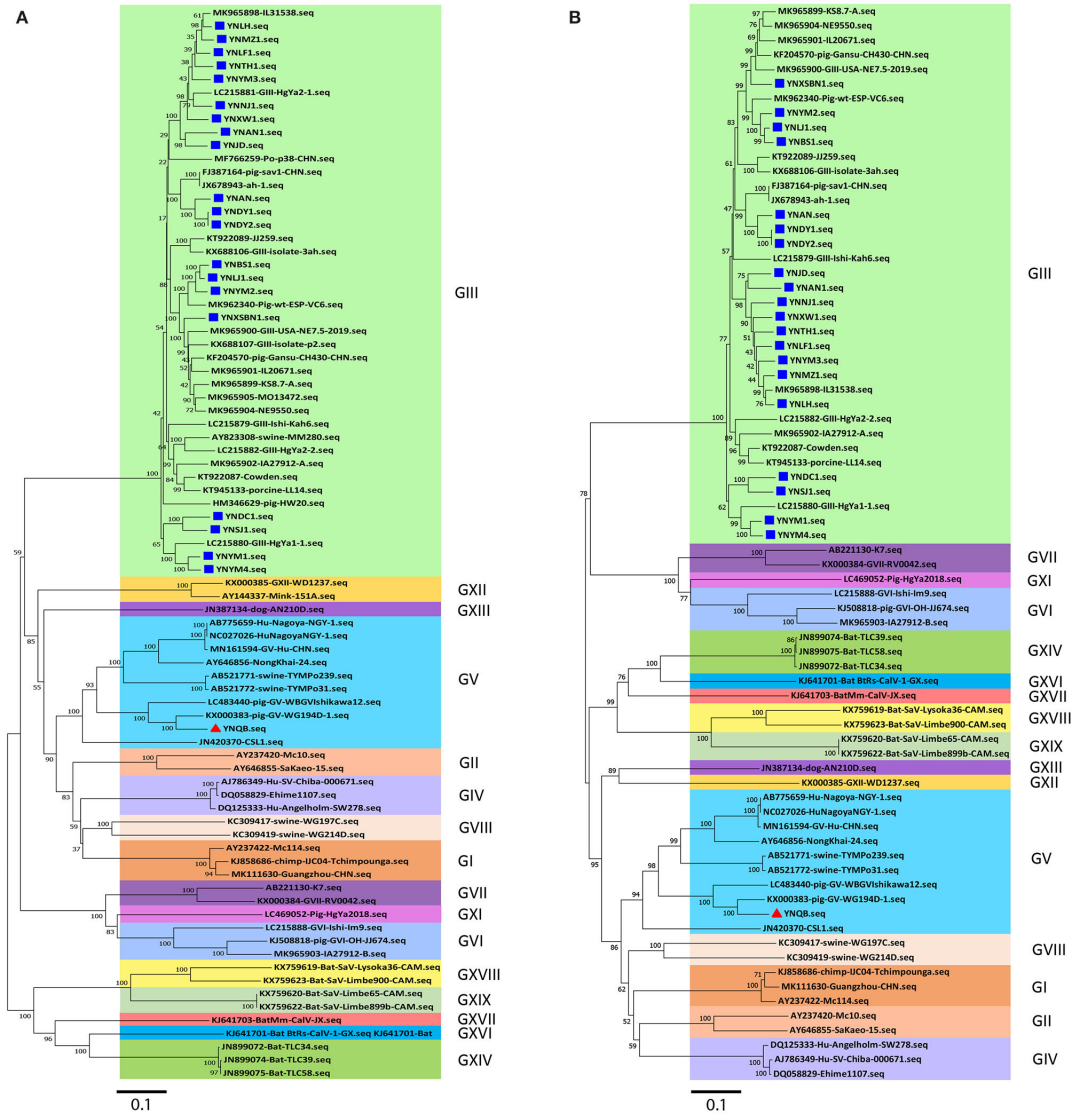
Phylogenetic analysis of sapovirus sequences of *VP1* **(A)** and capsid genes (*VP1* and *VP2*) **(B)** including the 20 Yunnan PoSaV-GIII strains (indicated with a solid blue square) and one Yunnan PoSaV-GV strain YNQB (indicated with a red triangle) determined in this study. The sequences retrieved from GenBank were labeled with their accession numbers (Supplementary Table S3). The phylogenetic trees were constructed by neighbor-joining (NJ) method. Significant bootstrap values are indicated as a percentage for 1000 replicates. Bootstrap values higher than 50 are displayed along the relative branches. Different genogroups in blocks are color coded. The numbers in the trees indicate the confidence and the bars under the trees indicate the phylogenetic distance.

Next, phylogenetic tree analysis using MEGA7.0 software was performed for the 21 Yunnan PoSaV strains and 60 selected PoSaV reference sequences representing 16 PoSaV genogroups (Supplementary Table S3). In both phylogenic trees constructed on either only *VP1* gene sequences (Figure 1A) or longer sequences (*VP1* and *VP2*) (Figure 1B), 20 identified Yunnan PoSaV strains (indicated by solid blue squares) were clustered together with the selected PoSaV-GIII reference strains (Figure 1, block highlighted in light green). On the other hand, one Yunnan PoSaV strain (YNQB, indicated by a red triangle) was clustered together with the 9 selected PoSaV-GV strains (Figure 1, highlighted in dark blue).

Finally, in agreement with the data from specific PCR amplification and sequence determination, phylogenetic tree analyses revealed that PoSaV infection is prevalent in Yunnan pig farms, with PoSaV-GIII to be the predominant genogroup. Furthermore, a PoSaV-GV strain was identified from Yunnan, which is the first report in China.

### High Genetic Diversity of Yunnan PoSaV-GIII Strains

**Figure 2:**
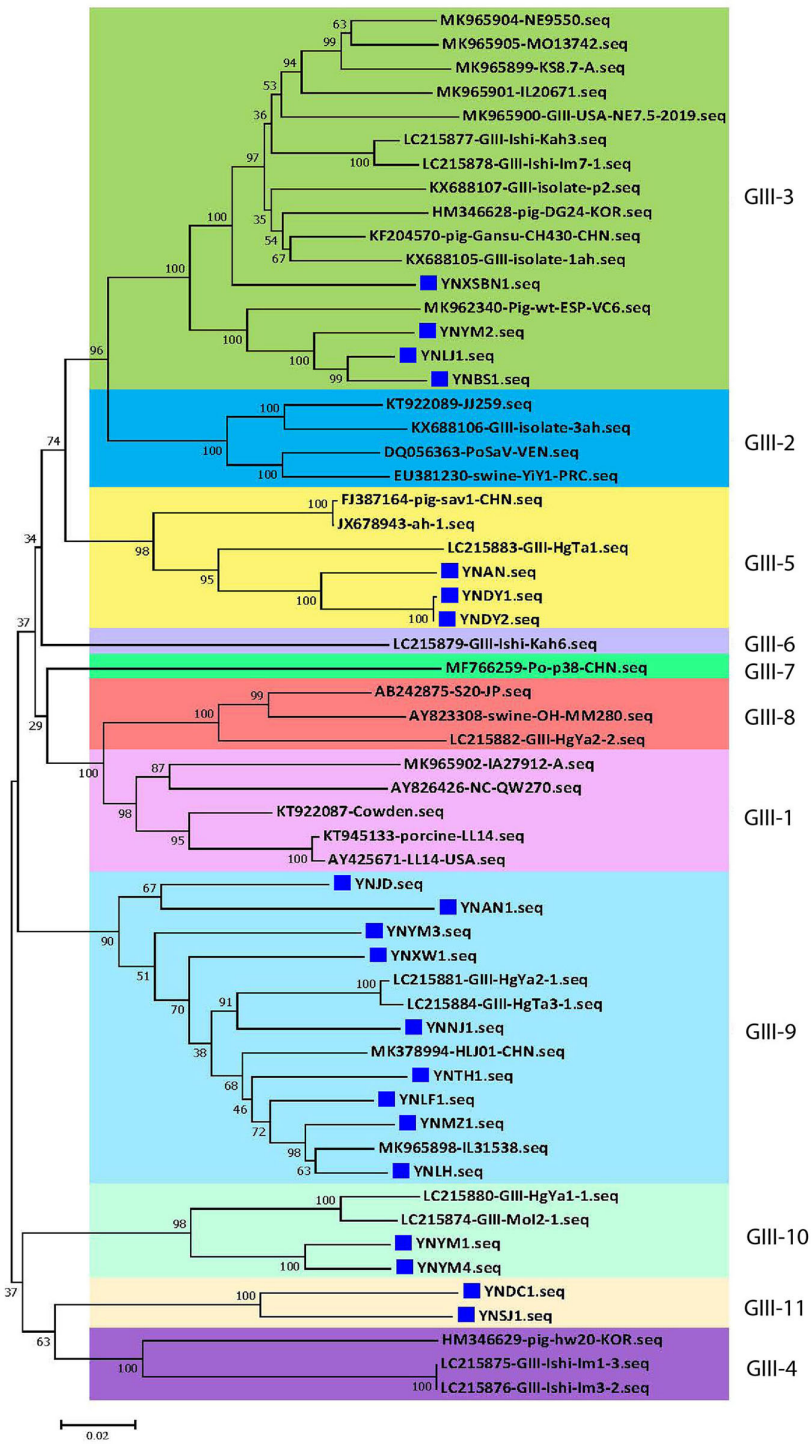
The 20 Yunnan PoSaV-GIII strains fell into 5 genotypes by phylogenetic analysis of the capsid genes (*VP1* and *VP2*), including 38 reference sequences retrieved from GenBank (Supplementary Table S3). The phylogenetic trees were constructed by neighbor-joining (NJ) method. Significant bootstrap values are indicated as a percentage for 1,000 replicates. Bootstrap values higher than 50 are displayed along the relative branches. Different genotypes are color coded. The blue squares represent the PoSaV sequences obtained in this study from Yunnan. The numbers in the trees indicate the confidence and the bars under the trees indicate the phylogenetic distance.

At present, the 19 genogroups of SaVs can be classified into at least 52 genotypes based on complete sequences of *VP1* genes using a pairwise distance cutoff value of ≤0.169 to distinguish different genotypes or clusters (1, 37). To further scrutinize the genetic diversity of the 20 Yunnan PoSaV-GIII strains, the complete sequences of *VP1* genes of these 20 strains were subjected to phylogenetic analysis together with 38 selected PoSaV-GIII reference sequences retrieved from NCBI (Supplementary Table S3). Phylogenetic analysis revealed that the 58 PoSaV-GIII *VP1* gene sequences could be divided into 11 genotypes (Figure 2), with the 20 Yunnan PoSaV-GIII strains dispersed into 5 genotypes (genotypes 3, 5, 9, 10, and 11) according to the previous classification (29). Sequence dissimilarity comparison (Supplementary Figure S1) confirmed that the sequence discrepancy within a cluster was ≤0.169 and that the sequence disparity between different clusters was mostly >0.169, in agreement with the widely accepted genotype definition (1, 37). Furthermore, when correlating the identification years and places of the PoSaV-GIII strains (Supplementary Table S3) with the genotype assignment (Figure 2), for most of the clusters, we did not find clear clustering profiles based on the years or places of strain identification. Besides, none of the 20 Yunnan PoSaV-GIII strains grouped together with the Cowden strain (KT922087 in genotype 1), and the 4 Yunnan PoSaV-GIII strains in genotype 3 grouped together with 12 PoSaV-GIII strains identified from USA, Spain, Korea, Japan, and China between 2012 and 2019. Taken together, the in-depth phylogenetic analysis revealed high genetic diversity of Yunnan PoSaV-GIII strains.

**Figure 3:**
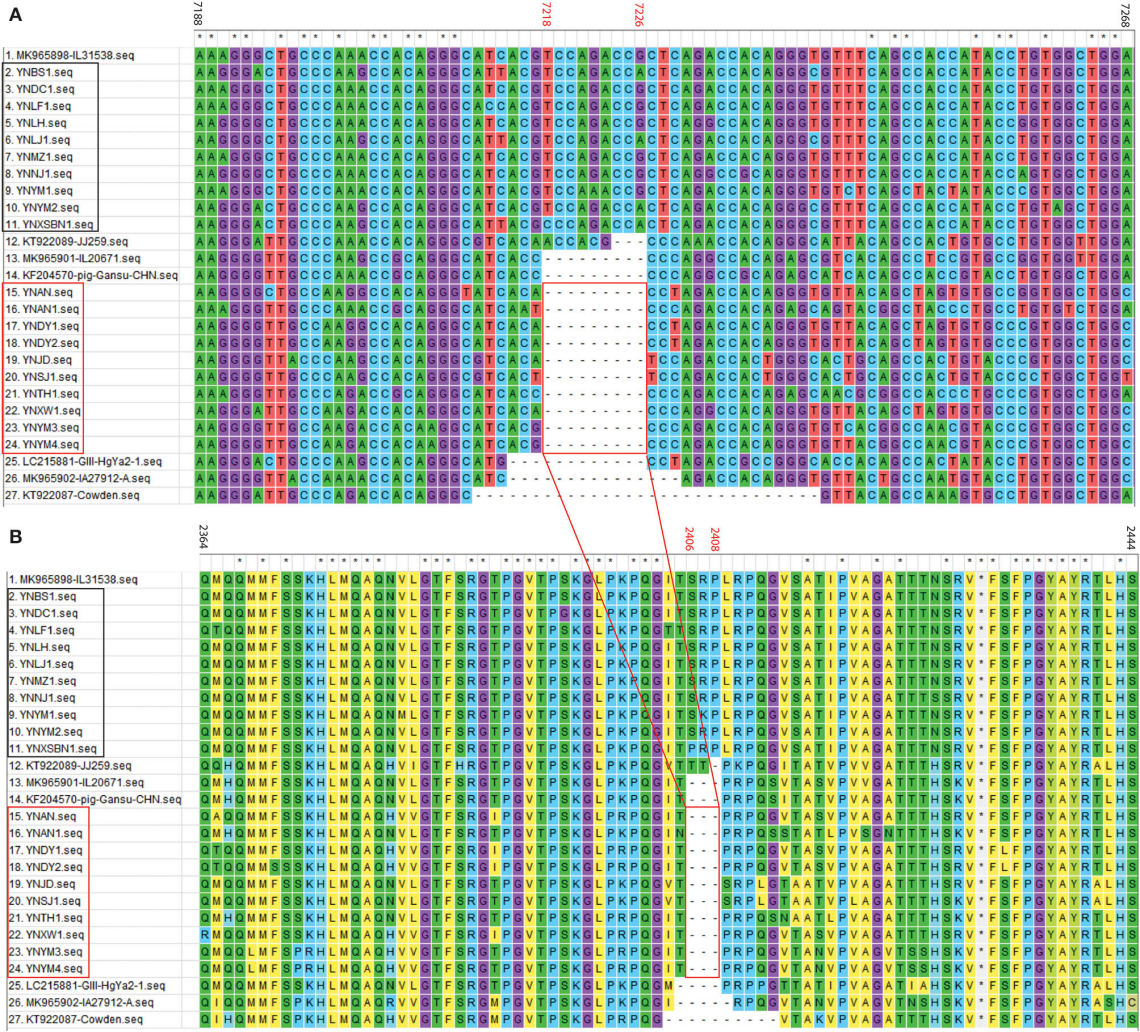
Nucleotide **(A)** and amino acid **(B)** sequence alignment of partial *VP2* region for PoSaV-GIII strains. ClustalW method with the default parameters was used to perform the similarity analysis of the *VP2* gene sequences between the 7 selected PoSaV-GIII *VP2* reference sequences (Supplementary Table S3) and the 20 Yunnan PoSaV-GIII *VP2* sequences obtained in this study (Supplementary Table S4 and Supplementary File). The positions of sequences were provided using MK985898 as reference. The 10 Yunnan PoSaV-GIII *VP2* sequences without deletions are indicated with the black square, whereas the other 10 Yunnan PoSaV-GIII *VP2* sequences with deletions are indicated with the red square. On top of the aligned sequences, numbers in black stands for the sequence region in comparison, whereas the numbers in red (7218–7226 in A for nucleic acid locations and 2406–2408 in B for the corresponding amino acid locations) indicate the deletion region, which is highlighted by the red squares and the connecting lines. * indicated identical nucleic acid or amino acid sequences across the porcine sapovirus strains.

For the 20 Yunnan PoSaV-GIII strains identified in this study, the sequence assembly revealed that the length of *VP1* gene is 1635 nt, whereas the length of *VP2* gene is 516 (10 strains with 9 nt deletions, highlighted in the red square in Figure 3A) or 525 nt (another 10 strains without deletions, highlighted in the black square in Figure 3A). In addition, the genome sequences were determined for 3 PoSaV-GIII strains with different lengths (7341 nt for YNJD and YNAN, whereas 7350 nt for YNLH). Analysis using MegAlign function incorporated in software DNASTAR 6.0 revealed that the nucleic acid sequence similarity between the 20 Yunnan PoSaV-GIII capsid protein genes (*VP1* and *VP2*) ranged from 75.3% (YNSJ1 vs. YNYM2) to 99.9% (YNDY1 vs. YNDY2) as highlighted in red squares (Supplementary Figure S1), on the other hand, when cross-comparing the 20 Yunnan PoSaV-GIII sequences with the 38 selected PoSaV-GIII reference sequences, as highlighted in black squares (Supplementary Figure S1), the sequence disparity of capsid protein genes (*VP1* and *VP2*) is between 3.5 (YNLH-MK965898) and 25.1% (YNSJ1 vs. MK965899, YNLH vs. LC215876, and YNLH vs. LC965875). Overall, high sequence discrepancy with deletions and insertions again implied high genetic diversity of Yunnan PoSaV-GIII strains.

Further sequence evaluation using MEGA 7.0 software revealed that 10 Yunnan PoSaV-GIII strains had a 9 nt deletion (7218–7226 nt) in the *VP2* region (Figure 3A, highlighted in red square), whereas the other 10 did not possess this deletion at the same location (Figure 3A, highlighted in black square), resulting in deletion of three amino acids (2406–2408 aa, Figure 3B), in agreement with a previous report (5) and further complicating the PoSaV genetic diversity. Additionally, when looking into the origin of the 20 Yunnan PoSaV-GIII strains (Table 1), we noticed that the PoSaV-GIII strains determined from the three biggest swine producing prefectures or cities (Chuxiong, Yuxi, and Kunming) in Yunnan province contained both types, with or without the 9 nt deletion in gene *VP2*: two strains from Chuxiong (YNDY1 and YNDY2), two strains from Kunming (YNAN and YNAN1) and three strains from Yuxi (YNTH1, YNYM3, and YNYM4) showed the deletion of 7218–7226 nt in *VP2* gene region, whereas one strain from Chuxiong (YNLF1), one strain from Kunming (YNDC1), and two strains from Yuxi (YNYM1 and YNYM2) showed no deletion of 7218–7226 nt. The data suggested that different sub-genotypes of PoSaV-GIII strains may be prevalent in some certain areas.

### Sequence Alignment and Analyses of the Yunnan PoSaV-GV Strain With Reference Sequences

**Figure 4:**
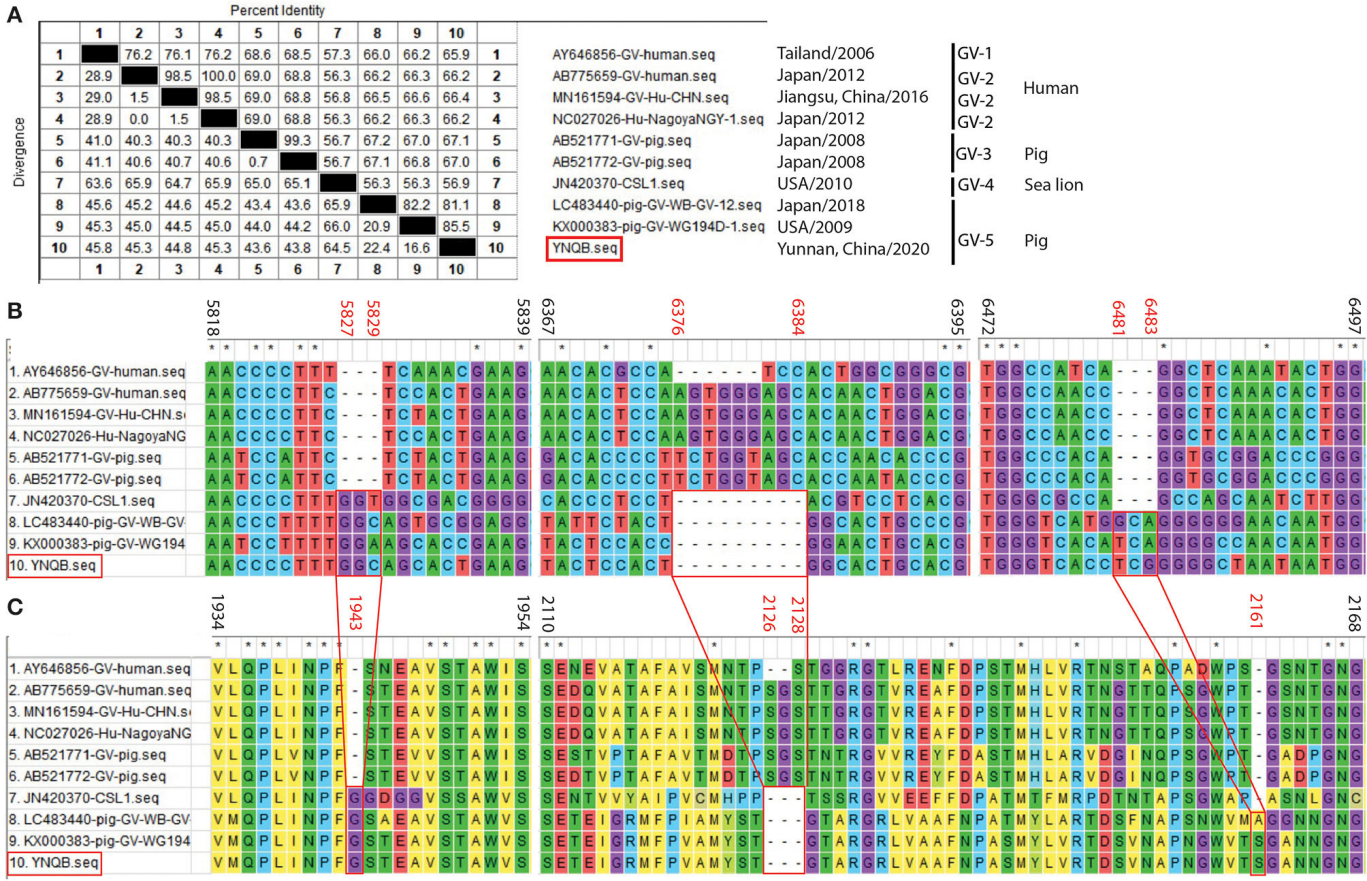
Sequence alignment and analyses of the Yunnan PoSaV-GV strain (YNQB, highlighted in red square) with reference sequences. Similarity of the sequenced genomes of YNQB and nine SaV-GV strains retrieved from NCBI **(A)**. Nucleotide **(B)** and amino acid **(C)** sequence alignment of partial *VP1* region of 10 SaV-GV strains. ClustalW method with the default parameters was used to perform the similarity analysis of *VP1* sequences of the nine selected SaV-GV reference sequences (Supplementary Table S3) and the Yunnan PoSaV-GV strain obtained in this study. The positions of sequences were provided using KX000383 as reference. On top of the aligned sequences, numbers in black stands for the sequence region in comparison, whereas the numbers in red (5827–5829, 6376–6384, and 6481–6483 in B for nucleic acid locations, whereas 1943, 2126–2128, and 2161 in C for the corresponding amino acid locations) indicate the indel regions, which is highlighted by the red squares and the connecting lines. * indicated identical nucleic acid or amino acid sequences across the porcine sapovirus strains.

One PoSaV-GV strain (YNQB) was identified from Yunnan in this study, which is the first report for PoSaV-GV strain in China. Sanger sequencing and sequence assembly revealed that the length of the sequenced genome, *VP1* gene, and *VP2* gene of YNQB is 7496, 1736, and 495 nt, respectively. Alignment of the sequenced genome of YNQB strain with all the 9 SaV-GV strains we extracted from NCBI revealed that the sequenced genome of the Yunnan PoSaV-GV strain (YNQB) from this study shared nucleic acid sequence similarity ranging from 56.9% (YNQB vs. JN420370 of sea lion origin) to 85.5% (YNQB vs. KX000383 of swine origin) (Figure 4A). According to the previous classification (47), SaV-GV strains could be classified into five genotypes (from GV-1 to GV-5) and the YNQB strain fell into cluster 5 together with another two SaV-GV strains of swine origin (Figure 4A).

Meanwhile, in the *VP1* gene region of the YNQB strain, we observed 3 nt insertion (position 5827–5829 nt), 9 nt deletion (position 6376–6384 nt), and 3 nt insertion (position 6481–6483 nt) (Figure 4B), resulting in a corresponding insertion of one amino acid (position 1943 aa), deletion of three amino acids (position 2126–2128 aa) and insertion of one amino acid (position 2161 aa) (Figure 4C), which are also presented in two out of the four SaV-GV strains of swine origin (LC483440 and KX000383). Of note, as shown in Figure 4A, two PoSaV-GV strains (AB521771 and AB521772, both from Japan in 2008) exhibit similar nucleic acid sequence profile (68.5–69.0% sequence similarity) with SaV-GV strains of human origin (AB775659, AY645856, MN161594, and NC027026), but shares lower sequence similarity (56.7–67.2%) with the other PoSaV-GV strains (LC4822440, KX000383, and YNQB).

### Recombinant Analyses of Yunnan PoSaV-GIII Strains

**Figure 5:**
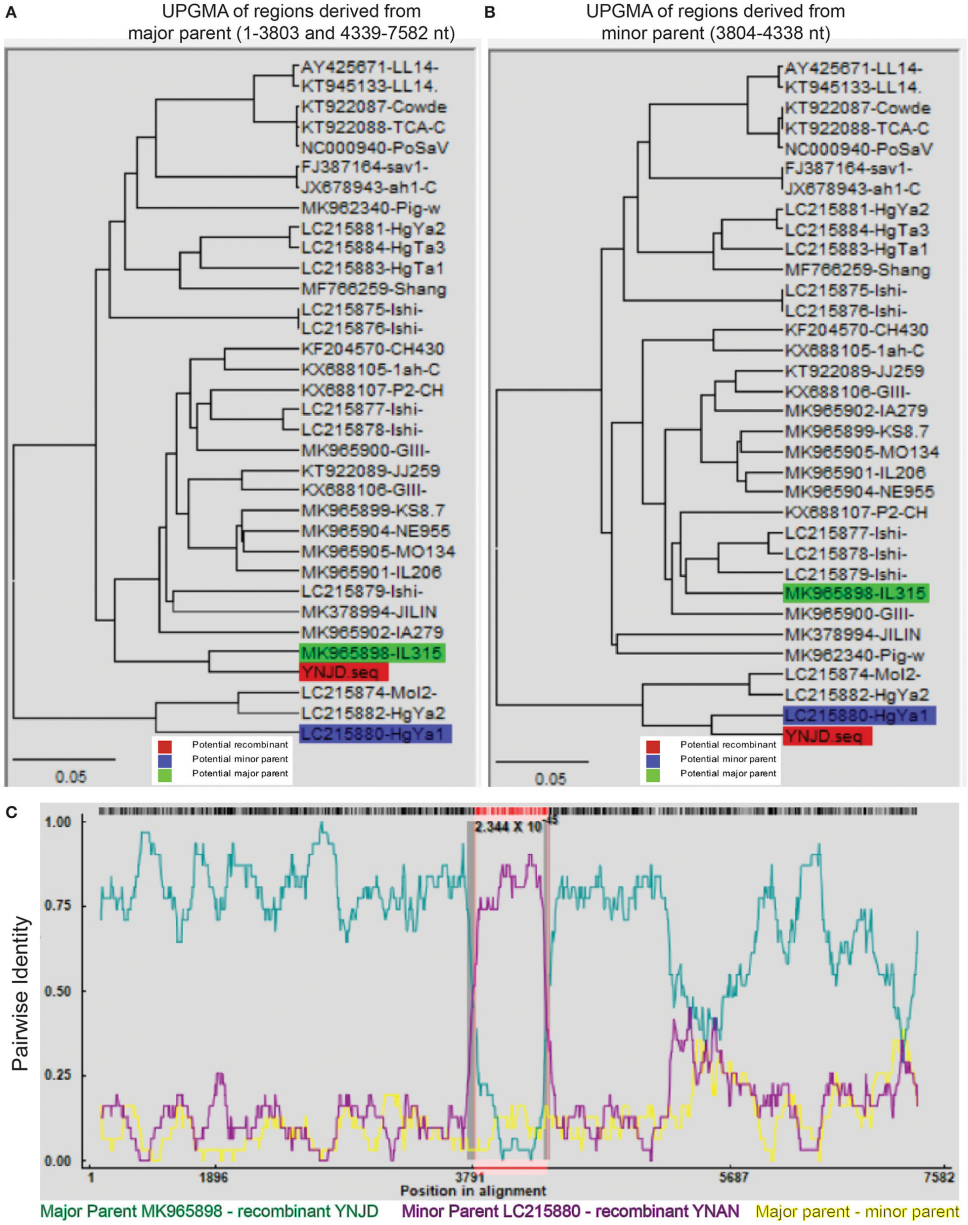
Recombination analysis of Yunnan PoSaV-GIII strain YNJD using RDP v.4 software. Phylogenetic tree was constructed based on the complete SaV genomic sequences using the unweighted pair-group method (UPGMA) (7) to illustrate the evolutionary relationship between the recombinant strain YNJD (highlighted in red block) and a major parent strain with similar sequence to MK965898 (highlighted in green block) in the region of 1–3803 nt and 4339–7582 nt **(A)**, and between the recombinant strain YNJD (highlighted in red block) and a minor parent strain with similar sequence to LC215880 (highlighted in blue block) in the region of 3804–4338 nt **(B)**. **(C)**. Analysis of the complete genomic sequences of MK965898 (green line) and LC215880 (pink line), with YNJD as the query sequence. Red, green, and blue color shades are used to label the recombinant, potential major and minor parent strains on each tree, respectively. In the RDP plots, turquoise blue lines are potential major parent-recombinant; purple lines are potential minor parent-recombinant; yellow lines are potential major parent-potential minor parent. The value below the red barcode indicates the confidence of the RDP analysis, with the smaller value standing for higher probability.

Recombination, both inter- and intra-genogroup, is common for SaVs (1, 5, 22, 48). To the best of our knowledge, there was only one report from China about intra-genogroup (PoSaV-GIII) recombination, where KT922089 and KF204570 were determined as the two parental strains (29) for KX688107 identified from Shanghai in 2015. To perform the recombination analyses for the four Yunnan PoSaV strains (YNLH, YNAN, YNJD, and YNQB), whose genome sequences were determined, using software RDP v.4 (44), analyses with stringent threshold (high confidence for all the computation programs) revealed that two PoSaV-GIII strains out of the four genomes (three PoSaV-GIII strains and one PoSaV-GV strain) may be resulted from multiple recombination events. As shown in Figure 5, YNJD strain may come from the recombination between strains with similar sequences to major parent MK965898 (regions of 1–3803 and 4339–7582 nt) and minor parent LC215880 (region of 3804–4338 nt) with high confidence 2.344 × 10^−45^. Meanwhile, as shown in Supplementary Figure S2, YNAN strain may originate from the recombination between strains with similar sequences to major parent MK965898 (regions of 1–5195 and 6352–7583 nt) and minor parent FJ387164 (region of 5196–6351 nt) with high confidence 1.303 × 10^−35^. In agreement with most of other SaV recombination events, the breakpoints for Yunnan PoSaV-GIII strain YNJD and previously reported Shanghai PoSaV-GIII strain KX688107 (29) located in the RdRp-capsid junction region, which is critical for virus replication. However, we noticed that the breakpoint for Yunnan PoSaV-GIII strain YNAN located in the VP1 region, which is important for immunogenicity. In summary, the distinct recombination background with different parental strains and different breakpoint regions for the three recombinants from China further illustrated the high genetic diversity of Yunnan PoSaV-GIII strains.

## Discussion

Comparing with the other provinces in China, Yunnan is one of the most important swine-producing provinces in China (42). Long geographic borders with Vietnam, Laos, and Myanmar, and extensive trade with foreign countries potentially exacerbated the high risk of disease transmission in pig herds. Porcine SaV is widely detected throughout the world, and the first outbreak of gastroenteritis in piglets caused by PoSaV in China was reported from Shanghai in 2008 (32); however, the relative information on the genetic characterization of PoSaV in China is still rather limited, with no information from Yunnan province. Therefore, it is imperative to investigate the genetic diversity and relationship of PoSaV strains currently circulating in Yunnan. Between January and May 2020, many pig farms in Yunnan had experienced months of enteric diseases, especially with severe diarrhea in piglets. Per the veterinarians' request, 202 fecal samples were collected from 3 to 50-day-old diarrheic piglets and sent to College of Animal Veterinary Medicine, Yunnan Agricultural University for the identification of the causative pathogens of disease.

The RT-PCR detection revealed that the PoSaV infection rate in Yunnan is 35.2% (Table 1, 71/202), much higher than in other areas in China. For example, the PoSaV-positive rates of fecal samples from diarrheic piglets by RT-PCR for Hunan province (samples collected between August 2006 and July 2007), Guangdong province (samples collected between November 2011 and April 2013), and Xinjiang province (samples collected between January 2013 and December 2014) were 14.73 (22/153) (33), 6.9 (7/101) (36), and 3.42% (5/146) (28), respectively. Considering that similar prevalence of PoSaVs was detected in diarrheic and non-diarrheic pigs (37), we speculate the overall PoSaV infection rate in different populations of pigs in Yunnan could be in the range 30 and 40%, which is high enough to draw sufficient attention from the veterinarians and the governments.

A total of twenty out of the 21 PoSaV strains obtained in this study belonged to GIII (Figure 1) and shared 75.3–100% sequence identities in the capsid protein genes *VP 1* and *VP2* (Supplementary Figure S1), which was more divergent than a recent study (22). The 20 GIII strains were rather different and did not completely cluster together. Instead, they formed five genotypes (Figure 2), suggesting that multiple strains co-circulate in Yunnan pig populations. In addition, a PoSaV-GV strain (YNQB) was identified in this study. To the best of our knowledge, this is the first PoSaV-GV case reported from piglets in China. Of note, genogroup GIII was also identified from the pig farm where the GV strain was determined, but from different piglets, indicating the simultaneous presence of two genogroups of PoSaV in a single pig farm. Despite the presence of two genogroups of PoSaVs (GIII and GV), GIII was the predominant genogroup in our sample set, in consent with recent reports (5, 22).

A total of two genogroups (PoSaV-GIII and PoSaV-GV) and diverse PoSaV-GIII strains are co-circulating in Yunnan, providing adequate niches for generating new recombinant strains through intra- and inter-genogroup recombination. Recombination analyses revealed that two PoSaV-GIII strains (YNJD and YNAN) may have evolved through intra-genogroup recombination events (Figure 5 and Supplementary Figure S2), where a strain with similar sequence to MK965898 is the major parent for both recombination events. With optimal amplification conditions and ideal virus isolation, more whole genome sequences can be determined for Yunnan PoSaV-GIII strains, and very likely, more recombinants may be discovered, which will further complicate the control and prevention of PoSaV infection.

In addition to recombination, SaVs also exploit a genetic drift mechanism (insertions, deletions, and mutations) to maximize viral fitness (5, 39). A total of ten out of the twenty PoSaV-GIII strains determined in this study have an identical 9 nt deletion in the VP2 gene region as the Cowden strain, resulting in a deletion of 3 aa (Figure 3), when using MK965898 as the reference sequence. We also identified a variable region in the 3′ end of *VP1* in the Yunnan PoSaV-GV strain YNQB. Compared with the reference strain AB775659, YNQB has a 9-nt deletion and two 3-nt-long insertions, which correspond to a deletion of 3 amino acids and two insertions of one amino acid (Figure 4). Both *VP1* and *VP2* are viral capsid proteins, which are closely related to immunogenicity and essential for the production of infectious virions (49); therefore, we speculate that these indels in the *VP1* and *VP2* genes may lead to immune escape and even change of the virulence. Isolation of the PoSaV strains and reverse genetic tools are warranted for future studies to explore the role of indels in virus replication and pathogenicity.

In summary, our study reported the detection and genetic characterization of PoSaVs in diarrheic piglets of 3–50 days from different prefectures or cities in Yunnan province during the period of January and May in 2020. The overall infection rate of PoSaV in Yunnan was 35.2%, much higher than in other areas in China. A total of two genogroups (GIII and GV) of PoSaVs were detected, with GIII strains of high genetic divergency predominating in Yunnan pigs. A total of two Yunnan PoSaV-GIII strains (YNJD and YNAN) may have evolved through intra-genogroup recombination events. Our findings provided significant insights into the epidemiology PoSaV in Yunnan and reported the first identification of PoSaV-GV in China, which is critical for the future vaccine development; however, continued surveillances on PoSaVs are indispensable to monitor viral evolution in pigs.

## Conclusion

In conclusion, we reported here the first molecular epidemiological investigation of porcine sapovirus (PoSaV) infection in Yunnan, China. Then, the overall infection rate of PoSaV in Yunnan was 35.2%, much higher than in other areas in China from previous studies. In addition, twenty PoSaV strains were partially or completely sequencing, where for the first time, a PoSaV GV strain was identified from China. Furthermore, two out of the twenty PoSaV-GIII strains were revealed to be recombinants. Eventually, our comprehensive analyses illustrated high genetic diversity of the Yunnan PoSaV.

## Data Availability Statement

The datasets presented in this study can be found in online repositories. The names of the repository/repositories and accession number(s) can be found in the article/Supplementary Material.

## Author Contributions

XL and CS: investigation, methodology, and experiments. YL, KQ, JunyB, and JunlB: methodology and data curation. YW and YY: methodology and experiments. JS: investigation, data curation, and writing. ZG, GL, and JL: writing, reviewing, and editing. CS and GY: conceptualization, supervision, and funding acquisition. All authors contributed to the article and approved the submitted version.

## Funding

This work was supported by the Major Specialized Projects of Yunnan Science and Technology Establishments and applications of prevention and control technology system for important pig diseases in Yunnan Province (202102AE090007), by Yunnan Technological Innovation Talents Program (202105AD160036), and by Program for Innovative Research Team (in Science and Technology) in University of Yunnan Province (IRTSTYN). The funders had no role in study design, data collection and interpretation, or the decision to submit the work for publication.

## Conflict of Interest

The authors declare that the research was conducted in the absence of any commercial or financial relationships that could be construed as a potential conflict of interest.

## Publisher's Note

All claims expressed in this article are solely those of the authors and do not necessarily represent those of their affiliated organizations, or those of the publisher, the editors and the reviewers. Any product that may be evaluated in this article, or claim that may be made by its manufacturer, is not guaranteed or endorsed by the publisher.

## Supplementary Material

Supplementary Figure S1 Nucleic acid sequence comparison of capsid protein genes (*VP1* and *VP2*) between the 38 selected PoSaV-GIII reference sequences (Supplementary Table S3) and the 20 Yunnan PoSaV-GIII strains (indicated with solid blue squares, Supplementary Table S4 and Supplementary File) by using DNAStar 6.0 software with the default parameters. Cluster numbers highlighted in red correspond to the phylogenetic analysis shown in Figure 2. The numbers in the sequence comparison table present the sequence dissimilarity. The numbers in hollow red squares show the lowest and highest sequence disparity between the 20 Yunnan PoSaV-GIII strains, whereas the numbers in hollow black squares display the lowest and highest sequence discrepancy between the 20 Yunnan PoSaV-GIII strains and the 38 selected PoSaV-GIII reference sequences.

Supplementary Figure S2 Recombination analysis of Yunnan PoSaV-GIII strain YNAN using RDP v.4 software. Phylogenetic tree was constructed based on the complete SaV genomic sequences using the unweighted pair-group method (UPGMA) (45) to illustrate the evolutionary relationship between the recombinant strain YNAN (highlighted in red block) and a major parent strain with similar sequence to MK965898 (highlighted in green block) in the region of 1–5195 and 6352–7583 nt **(A)**, and between the recombinant strain YNAN (highlighted in red block) and a minor parent strain with similar sequence to FJ387164 (highlighted in blue block) in the region of 5196–6351 nt **(B)**. **(C)** Analysis of the complete genomic sequences of MK965898 (green line) and FJ387164 (pink line), with YNAN as the query sequence. Red, green, and blue color shades are used to label the recombinant, potential major, and potential minor parent strains on each tree, respectively. In the RDP plots, turquoise blue lines are potential major parent-recombinant; purple lines are potential minor parent-recombinant; yellow lines are potential major parent-potential minor parent. The value below the red barcode indicates the confidence of the RDP analysis, with the smaller value standing for higher probability.

## References

[1] Alcala AC Rodriguez-Diaz J de Rolo M Vizzi E Buesa J Liprandi F. Seroepidemiology of porcine enteric sapovirus in pig farms in Venezuela. Vet Immunol Immunopathol. (2010) 137:269–74. 10.1016/j.vetimm.2010.06.005 20621364

[2] Becker-Dreps S Bucardo F Vinje J. Sapovirus: an important cause of acute gastroenteritis in children. Lancet Child Adolesc Health. (2019) 3:758–9. 10.1016/S2352-4642(19)30270-6 31439497 PMC7213765

[3] Becker-Dreps S Gonzalez F Bucardo F. Sapovirus: an emerging cause of childhood diarrhea. Curr Opin Infect Dis. (2020) 33:388–97. 10.1097/QCO.0000000000000671 32796163 PMC7748384

[4] Chiba S Sakuma Y Kogasaka R Akihara M Horino K Nakao T. An outbreak of gastroenteritis associated with calicivirus in an infant home. J Med Virol. (1979) 4:249–54. 10.1002/jmv.1890040402 232140

[5] Dufkova L Scigalkova I Moutelikova R Malenovska H Prodelalova J. Genetic diversity of porcine sapoviruses, kobuviruses, and astroviruses in asymptomatic pigs: an emerging new sapovirus GIII genotype. Arch Virol. (2013) 158:549–58. 10.1007/s00705-012-1528-z 23124843

[6] Flewett TH Davies H. Letter: Caliciviruses in man. Lancet. (1976) 1:311. 10.1016/S0140-6736(76)91450-1 55627

[7] Hall BG Barlow M. Phylogenetic analysis as a tool in molecular epidemiology of infectious diseases. Ann Epidemiol. (2006) 16:157–69. 10.1016/j.annepidem.2005.04.010 16099674

[8] Hansman GS Natori K Oka T Ogawa S Tanaka K Nagata N. Cross-reactivity among sapovirus recombinant capsid proteins. Arch Virol. (2005) 150:21–36. 10.1007/s00705-004-0406-8 15449145

[9] Hansman GS Oka T Sakon N Takeda N. Antigenic diversity of human sapoviruses. Emerg Infect Dis. (2007) 13:1519–25. 10.3201/eid1310.070402 18258001 PMC2851512

[10] Jun Q Lulu T Qingling M Xingxing Z Haiting L Shasha G. Serological and molecular investigation of porcine sapovirus infection in piglets in Xinjiang, China. Trop Anim Health Prod. (2016) 48:863–9. 10.1007/s11250-016-1023-8 26898687

[11] Katsuta R Sunaga F Oi T Doan YH Tsuzuku S Suzuki Y. First identification of Sapoviruses in wild boar. Virus Res. (2019) 271:197680. 10.1016/j.virusres.2019.197680 31398366

[12] Kumar S Stecher G Tamura K. MEGA7: Molecular Evolutionary Genetics Analysis Version 7.0 for Bigger Datasets. Mol Biol Evol. (2016) 33:1870–4. 10.1093/molbev/msw054 27004904 PMC8210823

[13] Kuroda M Masuda T Ito M Naoi Y Doan YH Haga K. Genetic diversity and intergenogroup recombination events of sapoviruses detected from feces of pigs in Japan. Infect Genet Evol. (2017) 55:209–17. 10.1016/j.meegid.2017.09.013 28923281

[14] Lauritsen KT Hansen MS Johnsen CK Jungersen G Bottiger B. Repeated examination of natural sapovirus infections in pig litters raised under experimental conditions. Acta Vet Scand. (2015) 57:60. 10.1186/s13028-015-0146-7 26410386 PMC4583762

[15] Li J Shen Q Zhang W Zhao T Li Y Jiang J. Genomic organization and recombination analysis of a porcine sapovirus identified from a piglet with diarrhea in China. Virol J. (2017) 14:57. 10.1186/s12985-017-0729-1 28302145 PMC5356244

[16] Li JY Mai W Tan HQ Jian MT Deng H Chen ZP. An outbreak of acute gastroenteritis caused by Sapovirus in a community of Guangdong province. Zhonghua Liu Xing Bing Xue Za Zhi. (2020) 41:226–30. 10.3760/cma.j.issn.0254-6450.2020.02.016 32164134

[17] Liu GH Li RC Huang ZB Yang J Xiao CT Li J. RT-PCR test for detecting porcine sapovirus in weanling piglets in Hunan Province, China. Trop Anim Health Prod. (2012) 44:1335–9. 10.1007/s11250-012-0138-9 22492394

[18] Ueki Y Shoji M Okimura Y Miyota Y Masago Y Oka T. Detection of Sapovirus in oysters. Microbiol Immunol. (2010) 54:483–6. 10.1111/j.1348-0421.2010.00239.x 20646214

[19] Liu GH Li RC Li J Huang ZB Xiao CT Luo W. Seroprevalence of porcine cytomegalovirus and sapovirus infection in pigs in Hunan province, China. Arch Virol. (2012) 157:521–4. 10.1007/s00705-011-1189-3 22167251

[20] Liu W Yang B Wang E Liu J Lan X. Complete sequence and phylogenetic analysis of a porcine sapovirus strain isolated from western China. Virus Genes. (2014) 49:100–5. 10.1007/s11262-014-1078-4 24792514

[21] Liu ZK Li JY Pan H. Seroprevalence and molecular detection of porcine sapovirus in symptomatic suckling piglets in Guangdong Province, China. Trop Anim Health Prod. (2014) 46:583–7. 10.1007/s11250-013-0531-z 24407531

[22] Madeley CR Cosgrove BP. Letter: Caliciviruses in man. Lancet. (1976) 1:199–200. 10.1016/S0140-6736(76)91309-X 54714

[23] Martin D. P. Murrell B. Golden M. Khoosal A. Muhire B. (2015). RDP4: Detection and analysis of recombination patterns in virus genomes. Virus Evol 1, vev003. 10.1093/ve/vev003 27774277 PMC5014473

[24] Mauroy A Van der Poel WH der Honing RH Thys C Thiry E. Development and application of a SYBR green RT-PCR for first line screening and quantification of porcine sapovirus infection. BMC Vet Res. (2012) 8:193. 10.1186/1746-6148-8-193 23072668 PMC3528410

[25] Melegari I Marsilio F Profio Di Sarchese F Massirio V Palombieri I. Seroprevalence of sapovirus in dogs using baculovirus-expressed virus-like particles. Virus Res. (2018) 251:1–5. 10.1016/j.virusres.2018.04.014 29698676

[26] Mombo IM Berthet N Bouchier C Fair JN Schneider BS Renaud F. Characterization of a genogroup I sapovirus isolated from chimpanzees in the republic of congo. Genome Announc. (2014) 2. 10.1128/genomeA.00680-14 25035327 PMC4102864

[27] Nagai M Wang Q Oka T Saif LJ. Porcine sapoviruses: Pathogenesis, epidemiology, genetic diversity, and diagnosis. Virus Res. (2020) 286:198025. 10.1016/j.virusres.2020.198025 32470356 PMC7255249

[28] Nakamura K Saga Y Iwai M Obara M Horimoto E Hasegawa S. Frequent detection of noroviruses and sapoviruses in swine and high genetic diversity of porcine sapovirus in Japan during Fiscal Year 2008. J Clin Microbiol. (2010) 48:1215–22. 10.1128/JCM.02130-09 20164276 PMC2849587

[29] Oka T Katayama K Ogawa S Hansman GS Kageyama T Ushijima H. Proteolytic processing of sapovirus ORF1 polyprotein. J Virol. (2005) 79:7283–90. 10.1128/JVI.79.12.7283-7290.2005 15919882 PMC1143638

[30] Oka T Lu Z Phan T Delwart EL Saif LJ Wang Q. Genetic characterization and classification of human and animal sapoviruses. PLoS ONE. (2016) 11:e0156373. 10.1371/journal.pone.0156373 27228126 PMC4881899

[31] Oka T Mori K Iritani N Harada S Ueki Y Iizuka S. Human sapovirus classification based on complete capsid nucleotide sequences. Arch Virol. (2012) 157:349–52. 10.1007/s00705-011-1161-2 22075918

[32] Oka T Wang Q Katayama K Saif LJ. Comprehensive review of human sapoviruses. Clin Microbiol Rev. (2015) 28:32–53. 10.1128/CMR.00011-14 25567221 PMC4284302

[33] Wang L Marthaler D Fredrickson R Gauger PC Zhang J Burrough ER. Genetically divergent porcine sapovirus identified in pigs, United States. Transbound Emerg Dis. (2020) 67:18–28. 10.1111/tbed.13337 31461567

[34] Wang QH Han MG Funk JA Bowman G Janies DA Saif LJ. Genetic diversity and recombination of porcine sapoviruses. J Clin Microbiol. (2005) 43:5963–72. 10.1128/JCM.43.12.5963-5972.2005 16333083 PMC1317165

[35] Yang S Zhang W Shen Q Huang F Wang Y Zhu J. Molecular characterization and phylogenetic analysis of the complete genome of a porcine sapovirus from Chinese swine. Virol J. (2009) 6:216. 10.1186/1743-422X-6-216 19961620 PMC2795755

[36] Yinda CK Conceicao-Neto N Zeller M Heylen E Maes P Ghogomu SM. Novel highly divergent sapoviruses detected by metagenomics analysis in straw-colored fruit bats in Cameroon. Emerg Microbes Infect. (2017) 6:e38. 10.1038/emi.2017.20 28536431 PMC5520483

[37] Okitsu S Khamrin P Thongprachum A Hikita T Kumthip K Pham NTK. Diversity of human sapovirus genotypes detected in Japanese pediatric patients with acute gastroenteritis, 2014-2017. J Med Virol. (2021) 93:4865–74. 10.1002/jmv.26934 33704833

[38] Rahman R Rahman S Afrad MMH Talha M Islam D Uddin KMM. Epidemiology and genetic characterization of human sapovirus among hospitalized acute diarrhea patients in Bangladesh, 2012-2015. J Med Virol. (2021). 10.1002/jmv.27125 34081341

[39] Ren K Wang R Liu X Liu Y Zhang J Bi J. Epidemiological investigation and genetic characterization of porcine astrovirus genotypes 2 and 5 in Yunnan province, China. Arch Virol. (2021). 10.1007/s00705-021-05311-8 34839421 PMC8627673

[40] Reuter G Zimsek-Mijovski J Poljsak-Prijatelj M Di Bartolo I Ruggeri FM Kantala T. Incidence, diversity, and molecular epidemiology of sapoviruses in swine across Europe. J Clin Microbiol. (2010) 48:363–368. 10.1128/JCM.01279-09 19940055 PMC2815582

[41] Zhang W Shen Q Hua X Cui L Liu J Yang S. The first Chinese porcine sapovirus strain that contributed to an outbreak of gastroenteritis in piglets. J Virol. (2008) 82:8239–40. 10.1128/JVI.01020-08 18508889 PMC2519573

[42] Sachsenroder J Braun A Machnowska P Ng TFF Deng X Guenther S. Metagenomic identification of novel enteric viruses in urban wild rats and genome characterization of a group A rotavirus. J Gen Virol. (2014) 95:2734–47. 10.1099/vir.0.070029-0 25121550 PMC4811644

[43] Saif LJ Bohl EH Theil KW Cross RF House JA. Rotavirus-like, calicivirus-like, and 23-nm virus-like particles associated with diarrhea in young pigs. J Clin Microbiol. (1980) 12:105–11. 10.1128/jcm.12.1.105-111.1980 6252238 PMC273530

[44] Shen H Zhang J Gauger PC Burrough ER Harmon K Wang L. Genetic characterization of porcine sapoviruses identified from pigs during a diarrhoea outbreak in Iowa, 2019. Transbound Emerg Dis. (2021) 69:1246–55. 10.1111/tbed.14087 33780163

[45] Valente CS Alfieri AF Barry AF Leme RA Lorenzetti E Alfieri AA. Age distribution of porcine sapovirus asymptomatic infection and molecular evidence of genogroups GIII and GIX? circulation in distinct Brazilian pig production systems. Trop Anim Health Prod. (2016) 48:21–7. 10.1007/s11250-015-0912-6 26385461

[46] Soma T Nakagomi O Nakagomi T Mochizuki M. Detection of Norovirus and Sapovirus from diarrheic dogs and cats in Japan. Microbiol Immunol. (2015) 59:123–8. 10.1111/1348-0421.12223 25545754 PMC7168372

[47] Song YJ Yu JN Nam HM Bak HR Lee JB Park SY. Identification of genetic diversity of porcine Norovirus and Sapovirus in Korea. Virus Genes. (2011) 42:394–401. 10.1007/s11262-011-0588-6 21369826

[48] Sosnovtsev SV Belliot G Chang KO Onwudiwe O Green KY. Feline calicivirus VP2 is essential for the production of infectious virions. J Virol. (2005) 79:4012–24. 10.1128/JVI.79.7.4012-4024.2005 15767403 PMC1061574

[49] Stamelou E Giantsis IA Papageorgiou KV Petridou E Davidson I Polizopomicronulou ZS. Epidemiology of Astrovirus, Norovirus and Sapovirus in Greek pig farms indicates high prevalence of Mamastrovirus suggesting the potential need for systematic surveillance. Porcine Health Manag. (2022) 8:5. 10.1186/s40813-021-00245-8 35000615 PMC8744241

