# Supplementary figures and images for: High Genetic Diversity of Porcine Sapovirus From Diarrheic Piglets in Yunnan Province, China
Source: Front Vet Sci. 2022 Jul 7;9:854905. doi: 10.3389/fvets.2022.854905 (PMC9300989; doi:10.3389/fvets.2022.854905)

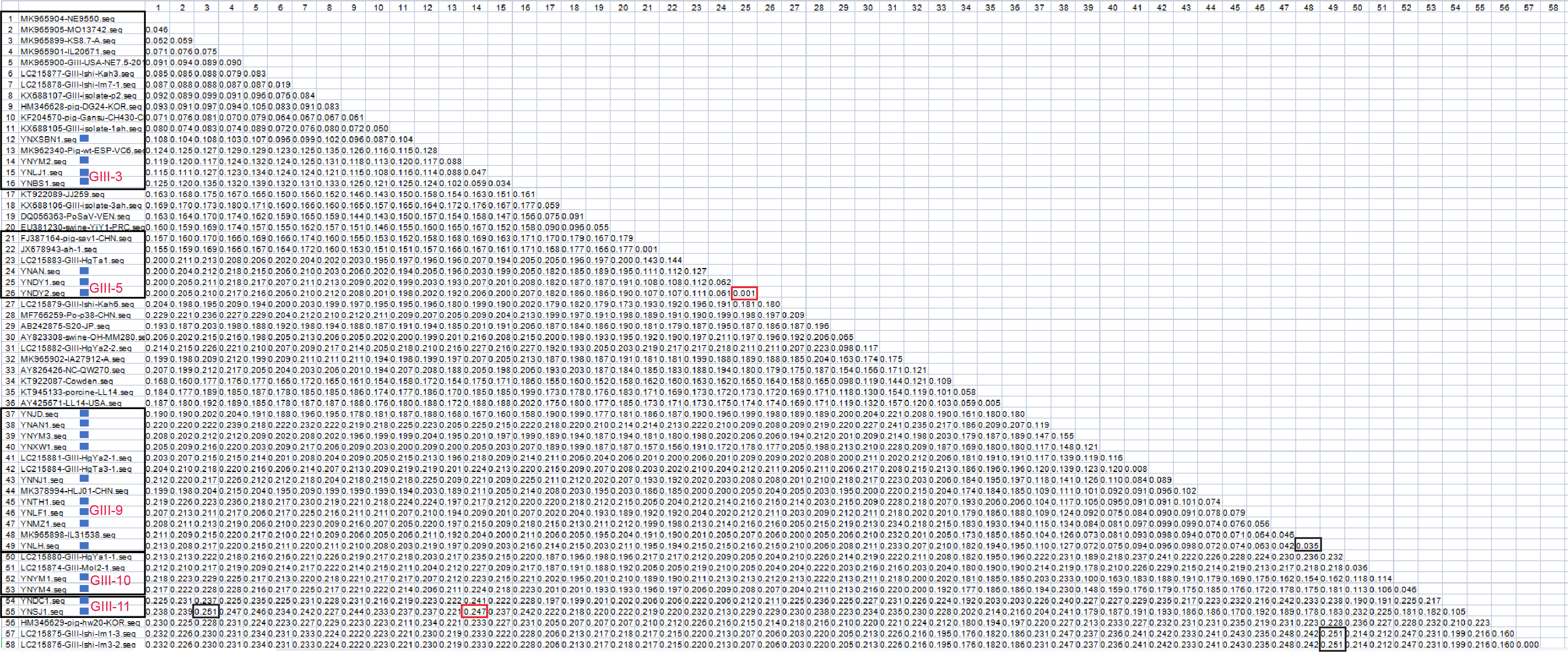

Supplement: Supplementary Figure S1 — Nucleic acid sequence comparison of capsid protein genes (VP1 and VP2) between the 38 selected PoSaV-GIII reference sequences (Supplementary Table S3) and the 20 Yunnan PoSaV-GIII strains (indicated with solid blue squares, Supplementary Table S4 and Supplementary File) by using DNAStar 6.0 software with the default parameters. Cluster numbers highlighted in red correspond to the phylogenetic analysis shown in Figure 2. The numbers in the sequence comparison table present the sequence dissimilarity. The numbers in hollow red squares show the lowest and highest sequence disparity between the 20 Yunnan PoSaV-GIII strains, whereas the numbers in hollow black squares display the lowest and highest sequence discrepancy between the 20 Yunnan PoSaV-GIII strains and the 38 selected PoSaV-GIII reference sequences. [file Image_1.JPEG]

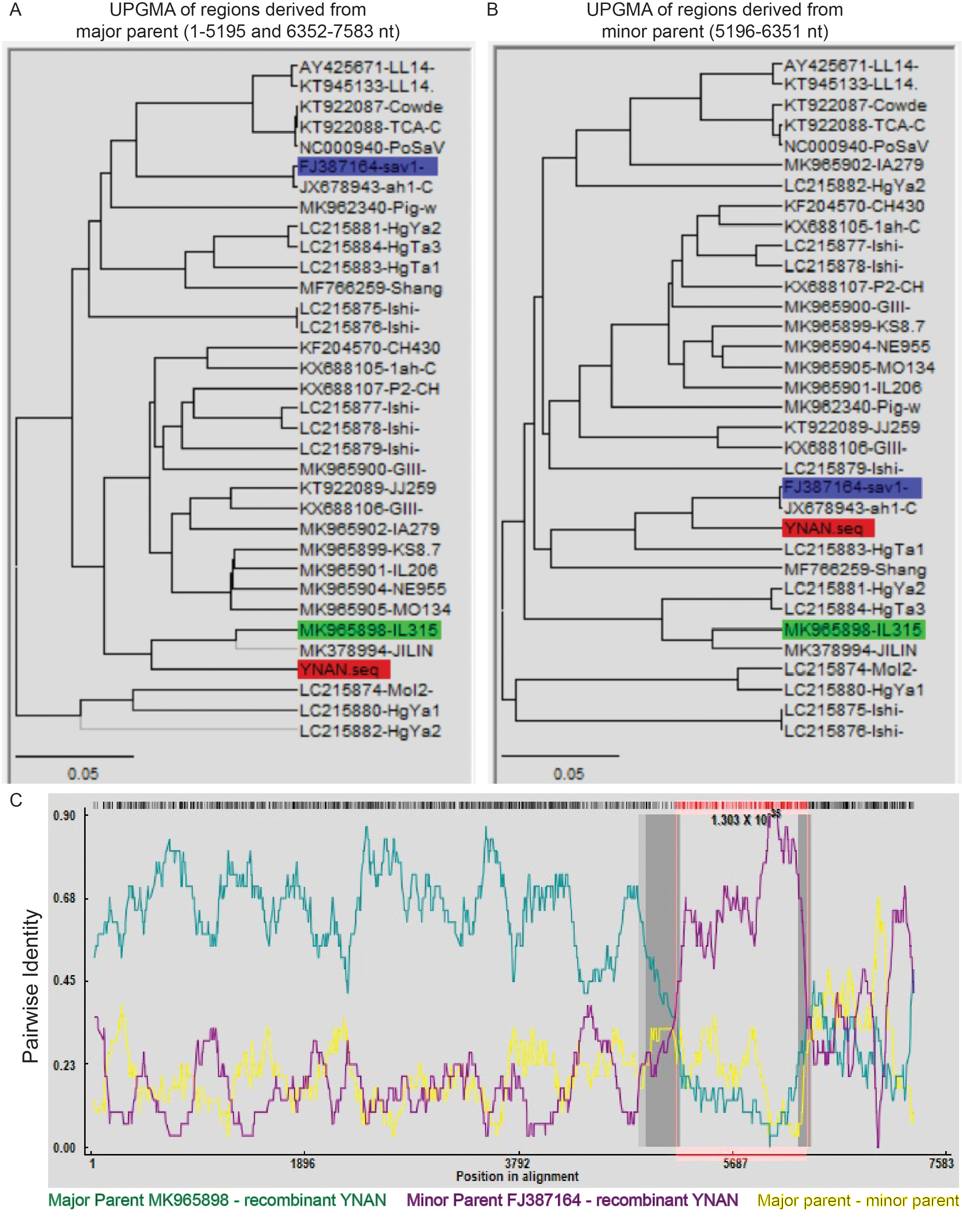

Supplement: Supplementary Figure S2 — Recombination analysis of Yunnan PoSaV-GIII strain YNAN using RDP v.4 software. Phylogenetic tree was constructed based on the complete SaV genomic sequences using the unweighted pair-group method (UPGMA) (45) to illustrate the evolutionary relationship between the recombinant strain YNAN (highlighted in red block) and a major parent strain with similar sequence to MK965898 (highlighted in green block) in the region of 1–5195 and 6352–7583 nt (A), and between the recombinant strain YNAN (highlighted in red block) and a minor parent strain with similar sequence to FJ387164 (highlighted in blue block) in the region of 5196–6351 nt (B). (C) Analysis of the complete genomic sequences of MK965898 (green line) and FJ387164 (pink line), with YNAN as the query sequence. Red, green, and blue color shades are used to label the recombinant, potential major, and potential minor parent strains on each tree, respectively. In the RDP plots, turquoise blue lines are potential major parent-recombinant; purple lines are potential minor parent-recombinant; yellow lines are potential major parent-potential minor parent. The value below the red barcode indicates the confidence of the RDP analysis, with the smaller value standing for higher probability. [file Image_2.JPEG]
