# Supplementary material for: High Genetic Diversity of Porcine Sapovirus From Diarrheic Piglets in Yunnan Province, China
Source: Front Vet Sci. 2022 Jul 7;9:854905. doi: 10.3389/fvets.2022.854905 (PMC9300989; doi:10.3389/fvets.2022.854905)
Supplement: Supplementary file 3 [file Table_1.docx]

**Supplementary Table 1 Primers used in this study to amplify genotype III PoSaV**

| Primer names | Primer sequence (5'→3') | Amplification region (bp) | Product length  (bp) |
| --- | --- | --- | --- |
| PoSaV-3F1 | GTGATCGTGATGGCTAATTGCC | 1-1104 | 1104 |
| PoSaV-3R1 | GTARTCCACTATCCACATGGC |  |  |
| PoSaV-3F2 | GTGGATAGTGGACTACTTCAAAC | 1089-1970 | 882 |
| PoSaV-3R2 | TTGATGTCAGCGGCCTCACTG |  |  |
| PoSaV-3F3 | AACAGTGGYTAGTGGACAACC | 1757-2708 | 952 |
| PoSaV-3R3 | GCYTTGAGAATGTCCCACCA |  |  |
| PoSaV-3F4 | CTGARTGCTCAYTGCTCAGGAC | 2543-3806 | 1264 |
| PoSaV-3R4 | GGTTGYAGGTTRTTGGCAATC |  |  |
| PoSaV-3F5 | TCMCAACACCARATGATTGCC | 3772-4663 | 892 |
| PoSaV-3R5 | TCARGCCGTACACGCAATCATC |  |  |
| PoSaV-3F6 | GGAGTTCAARGTGCCCTACATG | 4581-5351 | 771 |
| PoSaV-3R6 | GTGGTCCAGGTGACATTGGT |  |  |
| PoSaV-3F7 | CGTACAACGCRTGGTACGGT | 5082-6820 | 1739 |
| PoSaV-3R7 | AGATCACCTAGCAGGCCAGC |  |  |
| PoSaV-3F8 | CGCCTCATTGGACCAAGTGGGA | 6721-7350 | 630 |
| PoSaV-3R8 | GCCCCACAGCCGCCACACTGTGT |  |  |
