# Supplementary material for: High Genetic Diversity of Porcine Sapovirus From Diarrheic Piglets in Yunnan Province, China
Source: Front Vet Sci. 2022 Jul 7;9:854905. doi: 10.3389/fvets.2022.854905 (PMC9300989; doi:10.3389/fvets.2022.854905)
Supplement: Supplementary file 4 [file Table_2.docx]

**Supplementary Table 2 Primers used in this study to amplify genotype V PoSaV**

| Primer names | Primer sequence (5'→3') | Amplification  region (bp) | Product length  (bp) |
| --- | --- | --- | --- |
| PoSaV-5F1 | GTGATCACTTTGAGATGGCTTC | 1-940 | 940 |
| PoSaV-5R1 | ACGAAGAARTCATGAAGGGTGT |  |  |
| PoSaV-5F2 | GAGGGYTTTGCGGCTTCAGT | 873-1591 | 719 |
| PoSaV-5R2 | CGGTCRGTGTCAAACTCATCC |  |  |
| PoSaV-5F3 | GAGGTTGCRGTTTGGGATGAG | 1557-2362 | 806 |
| PoSaV-5R3 | TGRAGCAGTGTAAAGGGTGACCA |  |  |
| PoSaV-5F4 | GATGTAYCAAGTSTGGTCACC | 2327-3224 | 898 |
| PoSaV-5R4 | TATGGGTGTTGTGGCCTCATC |  |  |
| PoSaV-5F5 | ATGACRGCCGACCAGTTCCTC | 2958-3571 | 614 |
| PoSaV-5R5 | AAATAGGGAAGTCCACAATCCC |  |  |
| PoSaV-5F6 | ACCAAGAAAGGGGATTGTGG | 3540-4724 | 1185 |
| PoSaV-5R6 | GAAYCCATACAAGCAGTCATCAC |  |  |
| PoSaV-5F7 | CACACSTACGGTGATGACTGC | 4686-5199 | 514 |
| PoSaV-5R7 | ACACTACTAGCCCAAAGCCTTC |  |  |
| PoSaV-5F8 | ACCAGAAGTGCCCAGCACCAATG | 5156-6916 | 1761 |
| PoSaV-5R8 | CCAACTCATGACCATGCAATGC |  |  |
| PoSaV-5F9 | TCTTATTGGCCCACATGGGAG | 6860-7496 | 637 |
| PoSaV-5R9 | CCATCTTGCTAAARGTGTCCTAG |  |  |
