# Supplementary material for: High Genetic Diversity of Porcine Sapovirus From Diarrheic Piglets in Yunnan Province, China
Source: Front Vet Sci. 2022 Jul 7;9:854905. doi: 10.3389/fvets.2022.854905 (PMC9300989; doi:10.3389/fvets.2022.854905)
Supplement: Supplementary file 5 [file Table_3.docx]

**Supplementary Table 3 Reference sequences of porcine sapovirus used in this study**

| Accession number | Place/year | Genotype | Accession number | Place/year | Genotype |
| --- | --- | --- | --- | --- | --- |
| AY237422 | Japan/2008 | GI | MK965900 | USA/2019 | GIII |
| KJ858686 | Congo/2011 | GI | MK965901 | USA/2015 | GIII |
| MK111630 | Guangdong, China/2014 | GI | MK965902 | USA/2018 | GIII |
| AY237420 | Thailand/2005 | GII | MK965904 | USA/2019 | GIII |
| AY646855 | Thailand/2004 | GII | MK965905 | USA/2019 | GIII |
| AB242875 | Japan/2008 | GIII | AJ786349 | Japan/1999 | GIV |
| AY425671 | USA/2004 | GIII | DQ058829 | Japan/2002 | GIV |
| AY823308 | USA/2016 | GIII | DQ125333 | Sweden/2004 | GIV |
| AY826426 | USA/2003 | GIII | AB521771 | Japan/2008 | GV |
| DQ056363 | Venezuela/2016 | GIII | AB521772 | Japan/2008 | GV |
| EU381230 | Hunan, China/2006 | GIII | AB775659 | Japan/2012 | GV |
| FJ387164 | Shanghai, China/2008 | GIII | AY646856 | Thailand/2006 | GV |
| HM346628 | Korea/2007 | GIII | JN420370 | USA/2010 | GV |
| HM346629 | Korea/2007 | GIII | KX000383 | USA/2009 | GV |
| JX678943 | Anhui, China/2009 | GIII | LC483440 | Japan/2018 | GV |
| KF204570 | Gansu, China/2012 | GIII | MN161594 | Jiangsu, China/2016 | GV |
| KT922087 | USA/1979 (Cowden) | GIII | NC027026 | Japan/2012 | GV |
| KT922089 | USA/2016 | GIII | KJ508818 | USA/2000 | GVI |
| KT945133 | USA/2002 | GIII | LC215888 | Japan/2016 | GVI |
| KX688105 | Shanghai, China/2010 | GIII | MK965903 | USA/2018 | GVI |
| KX688106 | Shanghai, China/2010 | GIII | AB221130 | Japan/2008 | GVII |
| KX688107 | Shanghai, China/2015 | GIII | KX000384 | USA/2011 | GVII |
| LC215874 | Japan/2017 | GIII | KC309417 | USA/2009 | GVIII |
| LC215875 | Japan/2017 | GIII | KC309419 | USA/2009 | GVIII |
| LC215876 | Japan/2017 | GIII | LC469052 | Japan/2018 | GXI |
| LC215877 | Japan/2017 | GIII | AY144337 | Canada/2003 | GXII |
| LC215878 | Japan/2017 | GIII | KX000385 | USA/1999 | GXII |
| LC215879 | Japan/2017 | GIII | JN387134 | USA/2010 | GXIII |
| LC215880 | Japan/2017 | GIII | JN899072 | Hongkong, China/2012 | GXIV |
| LC215881 | Japan/2017 | GIII | JN899074 | Hongkong, China/2012 | GXIV |
| LC215882 | Japan/2017 | GIII | JN899075 | Hongkong, China/2012 | GXIV |
| LC215883 | Japan/2017 | GIII | KJ641701 | Beijing, China/2012 | GXVI |
| LC215884 | Japan/2017 | GIII | KJ641703 | Beijing, China/2010 | GXVII |
| MF766259 | Shanghai, China/2015 | GIII | KX759619 | Belgium/2014 | GXVIII |
| MK378994 | Jilin, China/2017 | GIII | KX759623 | Belgium/2014 | GXVIII |
| MK962340 | Spain/2017 | GIII | KX759620 | Belgium/2014 | GXIX |
| MK965898 | USA/2019 | GIII | KX759622 | Belgium/2014 | GXIX |
| MK965899 | USA/2019 | GIII |  |  |  |
