# Supplementary material for: High Genetic Diversity of Porcine Sapovirus From Diarrheic Piglets in Yunnan Province, China
Source: Front Vet Sci. 2022 Jul 7;9:854905. doi: 10.3389/fvets.2022.854905 (PMC9300989; doi:10.3389/fvets.2022.854905)
Supplement: Supplementary file 6 [file Table_4.docx]

**Supplementary Table 4 Accession numbers of the 21 porcine sapovirus sequences determined in this study**

| Number | Sample name | Region | Genotype | GenBank accession number |
| --- | --- | --- | --- | --- |
| 1 | YNQB | Wenshan | GV | OK485997 |
| 2 | YNJD | Pu’er | GIII | OK485998 |
| 3 | YNAN | Kunming | GIII | OK484999 |
| 4 | YNLH | Dehong | GIII | OK485000 |
| 5 | YNAN1 | Kunming | GIII | OK485002 |
| 6 | YNXSBN1 | Xishuangbanna | GIII | OK485003 |
| 7 | YNBS1 | Baoshan | GIII | OK485004 |
| 8 | YNDY1 | Chuxiong | GIII | OK485005 |
| 9 | YNDY2 | Chuxiong | GIII | OK485006 |
| 10 | YNDC1 | Kunming | GIII | OK485007 |
| 11 | YNLJ1 | Lijiang | GIII | OK485008 |
| 12 | YNLF1 | Chuxiong | GIII | OK485009 |
| 13 | YNMZ1 | Honghe | GIII | OK485010 |
| 14 | YNNJ1 | Nujiang | GIII | OK485011 |
| 15 | YNSJ1 | Zhaotong | GIII | OK485012 |
| 16 | YNTH1 | Yuxi | GIII | OK485013 |
| 17 | YNXW1 | Qujing | GIII | OK485014 |
| 18 | YNYM1 | Yuxi | GIII | OK485015 |
| 19 | YNYM2 | Yuxi | GIII | OK485016 |
| 20 | YNYM3 | Yuxi | GIII | OK485017 |
| 21 | YNYM4 | Yuxi | GIII | OK485018 |
