# Supplementary material for: High Genetic Diversity of Porcine Sapovirus From Diarrheic Piglets in Yunnan Province, China
Source: Front Vet Sci. 2022 Jul 7;9:854905. doi: 10.3389/fvets.2022.854905 (PMC9300989; doi:10.3389/fvets.2022.854905)
Supplement: Supplementary file 7 [file Data_Sheet_1.docx]

>YNQB [organism=Sapovirus GV.] Sapovirus GV gene, complete cdsGTGATCACTTTGAGATGGCTTCAAAGCCATTTCGAAGTAATGAGCGTAATGCTCGTTTTGAAATTAATCTCCTCCACAGGTGCTACCTCAGGGTAGCACCACGTGAAAGGTTTTTGCCTGACTCCTCACTGGAGTCTATTTGGTGTTATTACAACTTACACACCGTGCCGCAGGGCACTCCCTATGTCGAGCCCCACTTTCAGTCCGAAGGGCTCTTGTCCCGCCTTTTTGGCACTGCCGGTTCCCCTGGTTTGGATTCCCAGACAGCCTACAAGGAGCTGTTCGGTTTCCTTACTGAGGAACAGCTCCCACTTTCCCTTGATGATCTTGCAAAACTACAGGGGGAGTTGACGACGGCCCTTCGAACCAGTGGCAATGAGTTTGTTACCAAGCACGGCAAATCTCAGGTTCAGTCTCTTTTGCGGCAACTTGACCAGCTTGTCCCGCGTGATGTTTCGGAGCGGGAAGCAGCGCGGCGTTTGGAGTTTGAGCGCGCTACTGCTGAAATGTTTAGTGAGTTACCTAACACAGACTCCTTTACGGAGGATGACTGGAAACCTTACTGGTTCGCAATGTGGAAGAGAGTCATTAAAGGGTGTCGGGGCTATTACCATGCAATACCAAGGTGGTCATCATTCAAGACGCGATTGGCACGTGCGACGGAACCCATCAGACAATTGTTAGGTGTTATGGCGGAATCCTTCAACTATTGTTTGTCTTGTGACCCGCGAATTGTGGCGATGGATCTCGTCGCGGCCCTCAAACCCACAGTGTTGACCATGATCTATCAACAGCATCGCAACACTCCCCAGGGATGGCTTGCCACCTTAACTGCCTTATGGGAAGTTCACAGCCCAAAACTTTCCCACGTGGAGGGCTTCGCTGCTTCAGTCACCACTACTTTGGGCCTGTTGGTTAACACCCTTCATGATTTCTTTGTTAAACTATGTCACCACATGGATTCAACGCAAACGCCCCAAGGGCCCACGACGTCAGGTTGGGCGGTCATTGTGGCAGGGTTGTTTGCCCTCCTTTTGAAGCTATCATGTATCCCAAAAGTCTTTAGCCACTGGCACACACTGCTTAAGCTCGCTGGTGGCATCACCACGGTTGTGGGTGCAGGACGAGCAGTCGACTGGGTCCTCTCCAAAATCCGCGAGTCGCGCAACGCCTCCATGTGCAAACAGTTCCTTGCTCGCGTGTCAGCTCTGATTGAGGTGCACTACTCGCGTACCGTTGCTGGGGTTACTGAAAACACTGAGCTGCTTAAGTGTTTTGACCAACTGGTGGATGAGGGTGAAGAGCTGGTTGCTGAGCTGGGTGGTGGTTCACTCTCCGCTATCATCCGCTCGGGTGTCGAGACTCTCCAGCGAGTCGCCGCGGAGATTAAGGCCACCATTCAACTTGACAATCCTCGTAAAGTCCCTGTGTGTGTGGTCTTCTGTGGCCCACCTGGCATTGGGAAAACCTCCCTTGCCTATCACATGGCAAAGGGATATGGTCTGACTTCCAACTTTTCCCTTGCTAATGACCACCATGATGGATATACGGGAAATGAGGTTGCAGTGTGGGATGAGTTTGACACTGACCGCGATGGTAAGTTTGTTGAACAAATGATATCGCTTGTTAATTCACAGCCATGTGTTTTCAATTGTGATCGCCCAGAGAACAAGGGCAAGCTGTTCACCTCAAACTACATATTTTGCACAACCAACTATGCAACCAGTGTGCTTCCTGAGAACCCCCGTGCTGGAGCGTTTTACCGCCGCATCATCACTGTTGATGTGTCTGCCCCCGAGATTGAAGACTGGATGTCCAAGAATCCCGGGAAGTCCCCCCCAAAGACACTGTACCGGCAGGATTGCTCGCACCTCAAACTAATGTTGAGACCCTATCTTGGGTATAACCCGGATGGTGACACCCTTTCAGGTAAACGTGTGCGCCCCACCCCAATTACTATTAAGGCACTTCATGACTTGATTGATAAGAAGTTTGAGGAGCAGTCTGCGGAGCATCGTGGGCTTTGGATCACAGTGCCTAAGCGTCAGGTCCAACAGGCGCTGGCTGCAGTTAAGAAATTTTGTGTGGTCAACCAAGCACTGTGCCATGTTACATCAACTCCAACCCATGAGGTTTTGCAATGCGCTGTCTTTTCTTGCATTGTGGTTTCAAGTGACAATCCCCCTGGCAGCGTTCCTTTGCTCCACATCAAAGATGCCCAACTTGACTTGGACTCCGCAGGAAACTCACAAGCCAGCATCAGCGAATCACTAATGGGTCTCTTTACTACCCAGCAGAGAGTGAGTTCCAACTTCCAACGTAAAGTGATGTACCAAGTTTGGTCACCGTTCACTCTCCTTCAGACCGAGACTCTAAACACACAATCATTACCACCAGTTCGGCGTATTATTTACGCTGACACTGCGCTTGATTTCCTCAATGGTTTGCGCCACCATCTTGGCTTCAGTTCGCTGCCAGGGTTGTGGCGAGCAATACGTCACTTGCCAGACTGTTCTTCAATGATTAACTGGATTACTGATCATCTTACCCAGGTGCGATTCCCAGAGAATCCAGAGTCAACCTTGTTTCGCACAGGGGATGGTGATGTCATCTTTTATACTTTTGGGTCCTTTTATGTCCTTGGCACTACTGCGCGTGTGCCTGCCGTAACTGGTGACACTCTGAACCCGTTACCCAACATCCCTCTCAAAATGACTTGGTTTGAAACCCTTAGAGCATTGTGTACGTCTGCCTTAAGGTTGTTTACTGCAGTGGCCCCTTTCTTTCTTGCGATGGTTAATGTCTCATATCTACGCGCCCGTGGGAGTCGTGATGAGGAGGCTAAAGGTAAAACGAAGCATGGGCGTGGAGCTCGCCACGCACGTGGGAGAGCCACTGCACTGAATGATGATGAGTACAACGAGTGGATGGATCTCCGTCGTGACTGGCGTGAGGAAATGATGGCTGACCAGTTCCTCCAACTCCGGGACGAGGCGTATGAAGGAATCATCAATGATAGGACTCAGCGCTATGGGGCATGGTTACAGCTGCGCAATACTCGGTTGAGCGCTAATGCATACCAACACGCTACAATTATTGGGAAGGGAGGCGTGCGTGAAGAGTTGATACGCACGCAAATGTTGTCAGCACCCAAGAAAGGCAAATGGAACCGTGGAGCGGACTCAAATCCAATGAACTACTTCGATGAGGCCACAACACCCATAGTGGAGTTCCAATCATGCGGCGAGCATGTTGGGTGGGGTGTACACATTGGCAATGGGCGTGTCGTCACTGTCACCCATGTGGCCACGTCGTCAGATACAGTTGAAGGTCACCCTTTCCGCGTCAGTGAAACAGACGGCGAGACCTGCTATGTCCTAACATCCTTACAATCGCATCCCTATTACCAGCTCGGGCAGGGTGCGCCTGTTTACTTCACCACGCGCTTTCACCCCGTTCTTGTCATCAGCGAGGGGCAGTTTGACACACCTGCAACAACTGTGGTGGGGTACCATCTGCGCATTGTCAACAGTTACCCAACCAAGAAAGGGGATTGTGGTCTCCCATACTTCAATGCTCAACGCCAGGTCGTTGCTCTCCATGCTGCTGGCAGCACTGATGGAGCCACCAAGCTTGCCCAGCGTATAGTTACAAAGGTTGACATTGGGGAAACTTTTGTGTGGAAGGGTTTGCCCGTCATACGTGGAAAGGACGTTGGGGGCATGCCTACTGGCACTCGCTACCACCGATCGCCTGCATGGCCTGAGATCTCACCAGACGAAACACATGCTCCCGCACCCTTTGGGTCTGGTGACACACGTTACAACTTCTCTCAAGTCGAAATGCTGGTTGGTAACCTCAAGCCATATCTGGAACAAACTCCTGGGATTCCGCCCGGTTTACTCAACCGTGCGGTTGTTCATGCTCGGAGTTACTTGCAGTCAATAATTGGGACAACCCTTAGTGATCCCTTGTCCTTCAACATGGCCGCAGCTCTGCTTGAGAAGTCAACATCGTGCGGGCCACATGTCCCTGGGCTTAAAGGTGACTACTGGGATGAAGAGACATGCCAATATACCGGGGCACTCCGAGAACATCTTGAGAGTGTTTGGAATGACGCCATGATTGGTCGGGCACCGTCACATGATTACAAGCTTGCCCTTAAGGACGAGCTGCGGCCTGTTGAGAAGAACATGCAAGGAAAAAGACGCTTACTGTGGGGGGCGGATGCTGGGCTTACGTTTGTGTGTTGTGCTGCTTTGAAACCCATTGCAAACAGGCTACAGGCTGTGGTGCCAATGACACCAATCTCCGTTGGGATCAACATGGACTCTGGTCACATTGACGTCCTCAATGAATCATTTAAGGGTCGCGTGCTCTATGCACTGGACTATAGTAAGTGGGACAGTACCCAGTCACCATCTGTCACCGCTGCCTCCTTGGAGATACTTAGTTCCTTCATGACACCCTCGCCCATAGTTTCATCCGCCGTAGAAGCTCTCAAAGCCCCCGCACGTGGGATGATTAACGATGCAATTTTTGTCACCCGAAATGGCCTTCCTTCAGGTATGCCCTTCACTAGTGTCGTTAACTCACTCAACCACATGTTGTACATTTGTGCCGCTGTCCTTTCGGCCTATGAAAGCAGGGGTCTCCCTTATAGTGACAACGTGTTCCATGTGGAGACCATTCACACGTATGGTGATGACTGCTTGTACGGATTCACCCCAGCCACGGCATCGCTGGCAGACAGTGTGATAGACAACCTGCGGTCTTTTGGGTTGCGTCCAACAGCTGCTGACAAATCGTCAAACATTGCCCCAGTCCAAGTGCCAGTCTTTCTCAAGCGCACTTTCCAAGTTACCCCCCATGGTTTGCGCGCCCTGCTTGACCAATCTTCAATTCTTCGACAGTTCTACTGGGTCAAGGCCCAGAGGACTTGCGAGGTTAATTCCCCCCCAACTCTTGACGTGCGCGCACGATCATCACAACTCGAAGTTGCCTTGGCATATGCTAGCCAGCATGGTCACGCGTTCTTTGACTCTGTCGTTGCTATTGCACACCACACGGCAGAGTGTGAGGGGTATTCTCTGGTGAACACCAACTACGAGCAGGCCGTCGCGTGTTACAACAGCTGGTTTATTGGTGGGTCTGCACCAGAAGTGCCCAGCACCAATGAAGGCTTTGGGCTAGTAGTGTTTGAAATGGAGGGCAATCGCTCTCAGCAGGGGATAGCCACCCCATCTCAGGATGCGCCACGGAACACCCAAGTAGCACCACCTGGTACCATCGGACCCTCAGACGCTGCATTGGTCCCTGTTAACCCGGAACAACCGAATTTGCCTGCACAACGAGCCGAGCTGGCAATCGCAACTGGTGCTGTGTCATCAAATGTCCCTGAGAGTGTTCGTCGCTGTTATGCGCTTTTGCGCACCGTGCCTTGGAATACACGCCAACCCCAAGGAACCCTTCTAACAGCAGTCTCTTTGCACCCAAACATCAATCCCTACACATCCCACCTCTCACAAATGTATGCCGGGTGGGGTGGTTCAATGGAGGTCAGGGTCACAGTCTCTGGGTCTGGCTTGTATGCTGGAAAATTGTTGTGTGGTGTCCTCCCCCCTGGCATTGAACCAACCACTGTGTTGAACCCTGGGGTCCTTCCGCATGCAATGGTTGATGCCCGTTTAACCGACCCCGCCGTTTTCAATTTGCCCGATGTGCGTGCGGTCGACTACCACCGCACTGATGGTGAGGAATCAACTGTCACCTTGGGCATTTGGGTTATGCAGCCTCTGATCAACCCCTTTGGCAGCACTGAAGCTGTGTCAACCGCTTGGGTCTCCATTGAAACCATGCCGGGCGCAGATTTTGACTTGTGCTTGCTTAAACCTCCCCAACAGCGGATGGCAAATGGTGCTTCACCCGACTCCTTGCTGCCACGACGCCTGCAGAGACACCGGGGCAATCGTGCTGGTGGGTTCATTGTGGGTGCAGTGATAGTTGGTTCAGCCAACCAAATCAACCGTCACTTTAGTGCCACGGGCACTACCCTTGGTTGGTCGACTGCCGGCGTTGAACCCATGGCTTTTGCAATAGGCGAGATGCAAACTGGAACCAACACAAACCCAAAGGTGGGCTACCATATTGAGGTGGGCAGTGATCAACGTGGCCCTATATATCCAAACATCGTCAACCAGTGGCCAGATTTTGCTGTCAATTCCAGCTACACATGGAACGATAATGAGATCATCCCAACGAACGCTGTGGCTGGCACAGTTATGCACTTCCAAGACAATGGTGATGTTAGTGAAACAGAAATTGGCCGCATGTTTCCAGTCGCAATGTACTCCACTGGCACTGCACGTGGTAGGCTTGTGGCCGCGTTCAACCCGGCGAGCATGTACCTCGTACGTACGGACAGTGTCAACGCCCCAAATGGCTGGGTCACCTCGGGGGCTAATAATGGGAATGGATATTTCACACCACTATGGGGTCATGGGAACGGCAACATGATTGGAGACAAGATATGTAACATGGAAGGGGCAAATTATGTCTTCAATTCAAGTGGCCCAGATAACATCCTCTTGTGGAAGGAGCGGATCTTTTCAGATTACAATGGTAGTTGCGTTTTGTACAGCTCACAATTGGATACTACCGCAACCACCCTCCAGGAAGGTCCAGTCAACATCCCAGAGAACATGATGGCAGTGTACAATGCTGAGTCAAACTCGGCCAATTTTCAACTCGGCATTCGCCGCGATGGGTATGCTGTCACTGGAGGCACAATTGGCACTCACGTAGACCTTGATCCAGAAACTACCTTTACTTTTGCTGGTCTTTACCCCTTAACCTCATCTCTTATTGGCCCACATGGGAGTGAAGGACGGGCCCGCATTGTATGGTCATGAGTTGGATTGTAGGTGCTCTTCAGACCCTTGGTGGTCTCACCGATGTGGCCTCATCAATATCTGGGGCGGTTTACCAACAACGCCATATAAATCAGCTTCGGCGTGCAAATGATTTGCAAGCAGAATGGATGGCTAGAAATGAACAACTACAACGTGATGCAATGGCTATGAACATGGAACTTTCCACTCGAGGCCCAGCCTTACGTGTCCAATCAGCAATTGATGCTGGGTTTGATCCACTCAGTGCCCGCCAAATCGCGGGCTCAAATGAGCGTCGCATTTCTGGCTATCTTGAGCAGCCAGTGCGTACAGTGGATCGTGCTGTTGCAACACAGTCTCATGGTAATCTTGTGGGTCTTTCCAATGCTATGAGTACCTTTTCCCACGGCACTCGCTTTGGGATGCGGGCCCCTAGTCGGGCACAGCCGAGTGTCACCACTGGGCGCCCTGTCGTCAATCTTGGGCAGCCTTCGGGCGAGAGCCGTGTGTAGTGGTTTCCTATTCTATTTTGCTTGGACTTCTATTTCTTTTATTTTCTTTCCATTCTAGGTTTCCTCGTTCGCTAGGACACTTTTAGCAAGATGG

>YNJD [organism=Sapovirus GIII.] Sapovirus GIII gene, complete cdsGTGATCGTGATGGCTAATTGCCGTCCGTTGCCTATTGGGCATTTGCCCAACAGGATTTTTGGCACCCCACGACTCACCCCAGGGTGGGTGTGGGCGTGCACCAAAGGAGCTGCATTCAAATTAGAGTGGCTCCAAGATCCGGTGGTCATACGGCCCCCGGAGATTTTTGTTGCGCAAGGGGTGGTTGATGACTTCTTCCGCCCCAAGCGCGTTTTGCAGGGTGACCCACAACTCATTGCACAGGTTTTGCTGGGCGACGCCAACGGGCCACTCGTTGGTCCCGTGTCCATGCAGCAGCTAACATCACTGCTGCATGAGGTGTCGCAGGCCCTGAATGACCACAAGCACCCACTGGCTGGTAGATACACTAGAGCCTCACTGCAAAGGTATGCAGACACACTGTCCAATTACATACCCCTTGTTGACATCCTAACAGGACCTAAAGACCTCACTCCGCGTGATGTTCTCGAGCAACTCGCGGCAGGACGCGAATGGGAATGTGTGCCAGACAGTGCCCTAAAGAAGGTTTTCAGGGACATGTGGCAGCACATTTATGAGGGATGTGACAGTGTGTATATAAAGCTGCAAGATGTAAAACGCAAAATGCCACACATTGACACCACTGTGCTCAAGCAGTTTTTCATCACACTTACAGATACCATATCCATGGCAACAGCCTTGGACACCAAGACGTGGCTTGCACACATATTAGGTTGGCTCAAACCCACCTGCCTAGTGATGATCATGCAACAGCATGTCAACTCTCCCCAGGGGTGGGCTGCCACGCTAACTGCTCTAGCCGAGTTGTATTATGGTATCATGCCACTGACAGAGACGCTTGGCTCCATTGCCAGTTGGGTGACCGATAAGTTCGCTGATATGGCGACCAACACCTGGAGCAAGTTTAAGACCTGGTGGGACAGCCTATATACACCACAGGCTGGCAATGACCTTATCATATTGGGGGGTGTGGTTGGGCTGGTGTACTTCATGGTGTTTGGTGACGCGCCCACCCAAATGTTCACCAAAAAGTTGATGAAGGTGTGTGGTTTTATCACCTCCACAGTGGCTGCCATCAAAGCGGCCATGTGGATAGTGGATTACTTCAAACAGCGTGAACATGAGCATCAAGTTCGTGTCACGCTGGCCAGGTGGGCTGCATTGCAAGAGGTGATCAAACAGAACAGGTGTGCTGGCCTGTCAGAAGTGACCAAACTGAAAGAGTGCTGTGAAGTGTTGTTGAACGAGGTCACCGAGCTAATGTATAAACTCGGTGCCTCCCCTTTAGCAGGATTGATACGCAGCACCTCTGACGTCATACAAACTACCATCAATGACCTGGCCCAATTGATGGCTTATGACACCCAGCGCAAGCCACCTGCCATGATAGTGTTTGGTGGACCTCCAGGCATAGGTAAGACTAGGCTAGTGGAAGCTCTAGCAAGACAGTTGGGAGAAGTCAGTCATTTCACCATGACCGTTGACCACTATGACACCTACACTGGCAACACTGTTGCAATCTGGGATGAGTTTGACGTGGACTCAAAACAAGCCTTCATTGAGGCCACTATTGGTATTGTTAACTGTGCCCCTTACCCCTTGAACTGTGATCGGCCAGAGGCAAAGGGCAGGGTGTTTACCTCCCAGTACATCTTGGCTACCACCAATTGTCCAACCCCAGTCATGCCCGACCACCCAAGAGCCATGGCCTTTTGGCGCCGCATAACATTTATTGATGTGACAGCCCCAACAATTGAACAGTGGTTGGTGGACAACCCGGGCCGCAAGGCTCCTGCCTCCCTCTTCAAGGATGACTTTTCTCACCTGCAGTGTTCAGTGCGTGGTTACACCGCTTATGATGAAAAAGGCAACACACTGAGTGGTAAAGTGGCCAGGGCTAAGTATGTGTCAGTGAACAACCTTCTCGATTTGATCAAAGAGAAGTATAACAGTGAGGCCACTGACATCAAACACTTGTGGTTCACTGTCCCACAGGCCATACACAAACAAGCCCGTGATATCATACTGGGCTGGCTGCGGTTCCACTCTTACCCGAACACAGTGGCAGACAACATACCACTGTCCGAGGTCAGGGACCCCACGTGCTTTGGGTATGTGGTCATTTCTGACGTGGATCCGCCACGACATGTGAGTGACTACGTTGCCCACATTGAGGTGGAATCAGTACTACGCACTGACATTATGGACCTGTTAAGGGAAGGTGGTGGCGGCTTGTTTAGGGCCCTAAAGGTGAAGAGCGCCCCCCGCAACAGTGTTATCAACAAGGTCATGATGCAAGCACACCACACAACCTTGCAGGTGCTCACCAATCAGGAACCAAACCCGCCAAACTTGCCGCGTCCCAGGCGCTTGGTGTTTGTGGAGTCACCCATTGACATCATCAGCGCGTTGCGGCACCACGTCGGTTTTTGCACCATCCCCGGCATTGTCAAGATGGTCACATCTGGGGTGGGTCTGGGTGTCGAAAACCTTGGAAACTTCTTGCAATCCATCGCTGGCAATGTTCGGTTCCCCCTGCAATCTGAGTGTTCATTGCTCAGGACACCCGGTGGTGACGTCCTGTTTTACACCTCAGGACAGGCTGCCGTTTGGGCCACACCTGCGCGCTTCCCGATTGTTACCCCAGGTGAGGCGTCAGTTGGCAAGGATGTTTGTGCAGAGTCTTCCTGGTGGGATATTCTCAAAGCACTTTTCAGCACACTTGTGGTGGCATTCGGGCCAATAGCCACACTGGTGTTGACAGCACACAACCTGGCATATCTGAACGCCCGTGAAGGCACACTTAGTGAGGCAAAGGGGAAAAATAAACGTGGGCGTGGCGCACGCAAAGCCATTGCTCTCAGGGATGATGAGTATGACGAGTGGCAGGACATCATACGTGACTGGCGCAAAGAAATGACTGTCCAACAGTTCCTGGACCTCAAAGAGCGCGCCCTGTCCGGTGCCTCCGACCCCGACTCGCAGAGGTATAACGCCTGGCTTGAACTGCGTGCGAAGAGATTGAGTGCTGGGGCCTACCAGCACGCGGTTGTGGACATCATTGGAAAATCAGGCCACAGGCGTGAAGTGATTCGCACACAGATGATGCGAGCCCCTCGTGAACCAAAACCAGACACCTACGACAGTGAGGGGCGTGGTTATGTGGTGCCCATGACCGCTCAGGAAAAACATACAGGCTGGTCAGTGCATATTGGTAATGGCAGGTTGGTCACTTGCACACACGTGGCCAACATGTGTGATCGTGTGGGTGATGTTGAGTTCAAAATCACTGAAACAGACCGTGACACATGCATCATCACCGCCCCACTCGGTCACCTGCCCAGTGTTGCCCTTGGGGATGGACCACCAGCCTTGTACACTACTAATTTTCACCCCATCCGTGTGCTGGATGAGGGCAGTTGGGACACTACAACCACCCGCGTCACCGGCTGGCGTGTTGTCATTAACAACGGGACCACGACTGCCCCTGGTGATTGTGGCCAACCGTACTTGAATGCACGCCGGCAGCTTGTGGGGGTGCATGCGGCAACAAGCACATGCGGGGTGAAGAAACTCGTTTCCAGGGTGCAAACTAAAAGAGCATCAAAAGCCACATTTCCTTGGAAGGGTTTACCTGTCACCAACATGCCTGATGCTGGGGGTTTGCCCACTGGCACGCGGTACCACCGCTCCACTGCATGGCCCAAGCAGCTGCCAGAGGAAACGCACGCCCCCGCCCCTTATGGTGTGAATGATCCCCGGTACCCTTTCTCCCAACACCAAATGATTGCCAACAATCTCCAACCTTACACTGCAACACCAACAAACTTGGACAAAACCTTGCTGCAGCGTGCTGTGCGACACACCAAGGGCTATTTGGATCAGGTCATTGGCACTCACAGGTCACCAAACCTCACCTACGCTGCAGCAGTCCAGTCCATGGCTCATGACACAGCATGTGGACCAAACTTGCCAGGTAGGAAGAAGGACTACATGACAGATGAGGGTGAACCAATAGGACCCTTGAAAGACATGTTGGAGGAGGCATGGGACCTTGCTCACCGGGGGGTCCCGAGGCGGCATGAGTACAAACTGGCTTTAAAAGACGAGCTGCGACCCATTGCCAAGAACGAGCAGGGCAAGCGTCGTCTACTGTGGGGTTGTGACGCCGGTGTCTCAATGGTGGCCAATGCAGTCTTCAAGCCTGTGGCAGAAAGGTTGGCTGACACAGTGCCCATGCATCCCGTTGCGGTGGGCATCTGCATGGACAGCCCCCAGGTTGAGAAAATGAACCAGGCATTAACGGGCAGTGTGCTGTACTGCATGGATTACAGCAAATGGGACTCCACGCAAAACCCGGCAGTGACGAGCGCCAGTGTCGACATCTTGGCCTCTTATGCCGAGGATACACCCCTCACCTCAGCAGCTATAGCCACACTGTGTTCACCGGCCGTCGGCCGGCTTGACGACATTGGTTTGACAGTCACAACAGGACTCCCTTCTGGCATGCCGTTCACTAGTGTCATAAACTCTGTCAACCACATGATATACTTCGCCATGGCCGTGCTTGAAGCTTATGAGGAGTTCAGGGTGCCCTACATGGGAAACATTTTTGACAATGAGACTGTCTACACATATGGGGATGATTGTGTGTACGGCCTGACACCTGCAACAGCTTCCATCATGCCGGTGGTGGTCAAAAATCTGACCAGTTACGGGTTGGTTCCAACAGCTGCAGACAAATCTCAAACCATTGAGCCCACTGACACGCCAGTGTTCCTCAAGCGTACCTTTGCACAAACACCCTTTGGGCTGCGTGCACTGCTTGACGAAACAAGTCTAGCGCGGCAGTGCTACTGGGTTAAAGCTAACCGCACTAATGATCTCTTTGAACCAGCGGCCGTTGATGTGGAAATCAGGAAAAACCAATTGGAGGTCATGCTCGCATACGCCAGCCAACATCCAAGGGCAGTGTTTGACAAGCTTGCAAACATGGTTGAGGTGACTGCACACACTGAGGGTTACCAAGTTGTGAATATAGACTGGGCCAACGCAGTGGCAACGTACAACGCATGGTACGGTGGCACTGAAGGTGGCCGTGCTCCCACCAGTGAAGATGAAGAGCCAGAAGTGTTCGTGATGGAGGCGCCTGCCCCAACCCGTCCGGTTGCAAGCAACCCAGAAGGCACTGAGACCAGTAATGAGTCCAGACCAGTTCAGCCACCTGGGCCTACGCCCGTGGCCGCAGCACAGGCTCTAGAGATGGCCGTTGCCACGGGGCAAATCAATGATACCATCCCCAGTGTGGTGAGGGAAACTTTTAGCACCTACACCAATGTCACCTGGACCACACGTCAGCCTGCAGGAACCTTACTTGCCCGGATGACCCTGGGGCCAGGTCTGAACCCTTACACACTCCACCTGTCTGCCATGTGGGCTGGCTGGGGGGGATCATTTGAAATCAAAGTGATAGTGTCCGGGTCTGGCTTGTATGCGGGCAAATTGTTGTGCGCACTCATACCACCTGGGGTTGATCCCAGTGCTGTGGACCAGCCTGGGGCCTTCCCCCACGCACTGGTGGATGCACGCACCACTGAGGGTGTTACCTTCACCCTTGGGGATGTCAGGGCAGTGGACTATCATGAGACGGGGGCTGGTGGGACCATTGCATGTTTGGCACTTTACGTGTACCAACCACTAATCAACCCCTTTGAAACAGCCTTATCGACAGCCATGGTGACGATTGAGACCCGCCCTGGCCCAGACTTTGGGTTCACCCTGCTCAAGCCCCCAAACCAAACCATGGAGGTAGGACTGGACCCTAGGTCGCTCCTGCCCCGCACTGCAAGAACACTGCGGGGGAACAGGTTTGGTAGGCCCATTAGATCTGTGATTATAGTGGGTCTGGCACAACAAATCAACAGGCATTTTTCAGCAGAGGGCACCACACTTGGTTGGTCCACGGCCCCAATTGGCCCCTGTGTGGGCCGTGTCAACACAAAGTACACTGGTAACGCGGGCAAGGTGGTGGCCCAGCTGCTACCCTTGAGCAACGGGCCCCTTTACCCAAACATCATCAACCACTACCCAGATGTGGCTGCATCAATAATGCTCAGTGGAGGGTCTGGCATAACTGATGACATGACGTGTGGGGGAGGACCTATGGTGCTTTTCAACGATGTGGGTGATGTGGTGGAGAACGTCTCCTACCAAATGAGGTTCATAGCCTCACAGGCCACATCCCAAAATACCACACTCATCGACAAGATCAATGCAACATCAATGTCAGTGGTCAGTTTTGACAACTCCCGGAATGACTTTCCCCAAGCAAACGACAATGTGGGCATTGAGTTAACCTACACTTGTGGCTCCACACCAATTAACGGGAACGTCACCCAGTTCATGGACCGCCAATACACCTTTGGCGCGCAGGGGCCCAATAATATCATGCTTTGGGTGGAATCTGTGCTTGGCACGCACACTGGCAACAACAGAGTTTATAGCTCGCAACCAGACACCGTGTCTGCCGCACTGCAGGGCCAGCCCTACAACATACCAGAGGGGTACATGGCCGTGTGGAACGTCAACGCGGACAGTGCTGACTTTCAGATAGGGCTGAGACGCGATGGCTTCTTTATCACCAATGGAGCCATTGGCACAGAAATGGTCATTTCAGATGACACCACCTTCACCTTCAACGGCATGTACACCCTCACCACCCCCCTCATTGGACCAAGTGGGACATCAGGAAGGTCCATTCACGGCGCACGATGAGTTGGGTTGCAGGCGCAATGCAGGGGGCTGGCCTGCTGGGTGACCTTGCGGGCACAATTGGACAGATAGTGCTGTATAATAAGCAGTTGAATGTTACCAAATCTTTTAATCAGGCTCAGATTGAATTAGCTAAAGATCAATTGAAGCAGAATGTGGAATTGGCCAATAAGTATTATGAGTTTAATGAAAAATTGCCTGTTAATCAGTATAACAGTGCTGTGAGTGCTGGCTTTGACCCGGTTTCTGCACGTCAATTGGCTGGGTCGCGCGAGGTGCGTTACCTTGGTGGGCAACAAACACCGCTGCTCCACCAAGGGCAAATGCAACAAATGATGTTCTCATCAAAGCACCTCATGCAGGCACAAAACGTGCTTGGCACGTTTTCCCGTGGAACACCGGGTGTCACCCCCAGTAAGGGGTTACCCAAGCCACAGGGCGTCACATCCAGACCACTGGGCACTGCAGCCACTGTACCCGTGGCTGGCGCCACCACCACTCATAGCAGAGTGTGATTTTCTTTCCCTGGTTACGCGTACCGAGCCTTACACAGTGTGGCGGCTGTGGGGC

>YNAN [organism=Sapovirus GIII.] Sapovirus GIII gene, complete cdsGTGATCGTGATGGCTAATTGCCGTCCGTTGCCTATTGGGCATTTGCCCAACAGGATTTTTGGCACCCCACGACTCACCCCAGGGTGGGTGTGGGCGTGCACCAATGGAGCTGCATTCAAACTAGAGTGGCTCCAAGACCTGGTGGTCATACGACCCCCCGAGGTTTTTGTTGCGCAAGGGGTAGTGGATGACTTCTTCCGCCCCAAGCGCGTTTTGCAGGGTGACCCGCAACTCATTGCACAGGTTTTGCTGGGCGATGCCAACGGGCCACTCGTTGGCCCTGTGTCCATGCAGCAGCTAACATCGCTGCTGCATGAGGTGTCGCAAGCCCTTAATGACCACAAGCACCCACTAGCTGGCAGATACACCAGAGCCTCGTTGCAGAGGTACGCAGACACACTATCCAATTACATACCCCTTGTTGACGTTCTGACAGGACCTAAAGATCTCACCCCGCGGGATGTTCTTGAGCAACTCGCAGCGGGACGCGAGTGGGAATGTGTACCGGACAGTGCCCTTAAGAAGGTTTTCAGGGACATGTGGCAGTACATCTGTGAGGGGTGTGACAGCGTGTACATAAAGCTGCAAGATGTAAAACGCAAGATGCCACACATTGACACCACTGTGCTCAAGCAGTTTTTCATCACACTCACAGACACCATATCCATGGCAACAGCCTTGGACACCAAGACGTGGCTTGCACACATATTGGGTTGGCTCAAACCCACTTGCCTGGTGATGATCATGCAACAACATGTCAACTCTCCCCAGGGATGGGCCGCCACGCTAACCGCTCTAGCCGAGTTGTATTGTGGTATCATGCCATTGACAGAAACTCTTGGCTCTATTGCCAGTTGGGTGACTGACAAGTTTGCTGACATGGCGACCAACACCTGGAGCAAGTTCAAGACCTGGTGGGAAAGCCTGTACACACCACAAGCTGGCAATGACCTCATCATCTTGGGGGGTGTAGTTGGGCTGGTGTACTTTATGGTGTTTGGTGACGCGCCCACCCAAATGTTCACCAAGAAATTGATGAAGGTGTGTGGCTTTATCACCTCTACAGTGGCTGCAATCAAAGCGGCCATGTGGATAGTGGATTACTTCAAACAGCGTGAACATGAACACCAAGTTCGTGTCACGCTGGCTAGGTGGGCTGCACTGCAAGAGGTGATTAAACAGAACAGGTGTGCTGGCCTGTCTGAGGTGACCAAATTGAAAGAGTGCTGTGAAGTGTTGTTGAATGAGGTCACCGAGCTAATGTACAAACTCGGCGCCTCTCCTTTGGCAGGACTAATACGCAGCACCTCTGACGTCATACAAACTACCATCAATGACTTGGCTCAACTGATGGCTTATGACACCCAGCGCAAACCACCCGCCATGATAGTGTTTGGTGGTCCTCCGGGCATAGGCAAGACCAGGCTAGTTGAAGCTCTAGCAAGACAGTTGGGAGAAGTCAGCCATTTCACAATGACCGTCGACCACTACGACACCTACACGGGCAACACTGTTGCAATCTGGGATGAATTTGACGTGGATTCAAAACAAGCCTTCATTGAGGCCACCATTGGCATTGTCAACTGTGCCCCTTACCCCTTGAACTGTGATCGGCCGGAGGCGAAGGGTAGGGTGTTTACCTCCCAGTACATTCTGGCTACCACCAATTGCCCAACCCCAGTCATGCCCGACCACCCAAGGGCCATGGCCTTTTGGCGCCGCATAACATTTATTGATGTGACAGCCCCAACAATTGAACAGTGGTTGGTGGACAACCCGGGCCGCAAGGCTCCTACCTCCCTCTTCAAAGATGACTTCTCTCACCTGCAGTGTTCAGTGCGTGGTTATACTGCTTATGATGAAAAAGGCAACACACTGAGTGGCAAAGTAGCCAGGGCCAAGTATGTGTCAGTGAACAACCTTCTCGACTTGATCAAAGAAAAGTACAACAGTGAGGCCACTGACATCAAACACTTGTGGTTCACTGTCCCACAGGCTATACACAAGCAAGCCCGTGACACCATACTGGGCTGGCTGCGGTTCCACTCTTACCCAAACACAGTGGCAGACAACATACCATTGCCCGAGGTCAGGGACCCCACGTGCTTTGGGTACGTGGTCATTTCTGACGTGGATCCGCCACGGCATGTGAGCGACTACGTTGCCCACATTGAGGTGGAATCCATACTACGCACTGATATTATGGACCTGCTGAGGGAAGGTGGTGGCGGCTTGTTTAGGGCCCTAAAGGTGAAGAGCGCTCCTCGCAGCAGTGTTATCAACAAGGTCACGATGCAAGTGCACCACACCACCTTGCAAGTGCTCACCAGCCAGGAACCAAACCCACCAAATTTGCCACGGCCCAGGCGCTTAGTGTTTGTAGAATCACCCATTGACATCATCAGCGCCTTGCGGCACCATGTCGGTTTTTGCACCATCCCCGGCATTGTCAAGATGGTCACATCTGGGGTGGGTTTGGGTGTCGAAAACCTTGGCAATTTTCTGCAATCCATTGCTGGCAATGTCCGGTTTCCCCTGCAATCTGAGTGTTCACTGCTCAGGACACCCGGTGGTGATGTCTTGTTTTACACCTCAGGGCAGGCTGCCGTTTGGGCCACACCTGCGCGCTTCCCGATTGTCACCCCAGGTGAGGCGTCAGTTGGCAAGGATGTTTGTGCAGAGTCCTCCTGGTGGGACATTCTCAAAGCACTTTTCAGCACACTTGTGGTGGCTTTTGGGCCAATAGCCACACTGGTGTTGACAGCACACAACCTGGCCTATCTAAACGCCCGTGAAGGCACACTCAGCGAGGCAAAAGGGAAAAACAAGCGAGGGCGTGGCGCACGCAAGGCCATTGCCCTCAGGGACGATGAGTACGACGAGTGGCAGGACATCATACGTGACTGGCGCAAAGAAATGACCGTCCAACAATTCCTGGATCTCAAAGAGCGTGCCCTGTCCGGTGCCTCTGACCCTGACTCGCAGAGGTACAATGCCTGGCTTGAACTGCGTGCGAAAAGATTGAGTGCTGGGGCCTACCAGCACGCGGTTGTGGACATCATTGGGAAATCAGGCCACAGGCGTGAAGTGATCCGCACACAGGTGATGCGAGCCCCTCGTGAACCAAAAGCAGACACCTACGATAGTGAGGGGCGTGGTTACGTGGTGCCCATGACCGCCCAGGAAAAACATACAGGCTGGGCGGTGCACATTGGGAATGGCAGGCTGGTGACTTGCACACACGTGGCCAACATGTGTGATCGTGTGGCTGGGGTTGAGTTTAAGATCACTGAAACAGACCGTGATACATGCATCATCACCGCTCCACTCGGCCACTTGCCCAGTGTTGCCCTTGGGGATGGACCACCAGCCTTCTACACCACCAACTTCCACCCCATCCGTGTGTTGGACGAGGGCAGCTGGGACACCACAACCACCCGCGTCACCAGTTGGCGTGTCATTATCAATAACGGGACCACGACTGCCCCTGGTGATTGTGGCCAACCGTACTTGAATGCACGCCGGCAGCTTGTGGGGGTGCATGCGGCAACAAGCACATGTGGGGTCAAGAAACTCGTTTCCAGGGTGCAAACCAAAAGAGCATCAAAAGCCACATTTCCTTGGAAGGGTTTGCCTGTCACCAACATGCCTGATGCTGGTGGCCTGCCCACTGGCACGCGGTACCACCGCTCCACTGCGTGGCCCAAACAATTGCCAGAGGAAACGCATGCCCCTGCCCCTTACGGTGTGAACGACCCCCGGTACCCTTTTTCCCAGCACCAAATGATTGCCAACAACCTACAACCCTACACCAAGACCCCAACGCACTTGGACCAGACCCTGTTGCAGCGCGCTGTGCAACACACAAAGGGCTATCTGGATCAAGTCATTGGAACACACAGGTCCGCGAACCTCACCTATGCAGCAGCAGTGGACTCAATGGCACATGACACGGCATGTGGGCCGAATCTGCCTGGCCGCAAGAAGGATTACATGACTGACCAGGGGGAGCCCATTGGGCCACTCAAGCAGACGTTGGAAGAGGCTTGGGACATGGCACATCGAGGTGTCCCGCGCAAACACGAGTACAAGCTCGCGCTCAAGGATGAACTCCGGCCCATAGAAAAGAATGACCAGGGCAAGAGGCGCCTCCTTTGGGGTTGTGATGCCGGGGTGTCGTTGGTGGCCAATGCTGTGTTCAAGCCCGTGGCCGAGCGCCTGGTGGACACTGTGCCCATGCACCCAGTGGCAGTTGGCATATGTATGGACAGTCCTCAAATTGAGCAGATGAACCAGGCTCTAACAGGCAGGGTGCTGTACTGTTTGGATTACAGCAAATGGGACTCCACGCAAAACCCAGCTGTGACGAGCGCCAGTGTTGACATCTTGGCCTCTTACGCTGAGGACACACCACTCACCTCAGCAGCTATAGCCACACTGTGCTCACCAGCCATCGGCCGGCTTGATGACATTGGTTTGACTGTCACAACAGGACTCCCCTCTGGCATGCCATTCACCAGTGTCATAAACTCTGTCAACCACATGATATACTTTGCCATGGCCGTGCTTGAAGCTTACGAGGAGTTCAAGGTGCCCTACATGGGGAATATTTTTGACAACGAGACAATTTACACTTATGGGGATGATTGTGTGTACGGTCTGACACCGGCAACAGCTTCCATCATGCCGGTGGTGGTCAAAAACTTGACCAGCTACGGATTGGTGCCAACTGCTGCAGACAAATCCCAAACTATTGGACCCACTGACACCCCAGTGTTTCTCAAGCGCACCTTTTCTCAAACACCCTTCGGGCTGCGTGCACTGCTTGACGAGACAAGTCTAGCGCGGCAGTGCTACTGGGTCAAAGCCAATCGCACCACAGACCTTTTTGAACCAGCGGCCGTCGATGTGGAAATCAGGAGAAACCAACTGGAGGTCATGCTCGCATATGCCAGTCAACATCCAAGGGCAGTGTTTGACAAGCTCGCAAACATGGTTGAGGTGACTGCACATGCTGAGGGTTACCAAGTTGTAAATGTTGATTGGGCCAACGCAGTGGCAACGTACAACGCATGGTACGGTGGCACTGAAGGTGGCCATGCTCCCACCAGTGAAGATGAAGAGCCAGAAGTGTTCGTGATGGAGGCACCTGCCCCAACCCGTCCGGTTGCCAGCAACCCAGAGGGCACTCAAACCAGTAATGAGTCTAGACCAGTCCAGCCAGCTGGGCCCATGCCCGTGGCCACAGCCCAGGCCTTAGAAATGGCTGTCGCCACAGGGCAAATCAATGATACCATCCCCAGTGTGGTCAGAGAAACCTTTAGCACTTACACCAATGTCACCTGGACCACACGCCAGCCCACGGGGACCTTGCTTGCCCGTATGTCCCTCGGGCCGAGTCTGAACCCCTACACGCTCCACTTGTCTGCCATGTGGGCCGGCTGGGGTGGATCATTTGAAGTCAAAGTGGTGATATCAGGGTCTGGCTTGTATGCAGGCAAATTGCTGTGTGCGCTCATACCACCTGGGGTTGACCCCAGTGGTGTTGACCAACCCGGGGCGTTCCCGCACGCGCTTGTGGATGCGCGCACCACTGAGGGCGTCACCTTCACCTTGGGGGATGTTAGGGCGGTGGATTACCACGAAACAGGCGCTGGTGGGAACGTGGCATGCTTGGCACTTTATGTGTATCAACCACTCATCAACCCTTTTGAAACCGCCATCTCGGCTGCCATGGTGACAATTGAGACCCGCCCTGGTCCAGACTTCGGATTCACCCTGCTCAAGCCTCCTAACCAAACCATGGAGGTGGGGTTTGATCCCAGGTCGCTTCTGCCCCGCACGGCAAGAACGCTTCGTGGGAACAGGTTTGGCAGACCTATCACAGCTGTAGTCATAGTAGGTTTGGCACAACAAATCAACAGACACTTTTCAGCTGATGGTACCACACTTGGTTGGTCCACGGCCCCTGTTGCCCCATGTGTGGCACGTGTCAATGGAAAGTACACCGGCACCAATGGCATGGCAGTGTTTCAACTTCAGCCCTTGAGCAATGGGCCCCTCTACCCCAACATCGTCAACCACTACCCAGATGTGGCCGCATCAACAATACTCAGTGACAGGACCACCATTGCTGACAACATGACATGTGGTGGGGGGCCCATGGTGGTTTTTGATGACCGAGGTGATGTGACTGAGACTGTGGCTTACCAAATGAGGTTCATAGCCTCACATGCCACTTCCCAAAACCCCACACTTGTTGACAAAATCAATGCAACAACAATGGCACTGGTCAGCTTTGGCAACTCACGGGCAGACTTGGCCAATCCCAACTACAACGTGGGTATTGACCTCACTTACACCTGTGGCGAAACACCAATCAATGGGAACGTCACCCCATTCATGGAACGCCAATACACATTTGGCCCACAGGGACCCAACAACATCATGCTCTGGGTGGAGTCTGTGCTCGGTACACACAGAGGAAACAATGCCGTGTATAGCTCACAGCCAGACACAGTGTCCGCCGCGCTGCAGGGTCAGCCCTACAACATACCGGAAGGGTTCATGGCTGTGTGGAACGTTAATGCAGACAGTGCTGATTTCCAGATAGGATTGAGGCGTGATGGCTTCTTTGTCACCAATGGGGCCATTGGCACGCGCATGGTCATCCCTGAAGACACCACCTTTGATTTCAACGGCATGTACACCCTTTCCACCCCCCTCATTGGACCAAGTGGGACATCAGGAAGGTCCATTCATGGCCCACGATGAGTTGGGTTGCTGGCGCAATGCAAGGTGCTGGCCTGCTGGGTGATCTTGCAGGCACGATTGGACAAATAGTGTTGTACAACAAGCAGTTGAATATCCAGAAATCTTTCAATCAGGCCCAGATGGAGTTAGCTAAAGAACAATTAAAACAAAATGAGAAGTTGGCTGACCAGTACTACAAGTTTAATGAAAATTTGCCTGTTAACCAGTACAATAGTGCAGTGAGTGCTGGTTTTGACCCGGTTTCTGCGCGACAGCTGGTCGGTTCACGTGAAGTGCGTTATCTTGGTGGGCAACAAACACCACTGCTCCACCAAGGACAGGCACAACAAATGATGTTTTCATCAAAACACCTTATGCAAGCACAACACGTGGTCGGCACCTTTTCCCGCGGAATACCTGGCGTCACCCCTAGCAAGGGGCTGCCAAGGCCACAGGGTATCACACCTAGACCACAGGGTGTTACAGCTAGTGTGCCGGTGGCTGGCGCTACCACCACTCACAGCAGAGTGTGATTTTCTTTCCCTGGTTACGCGTACCGGACCTTACACAGTGTGGCGGCTGTGGGGC

>YNLH [organism=Sapovirus GIII.] Sapovirus GIII gene, complete cdsGTGATCGTGATGGCTAATTGCCGTCCGTTGCCTATTGGGCATTTGCCCAACAGGATTTTTGGCACCCCACGACTCACCCCAGGGTGGGTGTGGGCGTGCACTAATGGAGCTGCATTCAAACTGGAGTGGCTCCAAGACCCGGTGGTCATACGACCCCCGGAAATTTTTGTTGCGCAGGGGGTGGTGGACGATTTCTTCCGCCCCAAGCGCGTTTTGCAGGGTGACCCACAACTCATCGCACAGGTTTTGCTGGGCGACGCCAACGGACCTCTCGTTGGCCCCGTGTCCATGCAGCAGCTAACATCACTGCTGCATGAGGTGTCGCAGGCCCTTAATGACCATGAGCACCCACTGGCTGGCAGATACACCAGAGCCTCGTTGCAAAGGTACGCAGACACACTGTCCAATTACATACCCCTTGTTGACATCCTGACAGGACCTAAAGATCTCACCCCACGAGATGTTCTTGAGCAACTCGCAGCAGGACGCGAATGGGAATGTGTACCAGACAGTGCCCTTAAGAAGGTGTTCAGGGACATGTGGCAGCACATCTGTGAGGGATGTGACAGTGTGTACATAAAGCTGCAAGATGTGAAACGCAAGATGCCACACATTGACACCACGGTGCTCAAGCAGTTTTTCATCACACTCACAGACACCATATCCATGGCAACAGCCTTGGACACCAAGACGTGGCTCGCACACATATTAGGTTGGCTCAAACCCACCTGCTTGGTGATGATCATGCAACAACATGTCAATTCTCCCCAGGGATGGGCTGCCACACTGACTGCTCTAGCCGAGTTGTATTATGGCATCATGCCATTGACAGAAACGCTCGGCTCCATTGCCAGTTGGGTAACCGACAAGTTTGCCGACATGGCAACCAACACCTGGAGTAAGTTCAAGACCTGGTGGGACAGCCTATACACACCACAGGCTGGTAATGACCTCATCATCTTAGGGGGTGTGGTTGGGCTGGTGTACTTTATGGTGTTTGGTGACGCGCCCACCCAAATGTTCACCAAAAAGTTGATGAAGGTGTGTGGTTTTATCACCTCCACAGTGGCTGCCATCAAAGCGGCCATGTGGATAGTGGATTACTTCAAACAGCGTGAACATGAGCACCAAGTTCGTATCACGCTGGCTAGGTGGGCTGCGTTGCAAGAAGTGATCAAGCAGAACAGGTGTGCTGGCCTGTCTGAGGTGACCAAATTGAAAGAGTGCTGTGAGGTGTTGTTGAACGAGGTCACCGAGCTGATGTACAAACTTGGCGCCTCTCCTTTGGCAGGACTGATACGCAGCACCTCTGATGTCATACAAACCACCATCAATGACCTGGCCCAACTGATGGCTTATGACACCCAGCGCAAACCACCTGCCATGATAGTGTTTGGTGGACCTCCAGGCATAGGCAAGACCAGGCTAGTGGAAGCTCTAGCAAGACAGTTGGGAGAAGTCAGTCATTTCACAATGACTGTTGACCACTACGACACCTACACTGGCAACACTGTTGCAATTTGGGATGAGTTTGACGTGGACTCAAAACAAGCCTTCATTGAGGCCACTATTGGTATTGTCAACTGTGCCCCTTATCCCCTGAATTGTGATCGGCCAGAGGCAAAGGGCAGGGTGTTCACCTCCCAGTACATCTTGGCCACCACCAATTGTCCAACCCCGGTCATGCCCGACCACCCAAGGGCTATGGCCTTCTGGCGCCGCATAACATTTATTGATGTGACAGCCCCAACAATTGAACAGTGGTTGGTGGACAACCCGGGCCGCAAGGCTCCTGCCTCCCTCTTCAAGGATGACTTTTCTCACCTGCAGTGTTCAGTGCGTGGTTACACCGCTTACGATGAAAAAGGTAACACACTGAGTGGCAAAGTGGCCAGGGCCAAGTATGTGTCAGTGAACAATCTCCTCGATTTGATCAAAGAAAAGTACAACAGTGAGGCCGCTGACATCAAACACTTGTGGTTCACTGTCCCACAGGCCATACACAAACAAGCCCGTGACATCATATTGGGCTGGCTGCGGTTCCATTCTTACCCGAACACAGTGGCAGACAACATACCACTGTCCGAGGTGAGGGACCCCACGTGCTTTGGCTACGTGGTTATTTCTGACGTGGACCCGCCACGACATGTGAGTGACTATGTTGCCCACATCGAGGTAGAATCGGTGTTACGCACTGACATCATGGACCTGCTGAGGGAAGGTGGTGGCGGCTTGTTCAGGGCCCTAAAGGTAAAGAGTGCCCCTCGCAACAGTGTTATCAACAAGGTCATGATGCAAGCGCACCACACAACCTTACAGGTGCTCACCAACCAGGAACCAAACCCACCAAACTTGCCACGTCCCAGGCGTTTGGTGTTTGTGGAATCACCCATTGACATCATCAGCGCATTACGGCATCATGTCGGTTTTTGCACCATCCCCGGCATTGTCAAGATGGTCACATCTGGGGTGGGTCTGGGTGTTGAAAACCTTGGAAATTTTCTGCAATCCATCGCTGGCAATGTCCGGTTTCCCTTGCAATCTGAGTGTTCACTGCTCAGGACGCCCGGTGGTGACGTCCTGTTTTACACCTCAGGGCAGGCTGCCGTTTGGGCCACACCGGCGCGCTTCCCAATTGTCACCCCAGGTGAGGCGTCAGTTGGCAAGGATGTTTGTGCAGAGTCTTCCTGGTGGGACATTCTCAAAGCACTTTTCAGCACACTTGTGGTGGCATTTGGCCCGATAGCCACACTGGTGTTGACAGCACACAACTTGGCCTATCTGAACGCCCGTGAAGGCACACTTAGCGAGGCAAAGGGGAAAAACAAACGAGGGCGTGGTGCACGCAAGGCCATCGcCCTCAGGGATGATGAGTATGACGAGTGGCAGGACATCATACGTGATTGGCGCAAGGAAATGACTGTCCAACAGTTCCTGGATCTCAAAGAGCGCGCCCTGTCCGGTGCCTCCGACCCCGACTCGCAGAGGTACAATGCCTGGCTTGAACTGCGTGCGAAGAGATTGAGTGCCGGGGCCTACCAGCATGCGGTTGTGGACATCATTGGGAAATCAGGCCACAGGCGTGAAGTGATACGCACACAGGTGATGCGAGCCCCTCGTGAACCAAAAGCAGACACCTATGACAGTGAGGGGCGTGGTTACGTGGTGCCTATGACCGCTCAGGAAAAACATACAGGCTGGGCAGTGCATATTGGGAATGGCAGGTTGGTCACTTGCACACACGTGGCCAACATGTGCGATCGTGTGGCTGAGGTTGAGTTCAAGATCACTGAAACAGACCGCGACACATGCATCATCACCGCTCCGCTCGGCCACTTGCCGAGTGTCGCCCTTGGGGATGGACCACCAGCCTTCTACACCACTAACTTCCACCCCATCCGTGTATTGGATGAGGGCAGTTGGGACACTACAACCACCCGCGTCACCGGCTGGCGCGTCATCATCAATAACGGGACCACAACTGCCCCTGGTGATCGTGGCCAACCGTACTTGAATGCGCGCCGACAGCTTGTAGGAGTGCATGCTGCAACAAGCACATGTGGGGTCAAGAAACTTGTTTCGAGGGTGCAAACCAAAAGAGCATCAAAAGCCACATTCCCCTGGAAAGGTTTACCTGTCACCAACATGCCTGATGCTGGTGGCCTGCCCACTGGCACGCGGTACCACCGCTCAACCGCATGGCCCAAGCAGCTGCCAGAGGAAACGCATGCCCCTGCCCCTTATGGTGTGAATGATCCCCGGTACCCTTTTTCCCAACACCAAATGATTGCCAACAATTTACAACCTTACACCAAAACTCCAATCGCGCTAGACCAGACCCTACTGCAACGCGCTGTCAAGCACACAAAGGGGTACCTGGATCAAGTCATTGGAACACACAGGTCTCCAAATCTCACCTACGCAGCAGCAGTGGACTCGATGGCTCACGACACAGCGTGTGGGCCGAATCTACCTGGCCGTAAGAAGGACTATATGACAGACCAGGGGGAACCCATTGGGCCACTCAAGCAAATGTTAGAGGAGGCCTGGGACATGGCACACCGAGGTGTCCCACGCAAGCATGAGTACAAACTTGCGCTCAAGGATGAACTCCGGCCCATAGAAAAGAATGACCAGGGCAAGAGGCGCCTCCTTTGGGGCTGTGATGCTGGGGTGTCATTGGTGGCCAATGCCGTGTTCAAGCCTGTGGCCGAGCGCCTGGTGGACACTGTACCCATGCACCCGGTGGCTGTTGGCATATGCATGGACAGTCCTCAAATCGAACAGATGAACCAGGCCTTGACTGGTAGGGTGTTGTACTGTCTGGATTACAGCAAGTGGGATTCCACGCAAAACCCGGCTGTGACGAGTGCCAGCGTTGACATCTTGGCCTCTTATGCCGAGGATACACCACTCACCTCAGCAGCCATAGCCACACTGTGCTCACCAGCCATTGGCCGGCTTGACGACATTGGTTTGGCTGTTACAACGGGACTCCCTTCTGGCATGCCGTTCACCAGTGTCATAAACTCTGTCAACCACATGATATACTTTGCCATGGCCGTGCTTGAAGCTTATGAGGAGTTCAAGGTGCCCTACATGGGAAACATTTTTGACAATGAGACTGTCTACACATACGGGGATGATTGTGTGTACGGCCTGACACCGGCAACAGCTTCCATCATGCCGGTGGTGGTTAAAAACTTGACCAGTTACGGGCTGGTTCCAACTGCTGCAGACAAATCCCAAACTATTGAACCCACTGACACGCCAGTGTTTCTCAAGCGCACCTTTTCTCAGACACCCTTTGGGCTACGTGCGCTGCTTGACGAAACAAGTCTAGCGCGGCAGTGTTACTGGGTCAAAGCCAACCGCACTACAGATCTCTTTGAACCAGCGGCCGTTGATGTGGAAATCAGGAAAAACCAACTGGAGGTCATGCTCGCATATGCCAGCCAACATTCAAGGGCAGTGTTTAACAAGCTTGCAAACATGGTTGAGGTAACTGCACATGCTGAGGGTTACCAAGTTGTAAATGTTGATTGGGCCAACGCAGTGGCAACGTACAACGCATGGTACGGTGGCACTGAAGGTGGCCGTGCTCCCACCAGTGAAGATGATGAGCCAGAAGTGTTCGTGATGGAGGCGCCTGCCCCAACCCGTCCGATCACGAGCAACCCAGAAGGCACTGAGACCAGTGATGAGTCCAGGCCAGTTCAGCCGCCTGGGCCTGTGCCCGTGGCCGCAGCACAGGCTCTGGAGATGGCCGTTGCCACAGGGCAAATCAATGACACCATCCCCAGTGTGGTCAGGCAAACATTCAGTACTTACACCAATGTCACCTGGACCACGCGTCAACCCGCAGGAACCCTACTCGCCCGTATGTCCCTAGGGCCAGGATTAAACCCTTACACACTCCATTTGTCTGCTATGTGGGCCGGTTGGGGTGGGTCATTTGACATCAAGGTGGTAATATCAGGGTCTGGCATATATGCGGGCAAACTGTTGTGTGCACTAATACCACCTGGGGTTGACCCTAGCACTGTGGACCAACCCGGAGCCTTTCCCCACGCGCTTGTGGATGCGCGCACCACTGACGGTGTTTCATTTAACTTGGGGGATGTTAGGGCGGTGGACTACCATGAAACAGGAGCTGGTGGAAACGTAGCATGCCTGGCACTCTACGTGTACCAGCCACTCATTAACCCCTTTGAAACCACCATATCTGCTGCCATGGTGACAGTCGAGACCCGTCCTGGCGCAGACTTTGGGTTCACCCTGCTCAAGCCTCCAAACCAAACCATGGAGGTAGGACTTGACCCCAGGTCGCTCCTGCCCCGCACTGCAAGAACACTGCGGGGGAACAGGTTTGGCAGGCCCATCAGATCTGTGATCATAGTGGGTTTGGCACAACAAATCAACAGGCATTTTTCCGCAGAGGGTACCACACTTGGTTGGTCCACGGCCCCAATTGGCCCCTGTGTAGGCCGCATTAACACCAAGTACACTGGTAATGCGGGCAAGGTAGTAGCACAACTGTTACCTTTGAGCAATGGGCCCCTTTACCCAAATATCATCAACCACTACCCAGACGTGGCTGCATCAACAATGCTCAGCGGAGGGTCTAGCATAACTACTGACATGACGTGTGGGGGAGGGCCCATGGTGCTCTTCAACGATGTGGGTGATGTGGTGGAGACCGTCTCCTACCAAATGAGGTTCATAGCCTCACAGGCCACATCTCAAAGCACCACACTCATCGATAAGATCAATGCAACATCAATGTCAGTGGTCAGTTTTGACAACTCCCGGAATGACTTTCCCCAATCAAATGACAATGTGGGTATTGAGTTAACCTACACTTGTGGCAACACACCGATCAATGGGAATGTCACCCAGTTCATGGACCGCCAATACACCTTTGGCGCACAAGGGCCCAACAACATTATGCTTTGGGTGGAGTCTGTGCTTGGCACGCACACTGGCAACAACAGAGTCTACAGTTCACAACCAGATACCGTGTCTGCTGCATTGCAAGGCCAGCCCTACAACATACCAGAAGGGTACATGGCCGTGTGGAACGTCAACGCGGACAGTGCTGACTTTCAAATAGGGCTGAGGCGCGATGGCTTCTTCATCACCAATGGCCCCATTGGCACAGAAATGGCCATTTCAGATGACACCACCTTCACCTTCAATGGAATGTACACCCTAACCACCCCCCTCATTGGACCAAGTGGGACAACAGGAAGGTCCATTCACAGCTCACGATGAGTTGGGTTGCAGGCGCAATGCAGGGCGCTGGCCTGCTGGGTGACCTCGCAGGCACAATTGGACAAATAGTGTTGTACAATAAGCAGTTGAATATTACCAAATCTTTTAATCAGGCTCAGTTGGAGTTAGCCAAAGATCAAATGAAACAAAATCAACAGTTGGCTAACCAGTATTATGAATTTAATGCAAATTTGCCTGCCAATCAGTATAATAGTGCTGTGAGTGCTGGTTTTGACGCGGTTTCTGCACGTCAATTGGCTGGGTCGCACGAAGTGCGTTACTTTGGTGGGCAACAAACACCATTGCTCCACCAAGGGCAAATGCAACAAATGATGTTCTCGTCAAAACATCTTATGCAAGCCCAAAACGTGCTTGGCACCTTTTCTCGTGGGACACCTGGTGTCACCCCCAGTAAGGGGCTGCCCAAACCACAGGGCATCACGTCCAGACCGCTCAGGCCACAGGGTGTTTCAGCCACCATACCGGTGGCTGGAGCCACCACCACCAACAGCAGAGTGTGATTTTCTTTCCCTGGTTACGCGTACCGAACCTTACACAGTGTGGCGGCTGTGGGGC

>YNAN1 [organism=Sapovirus GIII.] Sapovirus GIII genes for VP1 capsid protein and VP2 minor capsid protein, complete cdsATGGAGGCGCCTGCCCCTACCAGTTCGGTTGCGAGCAACCCAGAGGGCACTCAGAATAGCAATGAGTCCAGACCAATCCAGCCAGCTGGACCCATGCCAGTGGCTGCGGCCCAGGCCCTAGAAATGGCTGTTGCCACAGGGCAGGTCAATGACACCATCCCTAGCGTGGTGAGAGACACTTTTAGCACCTTCACTAATGTCACCTGGACCACACGTCAACCTGCAGGGACTTTGCTAGCCCGAATGTCTTTGGGACCTGGCTTAAACCCCTACACACTCCACTTGTCAGCTATGTGGGCTGGCTGGGGTGGATCTTTTGAAATCAAGGTGGTGATATCAGGGTCTGGCATGTACGCGGGCAAGCTGCTATGCGCGCTTATGCCACCAGGGGTTGACCCCGGTGCCATAGAACAGCCTGGGGCCTTTCCGCACGCACTTGTTGATGCGCGCATCACTGACGGCGTCACCTTCACCCTTGGGGATGTGAGAGCGGTAGATTACCACGAGACTGGGGCTCCTGGCGCCGTTGCATGCTTGGCACTTTACGTGTACCAGCCACTCATCAACCCTTTCGAAACCACCTTATCGGCTGCCATGGTAACTGTTGAGACTCGCCCCGGCCCAGACTTTGGATTCACCCTGCTCAAGCCTCCAAACCAAACCATGGAGGTGGGACTTGACCCCAGGTCGCTCCTGCCCCGCACTGCAAGAACACTGCGGGGGAACAGATTTGGCAGGCCCATCAGGTCTGTGATCATAGTGGGTTTGGCACAGCAAATCAACAGGCATTTTTCAGCAGAGGGCACCACACTTGGTTGGTCCACGGCTCCAATTGGCCCCTGTGTGGGCCGCATCAACACAAAGTACACTGGCAATGCAGGTAAGGTGGTGGCCCAACTGCTGCCTCTGAGTAATGGGCCGCTTTACCCAAACATCATCAACCACTACCCAGATGTGGCTGCATCAACAATGCTCAGTGGAGGGTCTAGCATAACTGCTGACATGACGTGTGGGGGAGGACCTATGGTGCTTTTCAACGATGTGGGCGATGTAGTGGAGACCGTCTCCTACCAAATGAGGTTCATAGCCTCACAGGCCACTTCTCAAAACACCACACTCGTCGACAAGATCAATGCAACATCAATGTCAGTGGTCAGTTTTGACAACTCCCGGAATGACTCCCCTCAGTCAAACGACAATGTGGGTATTGAGCTAACCTACACTTGTGGCAACACACCAATCAACGGGAATGTCACCCAGTTCATGGACCGCCAATACACCTTTGGCGCACAAGGGCCCAACAACATTATGCTTTGGGTGGAGTCTGTGCTTGGCACGCGCACTGGCAACAACAGAGTTTACAGCTCACAACCAGACACCGTGTCTGCTGCGCTGCAAGGCCAGCCCTACAACATACCAGAGGGGTACATGGCTGTGTGGAACGTCAACGCGGACAGTGCTGACTTTCAAATAGGGCTGAGGCGCGATGGCTTCTTTATCACCAATGGAGCCATTGGCACAGAAATGGCCATTTCAGATGACACCACCTTCACGTTCAACGGCATGTACACCCTTACTACCCCCCTCATTGGACCAAGTGGGACAACAGGAAGGTCCATTCATGGCGCACGATGAGTTGGGTCGCAGGTGCAATGCAGGGCGCTGGCCTGGCAGGCGATCTTGTCGGCACAGTCGGACAAATTGTATTATACAATAAGCAGTTGAACATTCAAAAATCTTTCAACCAGGCCCAGATGGAGTTGGCTAAAGAACAATTGAAGCAAAATCAACACATGGCCAACCAGTTTTATAAATTTAATTCAGATTTACCTGCTAATCAGTACAATAGTGCTGTGAGTGCCGGATTTGATTCAGTCTCTGCACGCCAATTGGCTGGGTCCCGCGAGGTGCGCTACTTCGGTGGACAGCAAACACCTATGCTCCACCAAGGGCAGATGCACCAAATGATGTTTTCATCAAAACACTTGATGCAGGCACAAAATGTGCTCGGCACTTTTTCCCGTGGGACGCCCGGTGTGACCCCTAGCAAAGGGTTGCCCAAACCGCAGGGCATCAATCCCAGACCACAGAGCAGTACGGCTACCCTGCCTGTGTCTGGAAACACCACCACTCACAGCAAGGTGTGATTTTCTTTCCCTGGTTACGCGTACCGAACCTTACACAGTGTGGCGGCT

>YNXSBN1 [organism=Sapovirus GIII.] Sapovirus GIII genes for VP1 capsid protein and VP2 minor capsid protein, complete cdsATGGAGGCACCTGCCCCAACTCGTTCGGTGGCGAGTAACCCAGAAGGTACTCAAACTAGCAATGAATCCAGACCAGTTCAGCCAGCCGGGCCCATGCCCGTGGCCACTGCTCAGGCCCTTGAGATGGCTGTTGCCACTGGGCAAGTTAATGACACCATCCCCAGTGTGGTCAGAGAAACTTTTAGCACCTACACCAATGTCACTTGGACCACACGCCAGCCTGCAGGAACCCTGCTCGCCCGGATGTCCCTAGGGCCAGGTTTGAACCCCTACACGCTCCACCTGTCTGCTATGTGGGCTGGTTGGGGAGGGTCATTTGAAATCAAGGTGATAATATCAGGGTCTGGTTTGTATGCGGGCAAATTGCTGTGTGCACTCATACCACCTGGGGTTGACCCCAGCGCTGTGGATCAGCCTGGGGCCTTCCCCCATGCACTTGTGGATGCACGCATCACTGAGGGCGTCACCTTCACCCTCGGGGACGTGAGGGCAGTGGATTACCACGAAACAGGTGCCGGTGGGACCATTGCATGTTTGGCACTTTATGTATATCAACCGCTCATCAACCCTTTTGAAACTGCCTTGTCGGCTGCCATGGTGACGATTGAAACCCGGCCTGGCCCAGACTTTGGATTCACCCTGCTCAAGCCTCCAAACCAAACCATGGAGGCGGGGCTTGACCCTAGGTCGCTCCTGCCCCGCACGGCAAGAACACTACGGGGAAACAGGTTTGGTAGACCTATCACAGCCGTGGTTATAGTTGGCATGGCACACCAAATTAATAGGCATTTTTCCGCTGAGGGCACCACGCTTGGGTGGTCCACAGCCCCAATTGGTCCTTGTGTGGGTCGCATCAACTCCAAGTACACCAACACCACCGGCCCAGCTGTGCTCTCGCTGCAACCCCTGAGCAATGGGCCCCTCTACCCCAACATCATCAACCACTACCCAGATGTGGCTGCGTCAAGGGCGTTCAACACCAGCACCAGCCTAACTGACAGCACCACGTGTGGGGGGGGGCCTATGGTGGTCTTTAATGATGTGGGTGATGTGGTCGAAAATGTGACCTACCAGATGAGGTTCATAGCCTCCCAAGCCACCTCCCAGACACCTACACTTGTTGATTACATTAATGCAACATCAATGGCAGTGTGCAGTTTCGGCAACTCTCGAGGAGACTTTGGCTCAGGCCAACTTAATGTGGGTGTTGAGTTGACCTACACCTGTGGCAACACAGCGATTAATGAAAAAGTCACCACGTTCATGGATCGCCAATACACATTTGGTGCACAGGGGCCCAACAACATCATGCTCTGGGTTGAAACTGTGCTTGGCACGCACACAGGCAACAACTCTGTGTATAGTTCACAACCCGACACTGTTGCTGCCGCACTGCAGGGTCAGCCCTACAACATACCAGATGGGTACATGGCTGTGTGGAACGTCAATGCAGACAGTGCCGACTTCCAGATAGGTCTGAGGCGCGATGGCTTCTTTATCACCAGCGGAGCCATTGGCACGCGCATGACCATTTCAGAAGACACCACTTTCACCTACGCTGGTATTTTCACCCTCACTACCCCCCTTATTGGACCAAGTGGGACGACAGGAAGGTCCCTTCACAGCTCACGATGAGTTGGGTTGCAGGCGCAATGCAGGGCGCTGGCCTGCTGGGAGACCTTGCAGGCACAATTGGACAAATAGTGTTGTATAACAAACAGTTGAATATTCAGAAAAATTTTAATCAGGCTCAGTTAGAGTTGGCCAAAGAACAATTGAAACAAAATCAAAATTTGGCTAACCAGTATTATGAGTTTAATGCCAACTTACCTGTTAATCAGTACAATAGTGCTGTGAGTGCTGGTTTTGATGCGGTTTCCGCACGTCAAATGGCTGGGTCGCACGAAGTGCGCTATTTTGGTGGGCAACAAACACCATTACTCCACCAAGGGCAAATGCAACAAATGATGTTTTCATCAAAACATCTCATGCAAGCGCAGAACGTGCTTGGCACCTTTTCTCGTGGAACACCTGGTGTCACGCCCAGCAAGGGACTGCCCAAGCCACAGGGCATTACGCCCAGACCACTCAGACCACAGGGCGTTTCAGCCACCATACCTGTGGCTGGAGCCACCACCACTAACAGCAGAGTGTGATTTTCTTTCCCTGGTTACGCGTACCGAACCTTACACAGTGTGGCGGCT

>YNBS1 [organism=Sapovirus GIII.] Sapovirus GIII genes for VP1 capsid protein and VP2 minor capsid protein, complete cdsATGGAGGCACCTGCCCCAACCCGTCCGGCTGCGAGCAACCCAGAGGGCACTCAAACTAGCAATGAGTCTAGGCCAGTTCAGCCAGCCGGGCCCATGCCCGTGGCTGCGGCCCAGGCCTTGGAAATGGCTGTCGCTACAGGACAAATCAATGACACCATCCCTAGTGTGGTGAGGGAAACTTTTAGCACCTACACCAATGTCACCTGGACCACACGTCAGCCTGCAGGAACCTTACTTACCCGGATGACCCTGGGGCCAGGTCTGAACCCTTACACACTCCACCAGTCTGCCATGTGGGCTGGCTGGGGGGGATCATTTGAAATCAAAGTGATAGTGTCCGGGTCCGGCTTGTATGCGGGCAAATTGTTGTGCGCACTCATACCACCTGGGGTTGATCCCAGTGCTGTGGACCAGCCTGGGGCCTTCCCCCACGCACTGGTGGATGCACGCACCACTGAGGGTGTTACCTTCACCCTTGGGGATGTCAGGGCAGTGGACTATCATGAAACGGGGGCTGGTGGGACCATTGCATGTTTGGCACTTTACGTGTACCAACCACTCATCAACCCCTTTGAAACAGCCTTATCGGCAGCCATGGTGACGATTGAGACCCGCCCTGGCCCAGACTTTGGGTTCACCCTGCTCAAGCCTCCAAACCAGACCATGGAGGTGGGACTTGACCCCAGGTCACTCCTGCCCCGCACGGCAAGAACACTGCGGGGAAACAGGTTTGGCAGACCCATTACAGCCGTAGTCATAGTGGGCATGGCACACCAAATTAACAGGCACTTCTCAGCCGAGGGCACCACGCTTGGGTGGTCCACAGCACCAATAGGTCCTTGTGTGGGTCGCATTAATTCCAGGTACACCAATGCCGGTGGCTTAGCCGTGCTCTCACTACTACCCCTGAGCAATGGGCCCCTTTACCCCAACATCGTCAACCACTACCCAGATGTAGCTGCATCAAGGGCATTCAACACCAGCACCAGTCTAACCGCCAACACCACGTGTGGGGGAGGGCCCATGGTGGTCTTTAATGATGTGGGTGATGTGGTTGAAAGCTTGACCTACCAGATGAGATTCATAGCTTCTCAAGCCACTTCCCAAACACCTACACTTGTTGACTACATCAATGCAACATCAATGGCAGTGTGCAGTTTTGACAATTCCCGAGGAGACTTTGGCACAGGCCAACTCAACGTGGGTGTTGAATTGACTTACACCTGTGGCAACACAGCAATCAATGAAAAAGTCACCACGTTCATGGACCGCCAATACACATTTGGCGCACGGGGGTCTAACAACATCATGCTGTGGGTAGAGCGTGTACTCGGCACGCACACGGGCAACAATGCGGTGTACAGCTCGCAACCCGACACCGTGTCTGCCGCATTGCAGGGTCAGCCCTACAACATACCAGATGGGTACATGGCTGTGTGGAATGTCAATGCGGACAGTGCTGACTTCCAGATAGGCCTGAGGCGCGATGGCTTCTTTGTCACCAGTGGGGCCATTGGCACGCAAATGATCATCTCGGAGGATACCACTTTCACCTACGCTGGCATGTTCACCCTCACCACCCCCCTTATTGGACCAAGTGGGACGACAGGAAGGTCCATCCACAGCTCACGATGAGTTGGGTTGCAGGCGCAATGCAGGGCGCTGGCCTGCTAGGAGACCTTGCAGGCACAATTGGACAAATAGTGTTGTATAACAAACAGTTGAATATTCAGAAAAATTTTAATCAGGCTCAGTTAGAGTTGGCCAAAGAACAATTGAAACAAAATCAAAATTTGGCTAACCAGTATTATGAGTTTAATGCCAACTTACCTGTTAATCAGTACAATAGTGCTGTGAGTGCTGGTTTTGATGCGGTTTCCGCACGTCAAATGGCTGGGTCGCACGAAGTGCGCTATTTTGGTGGGCAACAAACACCATTACTCCACCAAGGGCAAATGCAGCAAATGATGTTTTCATCAAAACATCTCATGCAAGCGCAGAACGTGCTTGGCACCTTTTCTCGTGGAACACCTGGTGTCACGCCCAGCAAGGGACTGCCCAAGCCACAGGGCATTACGTCCAGACCACTCAGACCACAGGGCGTTTCAGCCACCATACCTGTGGCTGGAGCCACCACCACTAACAGCAGAGTGTGATTTTCTTTCCCTGGTTACGCGTACCGAACCTTACACAGTGTGGCGGCT

>YNDY1 [organism=Sapovirus GIII.] Sapovirus GIII genes for VP1 capsid protein and VP2 minor capsid protein, complete cdsATGGAGGCACCTGCCCCAACCCGTCCGACTGCGAGCAACCCAGAGGGTACTCAAACCAGTAATGAATCCAGACCAGTCCAGCCAGCTGGGCCCATGCCCGTGGCCGCGGCCCAAGCCTTGGAGATGGCTGTTGCCACAGGGCAGATCAATGACACCATCCCCAGTGTGGTCAGGGAAACCTTTAGCACTTACACCAACGTCACCTGGACCACACGCCAGCCTACGGGGACTTTGCTTGCTCGAATGTCTCTCGGGCCAAGTCTGAACCCCTACACGCTCCACTTGTCTGCCATGTGGGCTGGCTGGGGTGGATCATTTGAAGTCAAAGTGGTGATATCAGGGTCTGGCTTGTATGCAGGCAAATTGCTGTGCGCACTCATACCACCCGGGGTTGACCCCAGTGGCGTTGACCAACCCGGGGCGTTCCCACACGCGCTTGTGGATGCGCGCATCACTGAAGGTGTCACCTTTACCTTGGGGGATGTTAGGGCGGTGGATTACCACGAAACAGGCACTGGTGGTAATGTGGCATGCTTGGCACTTTATGTGTACCAACCACTCATCAACCCTTTCGAGACTGCCATCTCGGCTGCCATGGTGACAATTGAGACCCGCCCTGGCCCAGACTTTGGGTTCACCCTGCTCAAGCCTCCAAACCAAACCATGGAGGTGGGATTTGATCCCAGGTCGCTCCTGCCCCGCACGGCAAGAACGCTCCGGGGCAACAGGTTTGGTAGACCTATCACAGCTGTGGTCATAGTAGGTTTGGCACAACAAATCAACAGACATTTTTCTGCTGATGGCACCACACTTGGCTGGTCCACGGCCCCAGTTGCCCCATGTGTGGCACGTGTCAATGGAAAGTACACCGGCACCAACGGCATGGCAGTGTTTCAGCTCCAACCCTTAAGCAATGGGCCCCTTTACCCCAACATCGTCAATCACTACCCAGATGTGGCCGCATCAACAATACTTAGTGACAGGACCACCGTTGCTGACAACATGACATGTGGTGGGGGGCCTATGGTGGTTTTTGATGATCAAGGTGATGTAACTGAAACTGTGGCTTACCAAATGAGGTTCATAGCCTCACATGCCACCTCCCAAAACCCCACACTGGTTGACAAAATTAATGCAACAACAATGGCACTGTGCAGTTTTGGCAACTCACGGGCAGATCTTGGCCAGTCCCAGCTCAACGTGGGTATTGACCTAACTTACACCTGTGGCGAAACACCGATCAATGGGAACGTCACCCCATTCATGGACCGCCAATATACATTTGGCCCACAGGGACCCAACAACATCATGCTTTGGGTGGAGTCTGTGCTCGGCACACACAGAGGAAACAACGCCGTGTACAGTTCACAGCCAGACACTGTGTCTGCCGCGCTGCAGGGTCAGCCTTACAACATACCGGAAGGGTTCATGGCTGTGTGGAATGTCAATGCGGACAGTGCTGACTTCCAGATAGGGTTGAGGTGTGATGGCTTCTTTGTCACCAATGGGGCCATTGGCACGCGTATGCTCATCCCTGAGGACACCACCTTTGATTTCAACGGCATGTACACCCTTTCCACCCCCCTCATTGGACCAAGTGGGACATCAGGAAGGTCCATTCATGGCCCACGATGAGTTGGGTTGCTGGCGCAATGCAAGGTGCTGGCCTGCTGGGTGATCTTGCGGGCACGATTGGGCAAATTGTGTTGTACGACAAGCAGTTGAATATTCAGAAATCTTTTAATCAGGCCCAGATGGAGTTGGCTAGAGAACAATTGAAACAAAATGAAAAGTTAGCCAACCAGTATTACAAATTTAATGAAAATTTGCCTGTTAATCAGTACAACAGTGCAGTGAGTGCTGGTTTTGACCCGGTTTCCGCGCGTCAGCTGGCCGGTTCACGTGAAGTGCGTTATCTTGGTGGGCAACAAACACCACTGCTCCATCAAGGCCAGACACAACAAATGATGTTTTCCTCAAAACACCTGATGCAAGCACAACACGTGGTCGGCACCTTTTCTCGCGGGATACCTGGTGTCACTCCTAGTAAGGGGTTGCCAAGGCCACAGGGCATCACACCTAGACCACAGGGTGTTACAGCTAGTGTGCCCGTGGCTGGCGCCACCACCACCCACAGCAGAGTGTGATTTCTTTTCCCTGGTTACGCGTACCGGACCTTACACAGTGTGGCGGCT

>YNDY2 [organism=Sapovirus GIII.] Sapovirus GIII genes for VP1 capsid protein and VP2 minor capsid protein, complete cdsATGGAGGCACCTGCCCCAACCCGTCCGACTGCGAGCAACCCAGAGGGTACTCAAACCAGTAATGAATCCAGACCAGTCCAGCCAGCTGGGCCCATGCCCGTGGCCGCGGCCCAAGCCTTGGAGATGGCTGTTGCCACAGGGCAGATCAATGACACCATCCCCAGTGTGGTCAGGGAAACCTTTAGCACTTACACCAACGTCACCTGGACCACACGCCAGCCTACGGGGACTTTGCTTGCTCGAATGTCTCTCGGGCCAAGTCTGAACCCCTACACGCTCCACTTGTCTGCCATGTGGGCTGGCTGGGGTGGATCATTTGAAGTCAAAGTGGTGATATCAGGGTCTGGCTTGTATGCAGGCAAATTGCTGTGCGCACTCATACCACCCGGGGTTGACCCCAGTGGCGTTGACCAACCCGGGGCGTTCCCACACGCGCTTGTGGATGCGCGCATCACTGAAGGTGTCACCTTTACCTTGGGGGATGTTAGGGCGGTGGATTACCACGAAACAGGCACTGGTGGTAATGTGGCATGCTTGGCACTTTATGTGTACCAACCACTCATCAACCCTTTCGAGACTGCCATCTCGGCTGCCATGGTGACAATTGAGACCCGCCCTGGCCCAGACTTTGGGTTCACCCTGCTCAAGCCTCCAAACCAAACCATGGAGGTGGGATTTGATCCCAGGTCGCTCCTGCCCCGCACGGCAAGAACGCTCCGGGGCAACAGGTTTGGTAGACCTATCACAGCTGTGGTCATAGTAGGTTTGGCACAACAAATCAACAGACATTTTTCTGCTGATGGCACCACACTTGGCTGGTCCACGGCCCCAGTTGCCCCATGTGTGGCACGTGTCAATGGAAAGTACACCGGCACCAACGGCATGGCAGTGTTTCAGCTCCAACCCTTAAGCAATGGGCCCCTTTACCCCAACATCGTCAATCACTACCCAGATGTGGCCGCATCAACAATACTTAGTGACAGGACCACCGTTGCTGACAACATGACATGTGGTGGGGGGCCTATGGTGGTTTTTGATGATCAAGGTGATGTAACTGAAACTGTGGCTTACCAAATGAGGTTCATAGCCTCACATGCCACCTCCCAAAACCCCACACTGGTTGACAAAATTAATGCAACAACAATGGCACTGTGCAGTTTTGGCAACTCACGGGCAGATCTTGGCCAGTCCCAGCTCAACGTGGGTATTGACCTAACTTACACCTGTGGCGAAACACCGATCAATGGGAACGTCACCCCATTCATGGACCGCCAATATACATTTGGCCCACAGGGACCCAACAACATCATGCTTTGGGTGGAGTCTGTGCTCGGCACACACAGAGGAAACAACGCCGTGTACAGTTCACAGCCAGACACTGTGTCTGCCGCGCTGCAGGGTCAGCCTTACAACATACCGGAAGGGTTCATGGCTGTGTGGAATGTCAATGCGGACAGTGCTGACTTCCAGATAGGGTTGAGGCGTGATGGCTTCTTTGTCACCAATGGGGCCATTGGCACGCGTATGCTCATCCCTGAGGACACCACCTTTGATTTCAACGGCATGTACACCCTTTCCACCCCCCTCATTGGACCAAGTGGGACATCAGGAAGGTCCATTCATGGCCCACGATGAGTTGGGTTGCTGGCGCAATGCAAGGTGCTGGCCTGCTGGGTGATCTTGCGGGCACGATTGGGCAAATTGTGTTGTACGACAAGCAGTTGAATATTCAGAAATCTTTTAATCAGGCCCAGATGGAGTTGGCTAGAGAACAATTGAAACAAAATGAAAAGTTAGCCAACCAGTATTACAAATTTAATGAAAATTTGCCTGTTAATCAGTACGACAGTGCAGTGAGTGCTGGTTTTGACCCGGTTTCCGCGCGTCAGCTGGCCGGTTCACGTGAAGTGCGTTATCTTGGTGGGCAACAAACACCACTGCTCCATCAAGGCCAGACACAACAAATGATGTCTTCCTCAAAACACCTGATGCAAGCACAACACGTGGTCGGCACCTTTTCTCGCGGGATACCTGGTGTCACTCCTAGTAAGGGGTTGCCAAGGCCACAGGGCATCACACCTAGACCACAGGGTGTTACAGCTAGTGTGCCCGTGGCTGGCGCCACCACCACCCACAGCAGAGTGTGATTTCTTTTCCCTGGTTACGCGTACCGGACCTTACACAGTGTGGCGGCT

>YNDC1 [organism=Sapovirus GIII.] Sapovirus GIII genes for VP1 capsid protein and VP2 minor capsid protein, complete cdsATGGAGGCGCCTGCCCCAACCCGTCCGGCTGCGAGCAACCCAGAGGGTACCCAAACCAGTAATGAGTCCAGACCAGTCCAGCCAGCTGGGCCCATGCCCGTGGCGGCGGCCCAAGCCCTGGAAATGGCTGTCGCCACTGGGCAAATCAATGACACCATCCCCAGTGTGGTTAGAGAAACTTTCAGCACCTACACCAATGTTACCTGGACCACACGCCAGCCTGCAGGGACTATGCTTGCCCGTATGTCCCTAGGGCCTGGCCTGAATCCCTACACACTCCACCTGTCCGCTATGTGGGCTGGCTGGGGCGGGTCGTTTGAGGTGAAAGTCATCATATCAGGGTCTGGTATGTATGCAGGCAAGTTGCTGTGTGCGCTCATACCACCTGGGGTCAACCCTACCACTGTGGACCAACCTGGGGCTTTCCCACATGCACTCGTGGACGCGCGCATCACTGATGGTGTCACTTTCACCTTGGGTGACGTCAGAGCGGTTGATTACCACGAAACAGGCACTGGTGGCAACGTGGCACAACTGGCACTTTATGTGTACCAACCACTCATCAACCCCTTTGAAACCAACGTGTCGGCAGCCATGGTGACTGTTGAGACCCGCCCTGGTCCAGACTTCGGGTTCACCCTGCTCAAGCCTCCTAACCAGACCATGGACGTGGGGTTCGACCCTAGGTCACTCCTGCCCCGGACAGCAAGAACACTGCGGGGGAACAGGTTTGGCAAACCCATTAGAGCCGTGGTCATAGTGGGCACAGCACAACAAATCAACAGGCACTTTTCGGCCGAGGGCACCACACTTGGGTGGTCCACGGCCCCAGTCGGCCCCTGTGTGGGTCGCGTGAACAACAAGTACACTGGCACCACTGGCAAGGTTGTGCTCCAGCTTCTGCCCCTGGACAATGGTCCCCTTTACCCCAACATCATCAACCACTACCCAGACATAGCTGCATCAACAACAATCAATGACAGCAGCGCCATATCCGACACCATGATGTGTGGTGGAGGGCCCATGGTGCTCTTTGACAACAATGGTGATGTAAGAGAGAACGTGGCTTACCAGATGAGGTTCATAGCTTCACGGGCCACGTCCCAAAGTGCCACACTCATTGACCAAATCGATGCAACATCAATGTCAGTGTGTAGTTTTGGTAACTCCCGGGCCGACCTCAGTGAGACCGAAATCAATGTGGGCATTGAGATGACTTACACCTGTGGTAACAGGCCCATCGATGGGATAGTCACCACGTTTATGGGCCAACAATACACATTTGGATCACAGGGAGCCAACAACATCATGCTTTGGGTGGAAAGTGTGCTCAGCTCATACACTGGAATGAACTCTGTGTACAGTTCTCAACCAGATACTGTATCTGCTGCACTGCAGGGCCAGCCCTTCAATATACCAGAGGGGCACATGGCGGTGTGGAACGTGAGCGCAGACAGTGCTGACTTCCAGATAGGCTTGAGGCGCGATGGCTATTTTGTCACCAATGGTGCCATTGGCACGAGCATAGCCATCCCAGAAGACACCACCTTCACCTACAGCGGCGTCTACACCCTCACCACCCCCCTCATTGGACCAAGTGGGACATCAGGGAGGTCCATTCACAGCTCACGATGAGTTGGGTTGCAGGCGCAATGCAGGGCGCTGGCCTGCTGGGTGACCTCGCAGGCACAATTGGACAAATAGTGTTGTACAATAAGCAGTTGAATAACACTAAATCTTTTAATCAGGCTCAGTTAGAGTTAGCCAAAGATCAAATGAAACAAAATCAACAGTTGGCTAACCAGTATTATGAATTTAATGCAAATTTGCCTGCCAATCAGTATAATAGTGCTGTCAGTGCTGGTTTTGATGCGGTTTCTGCACGTCAATTGGCTGGGTCGCACGAAGTGCGCTACTTTGGGGGACAACAAACACCAATGCTCCACCAAGGGCAAATGCAACAAATGATGTTCTCGTCAAAACACCTTATGCAAGCGCAAAACGTGCTTGGCACCTTTTCTCGTGGGACACCTGGTGTCACCCCCGGCAAAGGGCTGCCCAAACCACAGGGCATCACGTCCAGACCGCTCAGACCACAGGGTGTTTCAGCCACCATACCTGTGGCTGGAGCCACCACCACCAATAGCAGAGTGTGATTTTCTTTCCCTGGTTACGCGTACCGAACCTTACACAGTGTGGCGGCT

>YNLJ1 [organism=Sapovirus GIII.] Sapovirus GIII genes for VP1 capsid protein and VP2 minor capsid protein, complete cdsATGGAGGCACCCGCCCCAACCCGTCCGACTGCGAGCAACCCAGAGGGCACTCAAACTAGCAATGAGTCTAGGCCAGTTCAGCCAGCCGGGCCCATGCCCGTGGCTGCGGCCCAGGCCTTGGAAATGGCTGTTGCCACAGGGCAAGTCAATGACACCATCCCTAGTGTGGTGAGGGAAACTTTCAGCACCTACACCAATGTCACCTGGACCACACGTCAGCCTGCAGGAACCCTACTTGCCCGGATGACCCTGGGGCCGGGTCTGAACCCTTACACACTCCACCTGTCTGCCATGTGGGCTGGCTGGGGAGGATCATTTGAAATCAAAGTGATAGTATCCGGGTCTGGCTTGTATGCGGGCAAATTGTTGTGCGCACTCATACCACCTGGGGTTGATCCCAGTGCTGTGGACCAGCCTGGGGCCTTCCCCCACGCACTTGTGGATGCACGCACCACTGAGGGTGTTACCTTCACCCTTGGGGATGTCAGGGCAGTGGACTATCATGAAACGGGGGCTGGTGGGACCATTGCATGTCTGGCACTTTATGTGTACCAACCACTCATCAACCCCTTTGAAACAACCTTGTCAGCAGCCATGGTGACAATTGAGACCCGCCCTGGCCCAGACTTTGGGTTCACCCTGCTCAAGCCTCCAAACCAGACCATGGAGGTGGGACTTGACCCCAGGTCACTCCTGCCCCGCACGGCAAGAACACTGCGGGGAAACAGGTTTGGCAAACCCATTACAGCCGTGGTCATAGTGGGCATGGCACACCAAATCAATAGGCACTTCTCAGCCGAGGGCACCACGCTTGGGTGGTCCACAGCCCCAATAGGTCCTTGTGTGGGCCGCATTAATTCCAGGTACACCAACACCGGTGGCTTAGCCGTGCTCTCACTACTACCCCTGAGCAATGGGCCCCTTTACCCCAACATCGTCAACCACTACCCAGATGTGGCTGCATCAAGGGCATTCAACACCAGCACCAGTCTAACTGCCAGCACCACGTGTGGGGGAGGGCCTATGGTGGTCTTCAATGATGTGGGTGATGTGGTTGAAAACTTGACCTACCAGATGAGATTCATAGCTTCACAAGCCACTTCCCAAACACCTACACTTGTTGATTACATCAATGCAACATCAATGGCAGTGTGCAGTTTCGACAATTCCCGAGGAGACTTTGGCACAGGCCAACTCAACGTGGGTGTTGAATTGACTTACACCTGTGGCGAAACAGCAATCAATGAAAAAGTCACCACGTTCATGGATCGCCAATACACATTTGGCGCACAGGGGCCTAACAACATCATGCTGTGGGTAGAGCGTGTGCTCGGCACGCACACGGGCAACAATGCGGTGTACAGCTCGCAACCCGACACTGTGTCTGCCGCATTGCAGGGTCAGCCCTACAACATACCAGATGGGTACATGGCTGTGTGGAACGTCAATGCGGATAGTGCTGATTTCCAGATAGGCCTGAGGCGCGATGGCTTCTTCGTCACCAGTGGGGCCATTGGCACGCAAATGGTCATCTCAGAGGACACCACTTTCACCTACGCTGGCATGTTCACCCTCACCACCCCTCTTATTGGACCAAGTGGGACGACAGGAAGGTCCATTCACAGCTCACGATGAGTTGGGTTGCAGGCGCAATGCAGGGCGCTGGCCTGCTAGGAGACCTTGCAGGCACAATTGGACAAATAGTGTTGTATAACAAGCAGTTGAACATTCAGAAAAATTTTAATCAGGCTCAGTTAGAGTTGGCAAAAGAACAACTAAAACAAAATCAAAATTTGGCTAACCAGTATTATGAATTTAATGCTAATCTACCTGTTAATCAGTACAATAGTGCTGTGAGTGCTGGCTTTGATGCGGTTTCTGCACGTCAAATGGCTGGGTCGCACGAAGTGCGCTATTTTGGTGGGCAACAAACACCATTGCTCCACCAAGGGCAAATGCAACAAATGATGTTTTCATCAAAACATCTCATGCAAGCGCAGAACGTGCTTGGCACCTTTTCTCGTGGAACACCTGGTGTCACACCCAGCAAGGGGCTGCCCAAGCCACAGGGCATTACGTCCAGACCACTCAGACCACAGGGCGTTTCAGCCACCATACCTGTGGCTGGAGCCACCACCACTAACAGCAGAGTGTGATTTTCTTTCCCTGGTTACGCGTACCGAACCTTACACAGTGTGGCGGCT

>YNLF1 [organism=Sapovirus GIII.] Sapovirus GIII genes for VP1 capsid protein and VP2 minor capsid protein, complete cdsATGGAGGCACCTGCCCCAACCCGTCCGGTTGTAAGCAACCCAGAAGGCACTGAAACCAGCAATGAGTCCAGGCCAGTTCAGCCACCTGGGCCTATGCCCGTGGCTGCAGCACAGGCACTGGAAATGGCTGTCGCCACGGGACAAATCAATGACACCATCCCCAGTGTGGTTAAACAAACGTTCAGCACCTACACCAATGTCACCTGGACTACGCGCCAGCCCGCGGGGACTCTACTCGCCCGAATGTCCCTAGGGCCAGGATTAAACCCTTACACACTCCACTTGTCCGCCATGTGGGCCGGTTGGGGTGGGTCATTTGACATCAAAGTGATAATATCAGGGTCTGGTATATATGCAGGCAAACTGTTGTGCGCACTTATACCACCTGGGGTTGACCCTAGCACTGTGGACCAACCCGGGGCCTTCCCACACGCGCTTGTGGACGCGCGTACCACTGATGGTGTCACATTCACCTTGGGGGATGTCAGGGCGGTGGACTATCATGAAACAGGAGCCGGTGGAAACGTGGCATGCTTGGCACTTTATGTGTACCAGCCACTCATCAACCCCTTTGAAACCACCATATCTGCCGCCATGGTAACAGTTGAAACCCGTCCCGGTGCAGATTTTGGGTTCACCCTGCTCAAGCCTCCAAACCAAACCATGGAGGTGGGACTCGACCCCAGGTCGCTCCTGCCCCGCACTGCAAGAACACTGCGGGGGAACAGGTTTGGCAGGCCCATTAGATCTGTGATTATAGTAGGTTTGGCACAACAAATCAACAGGCATTTTTCCGCAGAGGGTACCACACTTGGTTGGTCCACGGCCCCAATTGGCCCTTGTGTAGGCCGCATCAACACCAAGTACACTGGCAATGCCGGCAAGGTGGTAGCTCAATTGCTACCTTTGAGCAATGGGCCCCTTTACCCAAACATCATCAACCACTATCCAGACGTGGCTGCATCAACAATGCTCAGTGGAGGGTCTAGCATAACTGCTGACATGACGTGTGGGGGAGGGCCCATGGTGCTCTTCAACGACGTGGGTGATGTGGTGGAGACCGTCTCCTACCAAATGAGGTTCATAGCCTCACAGGCCACATCTCAAAGCACCACACTCATCGACAAGATCAATGCAACATCAATGTCAGTGGTCAGTTTTGACAACTCCCGAAATGACTTTCCCCAATCAAATGACAATGTGGGTATTGAGTTAACCTACACTTGTGGCAACACACCGATTAACGGGAATGTCACCCAGTTCATGGACCGCCAATACACCTTTGGCGCACAAGGGCCCAACAACATCATGCTTTGGGTGGAGTCTGTGCTTGGCACGCACACTGGCAACAACAGAGTCTACAGCTCACAACCAGACACTGTGTCTGCTGCATTGCAAGGCCAGCCCTACAATATACCAGAGGGGTACATGGCCGTGTGGAACGTCAACGCGGACAGTGCTGACTTTCAAATAGGACTGAGGCGCGATGGCTTCTTTATCACCAATGGAGCCATTGGCACAGAAATGGCCATTTCAGATGATACCACCTTCACCTTCAATGGAATGTACACCCTAACCACCCCCCTCATTGGACCAAGTGGGACAACAGGAAGGTCCATTCACAGCTCACGATGAGTTGGGTTGCAGGCGCAATGCAGGGCGCTGGCCTGCTGGGTGACCTCGCAGGCACAATTGGACAAATAGTGTCGTACAATAAGCAGTTGAATATTACCAAATCTTTTAATCAGGCTCAGTTAGAGTTGGCCAGAGAACAAATGAAACAAAATCAACAGTTGGCCGACCAGTATTATGAATTTAATGCAAATTTGCCTGTCAATCAGTATAACAGTGCTGTGAGTGCTGGCTTTGACGCGGTTTCTGCACGTCAATTGGCTGGGTCGCACGAAGTGCGCTACTTTGGTGGGCAACAAACACCATTGCTCCATCAGGGGCAAACGCAACAAATGATGTTCTCATCAAAGCATCTTATGCAAGCGCAAAATGTGCTTGGCACCTTTTCTCGTGGAACACCTGGTGTCACCCCCAGCAAAGGGCTGCCCAAACCACAGGGCACCACGTCCAGACCGCTCAGACCACAGGGTGTTTCAGCCACCATACCTGTGGCTGGAGCCACCACGACCAACAGCAGAGTGTGATTTTCTTTCCCTGGTTACGCGTACCGAACCTTACACAGTGTGGCGGCT

>YNMZ1 [organism=Sapovirus GIII.] Sapovirus GIII genes for VP1 capsid protein and VP2 minor capsid protein, complete cdsATGGAGGCGCCTGCCCCAACTCGTCCGGTTGCGAGCAACCCAGAAGGCACTGAGACCAGTGATGAGTCCAGGCCAGTTCAGCCGCCTGGGCCTGTACCCGTGGCCGCAGCACAGGCTCTGGAGATGGCCGTTGCCACAGGGCAAATCAACGACACCATCCCCAGTGTGGTTAGGCAAACATTCAGTACTTACACCAATGTCACCTGGACCACGCGTCAACCCGCAGGGACCCTACTCGCCCGAATGTCCCTAGGGCCAGGATTGAACCCTTATACACTCCATTTGTCTGCCATGTGGGCCGGTTGGGGTGGGTCATTTGACATCAAGGTGGTAATATCAGGGTCTGGCATATATGCGGGCAAACTATTGTGTGCACTCATACCACCTGGGGTTGACCCTAGCACTGTGGACCAACCCGGAGCCTTTCCCCACGCACTTGTGGATGCGCGCACCACTGACGGTGTCTCATTTAACTTGGGGGATGTTAGGGCTGTGGATTACCATGAAACAGGAGCTGGTGGAAATGTGGCATGCTTGGCACTCTACGTGTACCAGCCACTCATCAACCCCTTTGAAACCACCATATCTGCTGCCATGGTGACAGTTGAGACCCGTCCCGGCGCCGACTTTGGGTTCACCCTGCTCAAGCCTCCAAACCAAACCATGGAGGTGGGACTCGACCCCAGGTCGCTCCTGCCCCGCACTGCAAGAACACTGCGGGGGAACAGGTTTGGCAGGCCCATTAGATCTGTGATTATAGTAGGTTTGGCACAACAAATCAACAGGCATTTTTCCGCAGAGGGTACCACACTTGGTTGGTCCACGGCCCCAATTGGCCCTTGTGTAGGCCGCATCAACACCAAGTACACTGGCAATGCCGGCAAGGTGGTAGCTCAATTGCTACCTTTGAGCAATGGGCCCCTTTACCCAAACATCATCAACCACTATCCAGACGTGGCTGCATCAACAATGCTCAGTGGAGGGTCTAGCATAACTGCTGACATGACGTGTGGGGGAGGGCCCATGGTGCTCTTCAACGATGTGGGTGACGTGGTGGAGGCCGTCTCCTACCAAATGAGGTTTATAGCCTCACAGGCCACATCTCAAAGCACCACACTCATCGATAAGATCAACGCAACATCAATGTCAGTGGTCAGTTTTGACAACTCCCGGAATGACTTTCCCCAATCAAATGACAACGTGGGTATTGAGTTAACCTACACTTGTGGCAACACACCGATCAATGGGAATGTCACTCAGTTCATGGACCGCCAATACACCTTTGGCGCACAGGGGCCCAATAACATCATGCTTTGGGTGGAGTCTGTGCTTGGCACGCACACTGGCAACAACAGAGTTTACAGCTCACAACCAGACACCGTGTCTGCTGCATTACAAGGCCAGCCCTACAATATACCAGAGGGGTACATGGCCGTGTGGAATGTCAACGCGGACAGTGCTGATTTCCAAATAGGACTGAGGCGCGATGGCTTCTTCATCACCAATGGAGCCATTGGCACAGAAATGGCCATTTCAGACGACACCACCTTCACCTTCAATGGAATGTACACACTAACCACCCCCCTTATTGGACCAAGTGGGACAACAGGAAGGTCCATCCACAGCTCACGATGAGTTGGGTTGCAGGCGCAATGCAGGGCGCTGGCCTGCTGGGTGACCTCGCAGGCACAATTGGACAAATAGTGTTGTACAATAAGCAGTTGAATATCACTAAATCTTTTAATCAGGCTCAGTTAGAGTTAGCCAAAGATCAAATGAAACAAAATCAACAGTTGGCTAACCAGTATTATGAATTTAATGCAAATTTGCCTGCCAATCAGTATAATAGTGCTGTCAGTGCTGGTTTTGATGCGGTTTCTGCACGTCAATTGGCTGGGTCGCACGAAGTGCGCTACTTTGGTGGACAACAAACACCAATGCTCCACCAAGGGCAAATGCAACAAATGATGTTCTCGTCAAAACACCTTATGCAAGCGCAAAACGTGCTTGGCACCTTTTCTCGTGGGACACCTGGTGTCACCCCCAGCAAAGGGCTGCCCAAACCACAGGGCATCACGTCCAGACCGCTCAGACCACAGGGTGTTTCAGCCACCATACCTGTGGCTGGAGCCACCACCACCAATAGCAGAGTGTGATTTTCTTTCCCTGGTTACGCGTACCGAACCTTACACAGTGTGGCGGCT

>YNNJ1 [organism=Sapovirus GIII.] Sapovirus GIII genes for VP1 capsid protein and VP2 minor capsid protein, complete cdsATGGAGGCACCTGCCCCAACTCGTCCGGTTGCGAGCAACCCGGAAGGCACTGAGACCAGTAACGAGTCCAAACCAGTTCAACCGCCTGGGCCTATGCCCGTGGCCGCAGCACAGGCGCTGGAAATGGCCGTTGCCACAGGGCAAATCAATGATACCATCCCCAGTGTGGTCAGACAAACATTCAGCACTTACACCAATGTTACCTGGACCACGCGTCAGCCCGCAGGGACTCTGCTCGCCCGAATGTCCCTAGGGCCAGGACTGAACCCCTACACACTCCACCTGTCCGCCATGTGGGCCGGCTGGGGTGGGTCATTTGACATCAAAGTGGTAATATCAGGGTCAGGCATATATGCGGGCAAGCTGTTGTGCGCACTTATACCACCTGGGGTCGACCCTAGCACTGTGGAACAACCTGGGGCCTTTCCACATGCGCTTGTAGATGCGCGCACCACTGACGGTGTCACATTCACCTTGGGCGATGTCAGGGCAGTAGACTATCATGAAACAGGAGCCGGTGGAAACGTGGCATGCCTGGCGCTCTATGTGTACCAGCCACTCATCAACCCCTTTGAGACCACCATATCCGCTGCCATGGTGACAGTCGAGACCCGTCCCGGCGCAGATTTTGGGTTCACCCTGCTCAAGCCCCCAAACCAAACCATGGAGGTGGGACTTGACCCTAGGTCACTCCTGCCCCGCACTGCAAGAACACTGCGGGGGAACAGGTTTGGCAGGCCCATCAGAGCTGTGCTCATAGTGGGTTTGGCACAACAAATCAATAGGCACTTTTCAGCAGAGGGTACCACACTTGGTTGGTCCACGGCCCCAATTGGCCCTTGTGTGGGCCGCGTCAACACAAAATACACTGGCAATGCGGGCAAGGTGGTAATGCAATTGCTACCTTTGAGCAACGGGCCCCTTTACCCAAACATCATCAACCACTACCCAGATGTGGCTGCGTCAACAATGCTCAGTGGAGGGTCCAGCATATCTGACAACATGACGTGTGGAGGAGGGCCTATGGTGCTTTTCAACGACGTGGGCGACGTAGTGGAGAACGTCTCCTACCAAATGAGGTTCATAGCCTCACAGGCCACATCTCAAGACACCAGACTCATTGACAAGATCAATGCAACATCAATGTCAGTGGTCAGTTTTGGCAATTCCCGGAATGACTTCCCTGAATCAAACGACAATGTGGGCATTGAGTTGACCTACACCTGTGGTTCCGCACCAATCAACGGGAACGTCACCCAGTTCATGGACCGCCAATACTCCTTTGGCGCACAGGGGCCCAACAACATCATGCTATGGGTGGAGTCTGTGCTTGGCACGCACACTGGCAATAACAGAGTCTACAGCTCACAACCAGACACTGTGTCTGCTGCATTGCAAGGCCAGCCCTACAACATACCAGAGGGGTATATGGCTGTGTGGAATGTCAATGCGGACAGTGCTGACTTTCAGATAGGGCTGAGACGTGATGGCTTCTTCATCACCAATGGAGCCATTGGCACAGAAATGGCCATCTCTGATGACACCACCTTCACTTTCAACGGCATGTACACCCTCACCACCCCCCTCATTGGACCAAGTGGGACGTCAGGAAGGTCCATTCACAGCTCACGATGAGTTGGGTTGCAGGCGCAATGCAGGGCGCTGGCCCGCTGGGTGACCTCGCAGGCACAATTGGACAAATAGTGTTGTACAACAAGCAGTTGAATATTACCAAATCTTTTAATCAGGCTCAGTTGGAGTTAGCCAAAGATCAGATGAAACAAAATCAACAGCTGGCTAACCAGTATTATGAATTTAATGCAAATTTGCCTGCCAATCAGTACAATAGTGCTGTGAGTGCTGGTTTTGACGCGGTTTCTGCACGTCAATTAGCTGGGTCACACGAAGTGCGCTACTTTGGTGGGCAGCAAACACCATTGCTTCACCAAGGACAAATGCAGCAAATGATGTTCTCGTCAAAACATCTCATGCAAGCGCAGAACGTGCTTGGCACCTTTTCTCGTGGGACACCTGGTGTCACCCCCAGCAAGGGGCTGCCCAAACCACAGGGCATCACGTCCAGACCGCTCAGGCCGCAGGGTGTTTCAGCCACCATACCAGTGGCTGGAGCCACCACCACTAGCAGCAGAGTGTGATTTTCTTTCCCTGGTTACGCGTACCGAACCTTACACAGTGTGGCGGCT

>YNSJ1 [organism=Sapovirus GIII.] Sapovirus GIII genes for VP1 capsid protein and VP2 minor capsid protein, complete cdsATGGAGGCGCCTGCCCCAACCCGTCCGGTTGCAAGCAACCCAGAAGGCACTGAGACCAGTAATGAGTCCAGACCAGTTCAGCCACCTGGGCCTGTGCCCGTGGCCGCAGCACAGGCTCTAGAGATGGCCGTCGCCACGGGGCAAATCAATGATACCATCCCCAGTGTGGTCAGACAAACATTCAGCACTTACACCAATGTCACCTGGACCACGCGTCAACCCGCAGGAACTCTACTCGCCCGAATGTCCCTAGGGCCAGGGTTAAACCCTTACACACTCCACTTGTCCGCTATGTGGGCTGGTTGGGGTGGATCATTTGACATCAAAGTGGTGATATCAGGATCTGGCATATATGCGGGCAAACTGTTGTGCGCACTTATACCACCTGGGGTTGACCCCAGCACTGTAGACCAACCTGGGGCCTTTCCCCACGCACTTGTGGATGCACGCACCACTGACGGCGTCTCATTTAATTTGGGGGATGTTAGAGCGGTGGACTACCATGAAACAGGAGCTGGTGGAAACGTGGCATGCTTGGTACTCTATGTGTACCAACCTCTCATCAACCCCTTTGAGACCACCATATCAGCTGCCATGGTGACAGTCGAGACCCGTCCCGGTGCAAACTTTGGGTTCACCCTGCTCAAGCCTCCCAACCAGACCATGGACGTGGGGTTCGACCCTAGATCACTCCTGCCCCGTACAGCAAGAACACTGCGGGGGAACAGGTTTGGCAAACCCATTAGAGCCGTGGTCATAGTGGGCACAGCACAACAAATTAATAGACACTTTTCGGCTGAGGGTACCACACTTGGCTGGTCCACGGCCCCAGTCGGCCCCTGTGTGGGTCGCGTGAACAACAAGTACACTGGTAACACTGGTAAGGTTGTGCTCCAGCTTCTGCCCCTGGACAATGGTCCCCTCTACCCCAACATCATCAACCACTACCCAGACATAGCTGCATCAACAACAATCAATGACAGCAGCGCCATATCCGACACCATGATGTGTGGTGGGGGGCCCATGGTGCTTTTTGACAACAATGGTGATGTGAGGGAGAACGTGGCTTACCAGATGAGGTTCATAGCTTCACGGGCCACATCCCAAAGTGCCACGCTCATTGACCAAATCGATGCAACATCAATGTCAGTGTGCAGTTTTAGTAATTCCCGGGCCGACCTCAGTGAGAGTGAAATCAATGTGGGCATTGAGATGACTTACACCTGTGGCAATAGGCCCATCAATGGGATTGTCACTAACTTTATGGGCCAACAATACACATTTGGGTCACAGGGAGCCAATAACGTCATGCTTTGGGTGGAAAATGTGCTCAGCTCATACACTGGAATGAATTCTGTGTACAGTTCTCAACCAGACACTGTGTCTGCTGCACTGCAGGGCCAGCCCTTCAACATACCAGAGGGACACATGGCGGTGTGGAACGTGAGCGCAGACAGTGCTGACTTCCAGATAGGTCTGAGGCGCGATGGCTACTTTGTCACCAATGGTGCCATTGGCACGAGCATAGCCATCCCAGAAGACACCACCTTCACCTACAGCGGCATCTACACCCTCACCACCCCCCTCATTGGACCAAGTGGGACATCAGGAAGGTCCATTCACGGTGCACGATGAGTTGGGTCGCAGGCGCAATGCAGGGGGCTGGCCTGCTAGGTGACCTTGCGGGCACAATTGGACAGATAGTGTTGTACAATAAGCAGTTGAATATTACCAAATCTTTTAATCAGGCTCAGATTGAAATAGCTAAAGATCAATTGAAGCAGAATGTGGAATTGGCCAACAAGTATTATGAGTTTAATGAGAATTTGCCTGTCAATCAGTACAATAGTGCTGTGAGTGCTGGCTTTGACCCGGTTTCTGCACGTCAATTGGCTGGGTCGCGTGAGGTGCGTTACCTTGGTGGGCAACAAACACCACTGCTCCACCAAGGGCAAATGCAACAAATGATGTTCTCATCAAAGCACCTCATGCAGGCACAAAACGTGCTTGGCACGTTTTCCCGTGGAACACCAGGTGTCACCCCCAGCAAGGGGTTGCCCAAGCCACAGGGCGTCACTTCCAGACCACTGGGCACTGCAGCCACTGTACCCCTGGCTGGTGCCACCACCACTCATAGCAAAGTGTGATTTTCTTTCCCTGGTTACGCGTACCGAGCCTTACACAGTGTGGCGGCT

>YNTH1 [organism=Sapovirus GIII.] Sapovirus GIII genes for VP1 capsid protein and VP2 minor capsid protein, complete cdsATGGAGGCGCCCGCCCCAATCCGTCCGGTTGCGAGCAACCCAGAAGGCACTGAGACCAGTGATGAGTCCAGACCAGTTCAGCCGCCCGGGCCTGTGCCCGTGGCCGCAGCACAGGCTCTGGAGATGGCCGTTGCTACGGGACAAATTAATGACACCATCCCTAGCGTGGTTAGACAAACATTCAGTACTTACACCAATGTCACCTGGACCACGCGTCAACCCGCAGGAACCCTACTCGCCCGAATGTCCCTAGGGCCAGGATTAAACCCTTACACGCTCCACTTGTCCGCCATGTGGGCTGGCTGGGGTGGATCATTTGACATCAAAGTGGTGATATCAGGGTCTGGCATATATGCGGGCAAACTGTTGTGCGCACTTATACCACCAGGGGTTGACCCCAGCACTGTGGACCAACCTGGGGCCTTTCCCCACGCACTTGTGGATGCGCGCACCACTGACGGTGTCTCATTCAACTTGGGGGATGTTAGGGCGGTGGACTACCATGAAACAGGAGCCGGTGGAAACGTGGCATGCTTGGCACTTTATGTGTACCAGCCTCTCATCAACCCCTTTGAGACCACCATATCTGCTGCCATGGTGACAGTCGAGACCCGCCCCGGTGCAGACTTCGGGTTCACCCTGCTCAAGCCCCCAAACCAAACCATGGAGGTGGGACTCGACCCCAGGTCACTCTTGCCCCGCACTGCAAGAACATTGCGAGGGAACAGGTTTGGTAGGCCCATCAGGTCTGTGCTTATAGTGGGCTTGGCGCAACAAATCAACAGGCATTTTTCAGCAGAGGGCACCACACTTGGATGGTCCACGGCCCCAATTGGCCCCTGTGTGGGCCGCATTAACACAAAGTACACTGGCAATGCAGGCAAGGTGGTGGCTCAACTGCTGCCTTTGAGCAATGGACCCCTTTACCCAAACATCATCAACCACTACCCAGACGTGGCTGCATCAACAATGCTTAGTGGAGGGTCTAGCATAACTGCTGATATGACGTGTGGGGGAGGACCCATGGTGCTGTTCAACGATGTGGGCGATGTGGTGGAGAACGTCTCCTACCAAATGAGGTTCATAGCCTCACAGGCCACTTCTCAAAGCACCACACTCATCGACAAGATTAATGCAACATCAATGTCTGTGGTCAGTTTTGACAACTCCCGAAACGACTTCCCCCAATCAAACGACAATGTGGGTATTGAGTTAACCTACACTTGTGGCAACACACCAATCAACGGGAATGTCACCCAGTTCATGGACCGCCAATACACCTTTGGCGCACAGGGGCCCAATAACATCATGCTTTGGGTGGAGTCTGTGCTTGGCGCGCACACTGGCAACAACAGAGTTTACAGCTCACAACCAGACACCGTGTCTGCCGCACTGCAGGGCCAGCCCTACAACATACCAGAGGGGTACATGGCCGTGTGGAACGTCAACGCGGACAGTGCCGACTTTCAAATAGGGTTGAGGCGCGATGGCTACTTTATCACCAATGGAGCCATTGGCACAGAAATGGCCATTTCAGATGACACCACCTTCACCTTCAACGGCATGTACACTCTCACCACCCCCCTCATTGGACCAAGTGGGACATCAGGAAGGTCCATTCACAGTGCACGATGAGTTGGGTCGCAGGCGCAATGCAGGGCGCTGGCCTGGCAGGTGATCTTGTCGGCACAGTTGGACAAATTGTGTTATACAATAAGCAGTTGAATATTCAGAAATCTTTTAATCAGGCCCAGATGGAGTTGGCTAAAGAACAATTGAAGCAAAATCAACATATGGCCAACCAGTTTTATGAATTTAATTCAAATTTACCCGTCAATCAGTACAATAGTGCTGTGAGTGCTGGCTTTGACTCAGTCTCTGCACGCCAATTGGCTGGGTCCCGCGAGGCGCGCTACTTTGGTGGACAGCAAACACCTTTGCTCCACCAAGGGCAGATGCACCAAATGATGTTTTCATCAAAACACTTGATGCAAGCACAAAATGTGCTTGGTACTTTTTCCCGTGGGACGCCTGGTGTGACCCCCAGCAAAGGGTTGCCCAGACCGCAGGGCATCACCCCCAGACCACAGAGCAACGCGGCCACCCTGCCCGTGGCTGGAGCCACCACTACTCACAGCAAAGTGTGATTTTCTTTCCCTGGTTACGCGTACCGAACCTTACACAGTGTGGCGGCT

>YNXW1 [organism=Sapovirus GIII.] Sapovirus GIII genes for VP1 capsid protein and VP2 minor capsid protein, complete cdsATGGAGGCACCTGCCCCAACCCGTCCGGTTGCAAGCAACCCAGAAGGCACTGAGACCAGTAATGAGTCCAGACCAGTTCAGCCGCCTGGGCCTATGCCCGTGGCCGCAGCACAGGCTCTGGAAATGGCTGTTGCCACGGGGCAAATCAATGACACCATCCCCAGTGTGGTCAGACAAACATTTAGCACTTACACCAATGTCACCTGGACCACGCGCCAGCCCGCAGGAACTCTACTTGCCCGAATGTCCCTAGGGCCAGGACTGAACCCTTACACACTTCATTTGTCCGCCATGTGGGCCGGCTGGGGTGGATCATTTGACATCAAGGTGGTAATATCAGGGTCTGGCATATATGCGGGTAAGCTGTTGTGTGCACTCATACCACCTGGGGTCGACCCTAGCACTGTGGACCAACCCGGAGCCTTTCCCCACGCGCTTGTGGATGCGCGCACCACTGACGGTGTTTCATTCAATTTGGGGGATGTTAGGGCGGTGGACTACCATGAAACAGGAGCTGGTGGAAACGTGGCATGTTTGGCACTCTACGTGTACCAACCACTCATTAACCCCTTTGAAACCATCATATCCGCTGCCATGGTGACGGTCGAGACCCGCCCCGGCGCGGACTTTGGGTTCACCCTGCTCAAGCCCCCAAACCAAACCATGGAGGTGGGATTTGATCCCAGGTCGCTCCTGCCCCGCACGGCAAGAACGCTTCGGGGGAACAGGTTTGGCAGACCTATCACAGCTGTGGTTATAGTAGGCCTGGCACAACAAATCAACAGACATTTTTCAGCTGATGGCACCACACTTGGCTGGTCCACGGCCCCAGTTGCCCCATGTGTGGCACGTGTCAATGGAAAGTACACCGGCACCAACGGCATGGCAGTGTTTCAACTCCAACCCTTGAGCAATGGGCCCCTTTACCCCAACATCATCAACCACTACCCAGACGTGGCTGCATCAACAATGTTCAGTGGAGGGTCTAGCATAACTTCTGACATGACGTGTGGGGGAGGACCCATGGTGCTCTTCAACGACGTGGGTGATGTGGTGGAGACCGTCTCCTACCAAATGAGGTTCATAGCCTCACAGGCCACATCTCAAAGCACCACACTCATCGACAAGATCAATGCAACATCAATGTCAGTGGTCAGTTTTGACAACTCCCGAAATGACTTTCCCCAATCAAATGACAATGTGGGTATTGAGTTAACCTACACTTGTGGCAACACACCGATTAACGGGAATGTCACCCAGTTCATGGACCGCCAATACACCTTTGGCGCACAAGGGCCCAACAACATCATGCTTTGGGTGGAGTCTGTGCTTGGCACGCACACTGGCAACAACAGAGTCTACAGCTCACAACCAGACACTGTGTCTGCTGCATTGCAAGGCCAGCCCTACAATATACCAGAGGGGTACATGGCCGTGTGGAACGTCAACGCGGACAGTGCTGACTTTCAAATAGGACTGAGGCGCGATGGCTTCCTTATCACCAATGGAGCCATTGGCACAGAAATGGCCATTTCAGATGATACCACCTTCACCTTCAATGGAATGTACACCCTAACCACCCCCCTCATTGGACCAAGTGGGACAACAGGAAGGTCCATTCACAGCTCACGATGAGTTGGGTTGCTGGCGCAATGCAAGGTGCTGGCCTGCTGGGTGATCTCGCGGGCACGATTGGACAAATTGTGTTGTATAATAAGCAGTTGAATATTCAGAAATCTTTTAATCAGGCCCAGATGGAGTTGGCTAAAGAACAATTGAAACAAAATGAGAAATTGGCCAACCAATATTACAAATTTAATGAAAGTTTGCCTGTTAATCAGTACAATAGTGCAGTGAGTGCTGGTTTCGACCCAGTTTCCGCGCGTCAATCGGCCGGTTCACGTGAAGTGCGTTACCTTGGTGGGCAACAAACACCACTGCTTCACCAAGGACGGATGCAACAAATGATGTTCTCATCAAAACACCTGATGCAAGCGCAACACGTGGTCGGCACTTTTTCCCGCGGGATACCTGGTGTCACTCCTAGTAAGGGATTGCCAAGACCACAGGGCATCACACCCAGGCCACAGGGTGTTACAGCTAGTGTGCCCGTGGCTGGCGCCACCACCACCCACAGTAGAGTATGATTTTCTTTCCCTGGTTACGCGTACCGGACCTTACACAGTGTGGCGGCT

>YNYM1 [organism=Sapovirus GIII.] Sapovirus GIII genes for VP1 capsid protein and VP2 minor capsid protein, complete cdsATGGAGGCGCCTGCCCCAACCCGTCCAGTTGCGAGCAACCCAGAAGGCACTGAGACCAGTGATGAGTCCAGGCCAATTCAGCCGTCTGGGCCGGTGCCCGTGGCCGCGGCACAGGCTTTGGAGATGGCCGTTGCTACAGGGCAAATCAATGACACCATCCCCAGTGTGGTCAGGCAAACATTCAGTACCTATACCAATGTCACCTGGACCACGCGTCAACCCGCAGGGACCCTGCTCGCCCGAATGTCCCTAGGGCCGGGATTAAACCCTTACACACTCCACTTGTCCGCCATGTGGGCCGGTTGGGGTGGGTCATTTGACATCAAGGTGGTAATTTCAGGGTCTGGCATATATGCGGGCAAACTGTTGTGTGCACTTATACCACCTGGGGTTGACCCCAGCACTGTGGACCAACCCGGAGCCTTCCCCCACGCACTTGTGGACGCGCGCACCACTGATGGCGTTTCATTCAACTTGGGGGATGTTAGGGCGGTGGACTACCATGAAACAGGAGCTGGTGGAAACGTGGCATGCTTGGCACTTTACGTGTACCAGCCACTCATCAACCCCTTTGAAACCACCATATCTGCTGCCATGGTGACAGTCGAAACCCGTCCCGGCGCAGACTTTGGGTTCACCCTGCTCAAGCCTCCAAATCAGACCATGGAGGTGGGGCTCGACCCCAGGTCACTCCTGCCCCGAACGGCAAGAACGTTGCGGGGCAACAGGTTTGGCAGACCTATTAAAGCAGTGGTTATAGTTGCCATGGCACACCAGATCAACAGGCACTTTTCTGCTGAGGGCACCACACTTGGCTGGTCCACGGCCCCTATTGGCCCTTGTGTGGCCCAGGTCGTTGAAAGGCGCACTAACACCACAGGCCTAGCAGTGTTTCAGCTGGGGCCCTTGAGTAACGGGCCCCTTTACCCTAACATCATCAACCACTACCCAGATGTGGCTGCATCAACAACGCTCAGTGGAGGGTCTGCCATATCCAATGACACCACATGTGGGGGAGGGCCCATGGTGGTCTTTGACAATCAGGGTGATGTGAATGAAAATGTGGCCTACCAGATGAGATTCATAGCCTCACGCGCCACCTCTCAAAATCCCACGCTTGTTGAACACATCAATGCAGCATCAATGGCGGTGTGTAGCTTTGGCAACAGCCGAGGGGACCTCACTCAATCCCAACTAAATGTGGGCATTGAGCTCACCAACACCTGTGGCAACACACCCATTAATGGGATAGTCACCACCTTCATGGACCGCCAATATGCATTTGGCCCACAGGGGCCCAATAATGTCATGCTTTGGGTGGAATCTGTGCTTGGCACACACACAGGCAACAACACTGTGTATAGTTCACAGCCTGACACTGTTTCTGCTGCACTACAGGGCCAGCCCTTCAACATACCAGATGGGCACATGGCAGTGTGGAATGTCAACGCGGACAGTGCTGACTTCCAAATAGGGCTGAGACGCGATGGCTTCTTCGTCACTAACGGAGCCATTGGCACGTCCATAACCATCTCAGAAGATACCACCTTCACCTACAATGGCATGTATTCCCTCACTACCCCTCTCATTGGACCAAGTGGGACAACAGGAAGGTCCATTCACAGCACACGATGAGTTGGGTTGCGGGTGCAATGCAGGGCGCTGGCCTGCTGGGTGACCTCGCAGGCACGATTGGACAAATAGTGTTGTATAATAAGCAGTTGAACATTACCAAATCTTTTAATCAGGCTCAGTTAGAGTTGGCCAAAGATCAGATGAAACAAAATCAACAGTTGGCCAACCAGTATTATGAATTTAATGCAAATTTGCCTGCCAATCAGTACAATAGTGCTGTGAGTGCTGGTTTTGATGCGGTTTCTGCACGCCAACTGGCTGGGTCGCACGAAGTGCGCTACTATGGTGCGCAACAAACACCATTGCTCCACCAAGGGCAAATGCAACAGATGATGTTCTCATCAAAACATCTTATGCAAGCGCAAAACATGCTTGGCACCTTCTCTCGTGGGACACCTGGTGTCACCCCCAGCAAAGGGCTGCCCAAACCACAGGGCATCACGTCCAAACCGCTCAGACCACAGGGTGTCTCAGCTACTATACCCGTGGCTGGAGCCACCACCACTAACAGCAGAGTGTGATTTTCTTTCCCTGGTTACGCGTACCGAACCTTACACAGTGTGGCGGCT

>YNYM2 [organism=Sapovirus GIII.] Sapovirus GIII genes for VP1 capsid protein and VP2 minor capsid protein, complete cdsATGGAGGCGCCCGCCCCAACCCGTTCGGTGGTGAGCAACCCAGAAGGCACTCAAACCAGCAATGAGTCTAGACCGGTCCAGCCAGCCGGGCCTATGCCCGTGGCCACAGCCCTGGCGCTTGAGATGGCTGTTGCCACTGGTCAAGTCAATGACACCATCCCCAGTGTGGTCAGGGACACCTTTAGTACCTACACCAATGTCACCTGGACCACACGTCGGCCTGCAGGAACCCTGCTTGCCCGAATGACCCTGGGGCCAGGTCTGAACCCCTACACGCTCCACCTGTCTGCCATGTGGGCTGGCTGGGGAGGATCATTTGAAATCAAAGTGATAGTATCCGGGTCTGGCTTGTATGCGGGCAAATTGTTGTGCGCACTCATACCACCTGGGGTTGACCCCAGTGCTGTGGATCAGCCTGGGGCCTTCCCCCACGCACTTGTGGATGCACGCACCACTGAAGGCGTTACCTTCACCCTTGGGGATGTCAGGGCAGTGGACTATCATGAAACAGGGGCTGGTGGGACCATTGCATGTCTGGCACTTTACGTGTACCAACCACTCATTAACCCCTTTGAAACAGCCTTGTCGGCAGCCATGGTGACAATTGAAACCCGCCCTGGCCCAGACTTTGGGTTCACCCTGCTCAAGCCTCCAAACCAGACCATGGAGGTGGGACTTGACCCCAGGTCACTCCTGCCCCGCACGGCAAGAACACTGCGGGGAAATAGGTTTGGCAGACCCATTACAGCCGTGGTCATAGTAGGCATGGCACACCAAATTAACAGGCACTTCTCAGCCGAGGGCACCACGCTTGGGTGGTCCACAGCCCCAATAGGCCCTTGTGTGGGCCGTATTAATTCCAGGTACACCAATACCGGTGGCTTAGCCGTGCTCTCACTACTACCCCTGAGCAATGGGCCCCTTTACCCCAACATCGTCAACCACTACCCAGATGTGGCTGCATCAAAGGCATTCAACACTAGCACCAGTCTGGCTGCCAACACCACGTGTGGGGGAGGGCCTATGGTGGTCTTCAATGATGTGGGTGATGTGGTAGAAAACTTGACCTACCAGATGAGATTCATAGCTTCACAAGCCACTTCCCAAACACCTACACTTGTTGATTACATCAATGCAACATCGATGGCAGTGTGCAGTTTTGACAATTCCCGAGGAGACTTTGGCACAGGCCAACTCAACGTGGGTGTTGAATTAACTTACACCTGTGGCGAGACAGCAATCAATGAAAAGGTCACCACGTTCATGGATCGCCAATACACTTTTGGCGCACATGGGCCCAACAACATCATGCTGTGGGTAGAGCGTGTACTCGGCACGCACACGGGCAACAATGCGGTGTACAGCTCGCAACCCGACACTGTCTCTGCCGCATTGCAGGGTCAGCCCTACAACATACCAGATGGGTACATGGCTGTGTGGAACGTCAATGCGGACAGTGCTGATTTCCAGATAGGCCTGAGGCGCGATGGCTTCTTCGTCACCAGTGGGGCCATTGGCACGCAAATGATCATCTCAGAGGATACTACTTTCACCTACGCTGGCATGTTCACTCTCACCACCCCCCTTATTGGACCAAGTGGGACAACAGGAAGGTCCATTCACAGCTCACGATGAGTTGGGTTGCAGGCGCAATGCAGGGCGCTGGCCTGCTAGGAGACCTTGCAGGCACAATTGGACAAATAGTGTTGTATAACAAGCAGCTGAATATTCAGAAAAATTTCAATCAGGCTCAGTTAGAGTTGGCCAAAGAACAACTGAAACAAAATCAAAATTTGGCTAACCAGTATTATGAATTTAATGCTAATTTACCTGTTAATCAGTACAATAGTGCTGTGAGTGCTGGTTTTGACGCGGTTTCTGCACGTCAAATGGCTGGGTCGCACGAAGTGCGCTATTTCGGTGGGCAACAAACACCATTGCTTCACCAAGGGCAAATGCAACAAATGATGTTTTCATCAAAACATCTCATGCAAGCGCAAAACGTGCTTGGCACCTTTTCTCGTGGAACACCTGGCGTCACACCCAGTAAGGGACTGCCCAAGCCACAGGGCATCACGTCCAGACCACTCAGACCACAGGGCGTTTCAGCCACCATACCTGTAGCTGGAGCCACCACCACTAACAGCAGAGTGTGATTTTCTTTCCCTGGTTACGCGTACCGAACCTTACACAGTGTGGCGGCT

>YNYM3 [organism=Sapovirus GIII.] Sapovirus GIII genes for VP1 capsid protein and VP2 minor capsid protein, complete cdsATGGAGGCGCCTGCCCCAACCCGTCCAGTTGCGAGCAACCCAGAAGGCACTGAGACCAGTGATGAGTCCAGGCCAGTTCAGCCGTCTGGGCCGGTGCCCGTGGCCGCGGCACAGGCTTTGGAGATGGCCGTCGCTACAGGGCAAATCAATGACACCATCCCCAGTGTGGTCAGGCAAACATTCAGTACCTATACCAATGTCACCTGGACCACGCGTCAACCCGCAGGGACCCTGCTCGCCCGAATGTCCCTAGGGCCGGGATTAAACCCTTACACACTCCACTTGTCCGCCATGTGGGCCGGTTGGGGTGGGTCATTTGACATCAAGGTGGTAATTTCAGGGTCTGGCATATATGCGGGCAAACTGTTGTGTGCACTTATACCACCTGGGGTTGACCCCAGCACTGTGGACCAACCCGGAGCCTTCCCCCACGCACTTGTGGACGCGCGCACCACTGATGGCGTTTCATTCAACTTGGGGGATGTTAGGGCGGTGGACTACCATGAAACAGGAGCTGGTGGAAACGTGGCATGCTTGGCACTTTACGTGTACCAGCCACTCATCAACCCCTTTGAAACCACCATATCTGCTGCCATGGTGACAGTCGAAACCCGTCCCGGCGCAGACTTTGGGTTCACCCTGCTCAAGCCTCCAAACCAAACCATGGAGGTGGGACTTGACCCCAGGTCGCTCCTGCCCCGCACTGCAAGAACACTGCGGGGAAACAGGTTTGGCAGGCCCATCAGATCTGTGATTATAGTAGGTTTGGCACAACAAATTAACAGGCACTTTTCCGCAGAGGGTACCACACTTGGTTGGTCCACGGCCCCAATTGGCCCCTGTGTAGGCCGCATCAACACCAAGTACACTGGTAATGCGGGCAAGGTGGTGGCACAACTGCTACCTTTGAGCAATGGGCCCCTTTACCCAAATATCATCAACCACTACCCAGACGTGGCTGCATCAACAATACTCAGCGGAGGGTCTAGCATAACTGCTGACATGACGTGTGGGGGAGGGCCCATGGTGGTCTTTGACAATCAGGGTGATGTCAATGAAAATGTGGCCTACCAAATGAGGTTCATAGCTTCACGCGCCACCTCTCAAAACCCCACGCTTGTTGAACACATCAATGCAACATCAATGGCGGTGTGCAGCTTTGGCAACAGCCGAGGGGACCTCACTCAATCCCAACTCAATGTGGGCATTGAACTCACCAACACCTGTGGCAACACACCGATCAATGGGAATGTCACCCAGTTCATGGACCGCCAATACACTTTTGGTGCACAAGGGCCCAACAACATCATGCTTTGGGTGGAGTCTGTGCTTGGCACGCACACTGGCAACAACAGAGTCTACAGCTCACAACCAGACACCGTGTCTGCTGCATTGCAAGGCCAGCCCTACAACATACCAGACGGGTACATGGCCGTGTGGAACGTCAATGCGGACAGTGCTGACTTTCAAATAGGGCTGAGGCGCGATGGCTTCTTCATCACCAATGGAGCCATTGGCACTGAAATGGCCATTTCAGATGACACCACCTTCACCTTCAATGGAATGTACACCCTAACCACCCCCCTCATTGGACCAAGTGGGACAACAGGAAGGTCCATCCACAGCTCACGATGAGTTGGGTTGCAGGTGCAATGCAAGGTGCTGGCCTGCTGGGTGACCTGGCGGGCACAATTGGACAAATAGTGTTGCATAATAAGCAGTTGAATATTCAGAAATCTTTCAATCAGGCCCAAATAGAGTTGGCTAAGGAACAGCTGAAACAAACTAAGGATCTAGCCAACCAGTATTATAAATTTAATGAAAATTTGCCTGTTAATCAATACAATAGTGCGGTGAGGGCTGGTTTTGACGCAGTTTCTGCGCGCCAATTGGCCGGCTCACGTGAAGTGCGTTACCTTGGTGGGCAGCAAACACCACTGCTCCATCAAGGACAGATGCAACAACTGATGTTTTCACCAAGACATCTGATGCAAGCGCAGCATGTGGTTGGCACTTTCTCTCGTGGGACCCCTGGCGTCACCCCCAGCAAGGGGTTGCCAAGACCACAAGGCATCACGCCCAGACCACAGGGTGTCACGGCCAACGTACCCGTGGCTGGCGTCACCTCCTCTCACAGCAAAGTGTGATTTTCTTTCCCTGGTTACGCGTACCGGACCTTACACAGTGTGGCGGCT

>YNYM4 [organism=Sapovirus GIII.] Sapovirus GIII genes for VP1 capsid protein and VP2 minor capsid protein, complete cdsATGGAGGCACCTGCCCCAACCCGTTCGGTTGCAAGCAACCCAGAAGGCACTGAAACCAGTGATGAGTCCAGACCAGTTCAGCCGCCTGGGCCTATGCCCGTGGCCGCAGCACAGGCTCTGGAAATGGCCGTTGCCACGGGGCAAATCAATGACACCATCCCCAGTGTGGTCAGACAAACATTTAGCACTTACACCAATGTCACCTGGACCACGCGTCAGCCCGCGGGAACTCTACTCGCCCGAATGTCCCTAGGGCCAGGATTAAACCCCTACACACTCCACTTGTCCGCCATGTGGGCCGGCTGGGGTGGGTCGTTTGACATTAAAGTAGTGATATCAGGGTCTGGCATATACGCGGGCAAACTTTTGTGCGCACTCATACCACCTGGGGTTGACCCTAGCACTGTAGACCAACCTGGTGCCTTTCCACACGCACTTGTGGATGCGCGCACCACTGACGGTGTCACATTCACCTTGGGGGATGTCAGGGCAGTGGACTATCATGAAACAGGGGCTGGTGGAAACGTGGCATGTTTGGCGCTCTACGTGTACCAGCCACTCATCAACCCTTTTGAGACCACCATATCCGCCGCCATGGTGACTGTTGAGACCCGTCCTGGTGCGGATTTTGGGTTCACCCTGCTCAAGCCCCCAAATCAGACCATGGAGGTGGGGCTCGACCCCAGGTCACTCCTGCCCCGAACGGCAAGAACGTTGCGGGGCAACAGGTTTGGCAGACCTATTAAAGCAGTGGTTATAGTTGCCATGGCACACCAGATCAACAGGCACTTTTCTGCTGAGGGCACCACACTCGGCTGGTCCACGGCCCCTATTGGCCCTTGTGTGGCCCAGGTCGTTGAAAGGCGCACTAACACCACAGGCCTAGCAGTGTTTCAGCTGGGGCCCTTGAGTAACGGGCCCCTTTACCCTAACATCATCAACCACTACCCAGATGTGGCTGCATCAACAACGCTCAGTGGAGGGTCTGCCATATCCAATGACACCACATGTGGGGGAGGGCCCATGGTGGTCTTTGACAATCAGGGTGATGTGAATGAAAATGTGGCCTACCAGATGAGGTTCATAGCCTCACGCGCCACCTCTCAAAATCCCACGCTTGTTGAACACATCAATGCAACATCAATGGCGGTGTGTAGCTTTGGCAACAGCCGAGGGGACCTCACTCAATCCCAACTAAATGTGGGCATTGAGCTCACCAACACCTGTGGCAACACACCCATTAATGGGATAGTCACCACCTTCATGGACCGCCAATATGCATTTGGCCCACAGGGGCCCAATAATGTCATGCTTTGGGTGGAATCTGTGCTTGGCACACACACAGGCAACAACACTGTGTATAGTTCACAGCCTGACACTGTTTCTGCTGCACTACAGGGCCAGCCCTTCAACATACCAGATGGGCACATGGCAGTGTGGAATGTCAACGCGGACAGTGCTGACTTCCAAATAGGGCTGAGACGCGATGGCTTCTTCGTCACTAACGGAGCCATTGGCACGTCCATAACCATCTCAGAAGACACCACCTTCACCTACAATGGCATGTATTCCCTCACTACCCCTCTCATTGGACCAAGTGGGACAACAGGAAGGTCCATTCACAGCACACGATGAGTTGGGTTGCAGGTGCAATGCAAGGTGCTGGCCTGCTGGGTGACCTGGCGGGCACAATTGGACAAATAGTGTTGCACAACAAGCAGTTGAATATTCAGAAATCTTTCAATCAGGCCCAAATAGAGTTGGCTAAGGAACAGCTGAAACAAAATAAGGAGCTAGCCAACCAGTATTACAAATTTAATGAAAATTTGCCTGTTAATCAATATAATAGTGCAGTGAGGGCTGGTTTTGATGCAGTTTCTGCGCGCCAATTGGCCGGCTCACGTGAAGTGCGTTACCTTGGTGGGCAGCAAACACCACTGCTCCATCAAGGGCAGATGCAACAATTGATGTTTTCACCAAGACATCTGATGCAAGCGCAGCATGTGGTTGGCACTTTCTCTCGTGGAACCCCTGGCGTCACCCCCAGTAAGGGGTTGCCAAGACCACAAGGCATCACGCCCAGACCACAGGGTGTTACGGCCAACGTACCCGTGGCTGGCGTCACCTCCTCTCACAGCAAAGTGTGATTTTCTTTCCCTGGTTACGCGTACCGGACCTTACACAGTGTGGCGGCT

>YNXW2 [organism=Sapovirus GIII.] Sapovirus GIII genes for VP1 capsid protein and VP2 minor capsid protein, complete cdsATGGAGGCACCTGCCCCAACTCGTCCGACTGCGAGCAACCCAGAGGGTACCCAAACCAGTAATGAGTCCAGACCAGTCCAACCAGCCGGACCCATGCCCGTGGCGGCGGCCCAAGCGCTGGAAATGGCTGTCGCCACTGGGCAAATCAATGACACAATCCCCAGTGTGGTCAGGGAAACTTTCAGCACCTACACCAATGTCACCTGGACCACACGCCAGCCTGCAGGGACTATGCTTGCCCGTATGTCCCTAGGGCCCGGCTTGAACCCCTACACACTTCACTTGTCTGCTATGTGGGCCGGCTGGGGCGGGTCGTTTGAGGTAAAGGTCATCATATCAGGGTCTGGCATGTATGCAGGTAAGTTGCTGTGCGCGCTCATACCACCTGGGGTCAACCCTGCCACTGTGGACCAACCTGGGGCTTTCCCACATGCACTTGTGGACGCGCGCATCACTGATGGTGTCACTTTCACCTTGGGGGACGTCAGAGCGGTGGATTACCACGAAACTGGCACTGGTGGCAATGTAGCACAACTGGCACTTTATGTGTACCAACCACTCATCAACCCCTTTGAAACCAACGTGTCGGCAGCCATGGTGACTGTTGAGACCCGCCCTGGTCCAGACTTTGGGTTCACCCTGCTCAAGCCTCCAAACCAGACCATGGAGGTGGGACTTGACCCCAGGTCGCTTTTGCCCCGAACGGCAAGGACCTTGCGAGGCAACAGGTTTGGTAGACCTATTAAAGCCGTGGTCATAGTGGGCATAGCGCACCAGATCAACAGGCACTTCTCTGCTGAGGGCACCACACTTGGTTGGTCTACGTCTCCTATTGGCCCTTGTGTAGGCCAGGTTGTTGCAAAGCACTCCAGCACCACGGGTATGGCAGTGTTCCAGCTAGGGCCGCTAAGCAATGGGCCCCTCTATCCCAACATCATCAATCATTACCCAGACGTGGCTGCATCCACAACACTCCGCGGAGGTTCCACTGTGTCTGGTGACACCACATGTGGGGGGGGGCCCATGGTGATTTTTGACAACAACGGCGACGTAACTGAAAACGTGGCCTACCAGATGAGATTCATAGCCTCACGTGCCACCTCTCAAAACCCCACGCTCGTTGCACACATCGACGCAACGTCGATGGCGGTGTGCAGCTTTGGCAACGGGCGGGGAGACCTTACCCAAGCCCAATTCAATGTTGGCATTGACCTCACCTATACCTGTGGCAATACACCAATCAATGGGTTGGTCACCACCTTCATGGACCGCCAGTATGCATTTGGCGCAACGGGGCCCAACAACATCATGCTCTGGGTGGAGTCTGTGCTCGGCACACACACAGGCAACAACTCTGTGTACAGCTCACAGCCAGACACTGTGTCCGCTGCTCTACAGGGTCAGCCCTTCAACATACCCGACGGGCACATGGCAGTGTGGAACGTTAACGCGGACAGTGCTGACTTCCAGATAGGGCTGAGACGCGATGGCTTCTTCGTCACCAACGGAGCCATTGGCACGTCCATAACCATTTCAGAAGACACCACCTTCACTTACAATGGCATGTATTCCCTCACCACCCCCCTCATTGGACCAAGTGGGACATCAGGAAGGTCCATTCACGGCACACGATGAGTTGGATCGCAGGTGCAATGCAAGGTGCTGGTCTGCTGGGTGATCTGGCGGGCACAATTGGACAAATAGTGTTGCACAACAAGCAGTTGAATATTCAGAAATCTTTCAATCAGGCCCAGATAGAGTTGGCTAAAGAACAGCTGAAACAAAATGAGAAGCTAGCCAACCAGTATTACAAATTTAATGAAAATTTACCTGTTAATCAGTACAATAGTGCAGTGGGGGCTGGTTTTGACGCAGTCTCTGCGCGTCAGTTGGCCGGCTCACGTGAAGTGCGTTACCTTGGTGGGCAGCAAACACCACTGCTTCATCAAGGACAGATGCAACAACTGATGTTTTCACCAAGACATCTGATGCAAGCGCAGCATGTGGTTGGCACTTTTTCCCGTGGGACCCCTGGCGTCACCCCCAGTAAGGGGTTGCCAAGACCACAAGGCATCACACCCAGACCACAGGGTGTTACAGCCAACGTGCCCGTGGCTGGCGTCACCTCTTCTCACAGCAAAGTGTGATTTTCTTTCCCTGGTTACGCGTACCGGACCTTACACAGTGTGGCGGCT

>YNTH2 [organism=Sapovirus GIII.] Sapovirus GIII genes for VP1 capsid protein and VP2 minor capsid protein, complete cdsATGGAGGCGCCCGCCCCAATCCGTCCGGTTGCGAGCAACCCAGAAGGCACTGAGACCAGTGATGAGTCCGGACCAGTTCAGCCGCCCGGGCCTGTGCCCGTGGCCGCAGCACAGGCTCTGGAGATGGCCGTTGCTACGGGACAAATTAATGATACCATCCCTAGCGTGGTCAGACAAACATTCAGTACCTACACCAATGTCACCTGGACCACGCGTCAACCCGCAGGAACCCTACTCGCCCGAATGTCCCTAGGGCCAGGATTAAACCCTTACACGCTCCACTTGTCCGCCATGTGGGCTGGCTGGGGTGGATCATTTGACATCAAAGTGGTGATATCAGGGTCTGGCATATATGCGGGTAAACTGTTGTGCGCACTTATACCACCAGGGGTTGACCCCAGCACTGTGGACCAACCTGGGGCCTTTCCCCACGCACTTGTGGATGCGCGCACCACTGACGGTGTCTCATTCAACTTGGGGGATGTTAGGGCGGTGGACTACCATGAAACAGGAGCCGGTGGAAACGTGGCATGCTTGGCACTTTATGTGTACCAGCCTCTCATCAACCCCTTTGAGACCACCATATCTGCTGCCATGGTGACAGTCGAGACCCGCCCCGGTGCAGACTTCGGGTTCACCCTGCTCAAGCCCCCAAACCAAACCATGGAGGTGGGACTCGACCCCAGGTCACTCTTGCCCCGCACTGCAAGAACATTGCGAGGGAACAGGTTTGGTAGGCCCATCAGGTCTGTGCTTATAGTGGGCTTGGCGCAACAAATCAACAGGCATTTTTCAGCAGAGGGCACCACACTTGGATGGTCCACGGCCCCAATTGGCCCCTGTGTGGGCCGCATTAACACAAAGTACACTGGCAATGCAGGCAAGGTGGTGGCTCAACTGCTGCCTTTGAGCAATGGACCCCTTTACCCAAACATCATCAACCACTACCCAGACGTGGCTGCATCAACAATGCTTAGTGGAGGGTCTAGCATAACTGCTGATATGACGTGTGGGGGAGGACCCATGGTGCTGTTCAACGATGTGGGCGATGTGGTGGAGAACGTCTCCTACCAAATGAGGTTCATAGCCTCACAGGCCACTTCTCAAAGCACCACACTCATCGACAAGATTAATGCAACATCAATGTCTGTGGTCAGTTTTGACAACTCCCGAAACGACTTCCCCCAATCAAACGACAATGTGGGTATTGAGTTAACCTACACTTGTGGCAACACACCAATCAACGGGAATGTCACCCAGTTCATGGACCGCCAATACACCTTTGGCGCACAGGGGCCCAATAACATCATGCTTTGGGTGGAGTCTGTGCTTGGCACGCACACTGGCAACAACAGAGTTTACAGCTCACAACCAGACACCGTGTCTGCCGCACTGCAGGGCCAGCCCTACAACATACCAGAGGGGTACATGGCCGTGTGGAATGTCAACGCGGACAGTGCTGACTTCCAAATAGGGCTGAGTCGCGATGGCTTCTTCGTCACTAACGGAGCCATTGGCACGTCCATAGCCATCTCAGAAGACACCACCTTCACTTACAATGGCATGTATTCCCTCACCACCCCTCTCATTGGACCAAGTGGGACAACAGGAAGGTCCATTCACAACACACGATGAGTTGGATCGCAGGTGCAATGCAGGGCGCTGGCCTGCTGGGTGACCTCGCAGGCACAATTGGACAAACAGTGTTGTACAATAAGCAGTTGAATATCACTAAATCTTTTAATCAGGCTCAGTTAGAGTTAGCCAAAGATCAAATGAAACAAAATCAACAGTCGGCTAACCAGTATTATGAATTTAATGCAAATTTGCCTGCCAATCAGTATAATAGTGCTGTCAGTGCTGGTCTTGATGCGGTTTCTGCACGTCAATTGGCTGGGTCGCACGAAGTGCGCTACTTTGGTGGACAACAAACACCAATGCTCCACCAAGGGCAAATGCAACAAATGATGTTCTCGTCAAAACACCTTATGCAAGCGCAAAACGTGCTTGGCACCTTTTCTCGTGGGACACCTGGTGTCACCCCCAGCAAAGGGCTGCCCAAACCACAGGGCATCACGTCCAGACCGCTCAGACCACAGGGTGTTTCAGCCACCATACCTGTGGCTGGAGCCACCACCACCAACAGCAGAGTGTGATTTTCTTTCCCTGGTTACGCGTACCGAACCTTACACAGTGTGGCGGCT
